# Synthetic glycan-based TLR4 agonists targeting caspase-4/11 for the development of adjuvants and immunotherapeutics†
†Dedicated to Prof. Paul Kosma on the occasion of his 65^th^ birthday.
‡
‡Electronic supplementary information (ESI) available: Supplementary figures and schemes, experimental details, characterization data and NMR spectra for new compounds. See DOI: 10.1039/c7sc05323a


**DOI:** 10.1039/c7sc05323a

**Published:** 2018-03-15

**Authors:** Florian Adanitsch, Jianjin Shi, Feng Shao, Rudi Beyaert, Holger Heine, Alla Zamyatina

**Affiliations:** a Department of Chemistry , University of Natural Resources and Life Sciences , Muthgasse 18 , A-1190 Vienna , Austria . Email: alla.zamyatina@boku.ac.at; b National Institute of Biological Sciences , Beijing 102206 , China; c Department for Biomedical Molecular Biology , Ghent University , Center for Inflammation Research , VIB , Ghent , Belgium; d Research Group Innate Immunity , Research Center Borstel , Leibniz Lung Center , Airway Research Center North (ARCN) , German Center for Lung research (DZL) , Borstel , Germany

## Abstract

Gram-negative bacterial lipopolysaccharide (LPS)-induced Toll-like receptor 4 (TLR4) mediated pro-inflammatory signaling plays a key role in immunoprotection against infectious challenges and boosts adaptive immunity, whereas the activation of the cytosolic LPS receptor caspase-4/11 leads to cell death by pyroptosis and is deeply implicated in the development of sepsis. Despite tremendous advances in the understanding of the LPS–TLR4 interaction, predictably regulated TLR4 activation has not yet been achieved. The structural basis for the induction of caspase-4/11 protease activity by LPS is currently unknown. The modulation of innate and adaptive immune responses through the controlled induction of TLR4 signaling without triggering caspase-4/11 activity would open novel perspectives in the development of safe vaccine adjuvants and immunotherapeutics. We report the discovery of highly potent glycan-based immunostimulants with picomolar affinity for TLR4 which interact with caspase-4/11 and promote caspase-4/11 oligomerization while abolishing caspase-11 protease activity. The rigidity and twisted molecular shape of the α,α-(1↔1′)-linked disaccharide core of synthetic LPS mimicking anionic glycolipids accounted for both species-independent and adjustable TLR4-mediated NF-κB signaling and the modulation of caspase-4/11 activation. By the use of crystal structure based design and advanced synthetic chemistry we created a set of versatile probes for studying the structural basis of caspase-4/11 activation and established a chemical strategy for controllable TLR4 mediated cytokine release which is dissociable from the induction of caspase-11 protease activity.

## Abstract 2

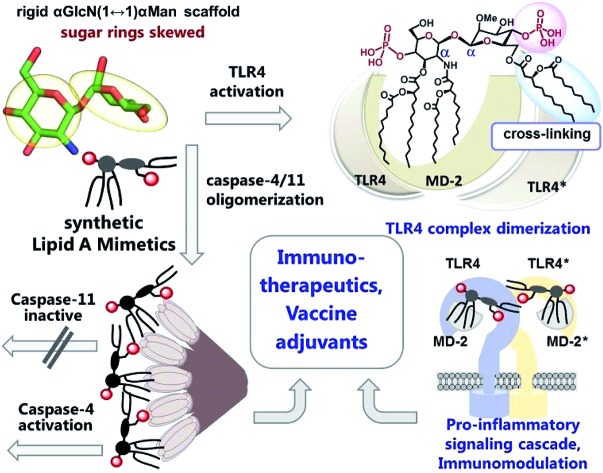
The skewed molecular shape of the rigid α,α-(1↔1′)-linked disaccharide core of novel synthetic anionic glycan-based immunostimulants is accountable for potent and adjustable TLR4-mediated signaling which is dissociable from the induction of caspase-11 protease activity.

## Introduction

**Fig. 1:**
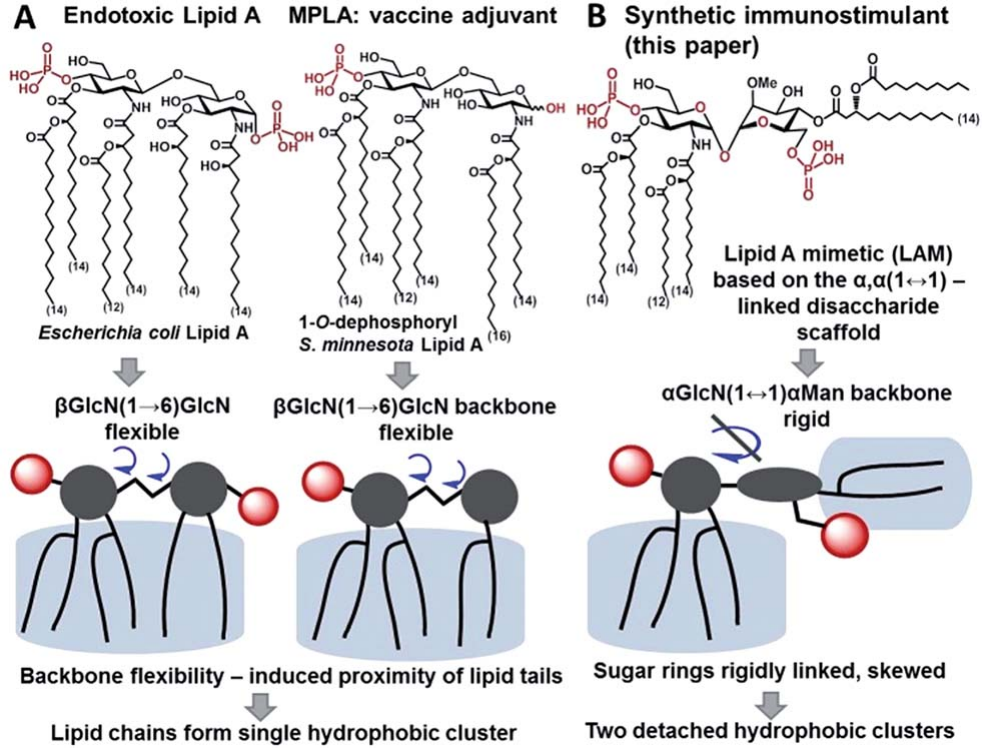
Chemical structure and schematic representation of (A) *E. coli* lipid A and vaccine adjuvant MPLA; (B) synthetic lipid A mimetics (LAMs) derived from the α,α-(1↔1′)-linked disaccharide scaffold.

Innate immunity provides an instant protection against bacterial infection by detecting and responding to lipopolysaccharide (LPS), a major component of the outer membrane of Gram-negative bacteria, which proceeds through a germline-encoded transmembrane pattern recognition receptor Toll-like receptor 4 (TLR4) (ESI-Fig. 1‡).^1^,^2^ TLR4 activation by LPS results in the induction of transcription factor NF-κB signaling leading to the upregulation of cytokines, chemokines and co-stimulatory molecules which generally facilitates recovery from infection. Dysregulated TLR4 signaling contributes to the pathogenesis of many chronic and acute inflammatory diseases such as asthma, arthritis, cardiovascular disorders, cancer, and sepsis syndrome which underscores the importance of the TLR4 complex as a therapeutic target.^3^–^6^ The modulation of TLR4-mediated signaling was demonstrated to confer protection against infectious challenges, to improve Alzheimer's disease-related pathology and to enhance recovery in cancer treatment.^7^–^9^ Besides, TLR4 activation potentiates both innate and adaptive immune responses,^10^ and therefore, TLR4 agonists can be applied as adjuvants for vaccine formulations aimed at infection and cancer that demand both humoral and Th1-biased immunity.^11^–^14^

The immunostimulating portion of LPS – a native TLR4 agonist glycophospholipid lipid A – is built on the basis of a highly conserved bisphosphorylated β(1→6)-linked diglucosamine (GlcN) backbone [βGlcN(1→6)GlcN] which carries a variable number of long-chain (*R*)-3-hydroxyacyl- and (*R*)-3-acyloxyacyl residues in asymmetric distribution (Fig. 1A). The TLR4-stimulating activity of LPS largely depends on the structure of lipid A, commonly, on its phosphorylation and acylation pattern (number, length and position of lipid chains), and varies from highly endotoxic (TLR4 agonist) to anti-inflammatory (TLR4 antagonist).^15^,^16^ Monophosphoryl lipid A (MPLA), a weak TLR4 agonist derived from *S. minnesota* LPS (Fig. 1A), was recently licensed as a vaccine adjuvant.^17^,^18^

Therapeutic immunomodulation has grown to be an attractive strategy for the treatment of acute and chronic conditions ranging from infectious diseases and sepsis to autoimmune disorders and cancer. Recent studies revealed that the inefficiency of the pro-inflammatory responses, in addition to hyperinflammation, is associated with the pathogenicity and progression of sepsis.^19^–^21^ It has also been recognized that the progression of auto-immune disorders as well as many cancers is related to non-resolving inflammation, which emphasizes TLR-mediated immunotherapy as a promising strategy for the treatment of a variety of diseases. The extensive application of protein subunit vaccines which necessitates a co-administration of immune-stimulating agents to boost cell-mediated and humoral immune responses as well as recent advances in the development of fully synthetic conjugated vaccines highlights the urgent need for novel TLR-dependent vaccine adjuvants.^22^–^24^

The therapeutic manipulation of the TLR4 system encounters significant challenges considering the enormous sensitivity of the TLR4 complex towards subtle variations in the chemical structure of lipid A, which exerts unsystematic effects on TLR4 activation. Further, species differences (human *vs.* mice) in ligand recognition by the TLR4 complex contribute to discrepancies in predicting the therapeutic effect using data obtained from mouse models. The recently discovered cysteine protease caspase-4/11 – a cytosolic LPS receptor which regulates the activation of the noncanonical NLRP3 inflammasome and causes a number of severe inflammatory impacts including the induction of the IL-1β signalling pathway and cell death by pyroptosis^25^–^28^ – significantly adds to the complexity of the pleiotropic effects of TLR4 agonists on the immune system. The induction of caspase-4 (or its mouse homologue caspase-11) protease activity and NLRP3 inflammasome activation are the crucial pathogenic factors in a variety of acute and chronic inflammatory settings including Alzheimer’s disease and sepsis.^29^–^32^

Deciphering the structural basis of LPS recognition by caspase-4/11 along with exploring the possibilities of dissecting TLR4 and caspase-4/11 activation pathways by molecularly defined ligands is crucial to foster the development of novel vaccine adjuvants and innovative immunotherapeutics targeted at the resolution of inflammation in preference to the inhibition of inflammatory responses.^20^,^33^ Besides, the emerging evidence on the benefits of eliciting a caspase-independent antitumor immunity through the induction of solely NF-κB signaling drives the creation of novel drugs satisfying these criteria.^34^

## Results and discussion

**Fig. 2:**
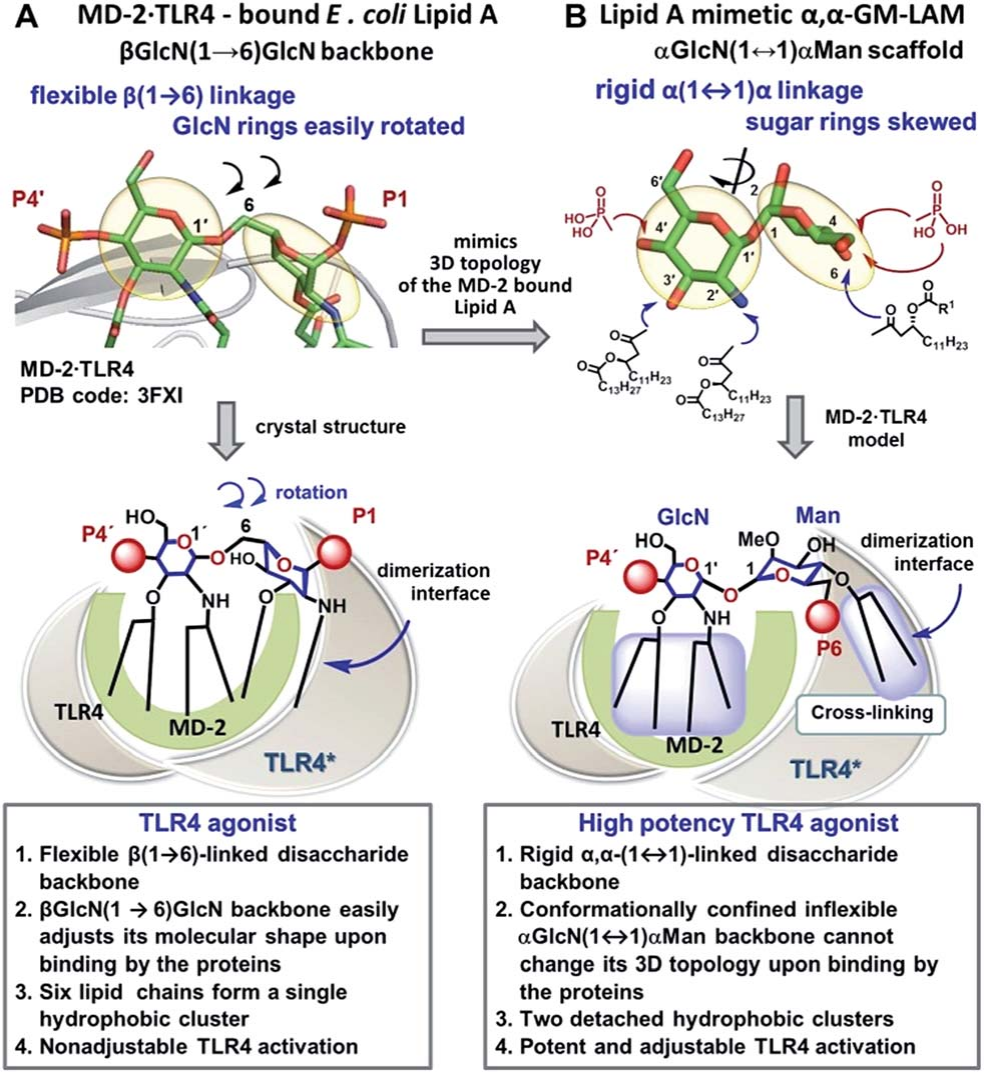
Crystal-structure-based design of TLR4 agonists and caspase-4/11 ligands based on the α,α-(1↔1′)-linked disaccharide scaffold. (A) Co-crystal structure of the MD-2·TLR4 bound lipid A (PDB code: 3FXI); (B) lipid A mimetics based on the trehalose-like α,α-(1↔1′)-linked disaccharide scaffold. Images were generated with PyMol.

Caspase-4/11 activation necessitates the binding of the lipid A terminus of LPS by the caspase activation and recruitment domain (CARD) resulting in caspase oligomerization.^25^ Only TLR4 stimulating LPS variants can activate inflammatory caspases, whereas native TLR4 antagonists such as lipid IVa and LPS from *R. sphaeroides* do not induce caspase-4/11 oligomerization and activation.^35^,^36^ The structural basis of caspase-4/11 activation is currently unknown, so that the design of caspase-4/11 specific ligands can exclusively rely on the TLR4–caspase-4/11 “cross-reactivity” of a particular lipid A variant. LPS-induced TLR4 activation requires specific fitting of the hexaacylated *E. coli* lipid A in a deep hydrophobic binding pocket of a co-receptor protein myeloid differentiation factor-2 (MD-2) which triggers the dimerization of two TLR4·MD-2·LPS complexes. The dimerization is driven by the interaction of the 2*N*-linked β-hydroxyacyl chain and the glycosidically linked phosphate group P1 of lipid A (PDB: ; 3FXI) with the second TLR4* complex (Fig. 2A).^37^,^38^ Receptor complex dimerization initiates the recruitment of adaptor proteins to the intracellular TIR (Toll/interleukin-1 receptor) domains of TLR4 ultimately resulting in the activation of downstream signaling pathways which induce the upregulation of cytokines, chemokines and co-stimulatory molecules.^1^,^2^

Despite the tremendous efforts of Pharma R&D to develop TLR4-dependent agonists as drugs and vaccine adjuvants, predictably regulated TLR4 activation has not yet been achieved. Notwithstanding, the moderate activity and low toxicity of a vaccine adjuvant MPLA were attributed to the inefficiency of MPLA-driven TLR4 complex dimerization.^39^

**Fig. 3:**
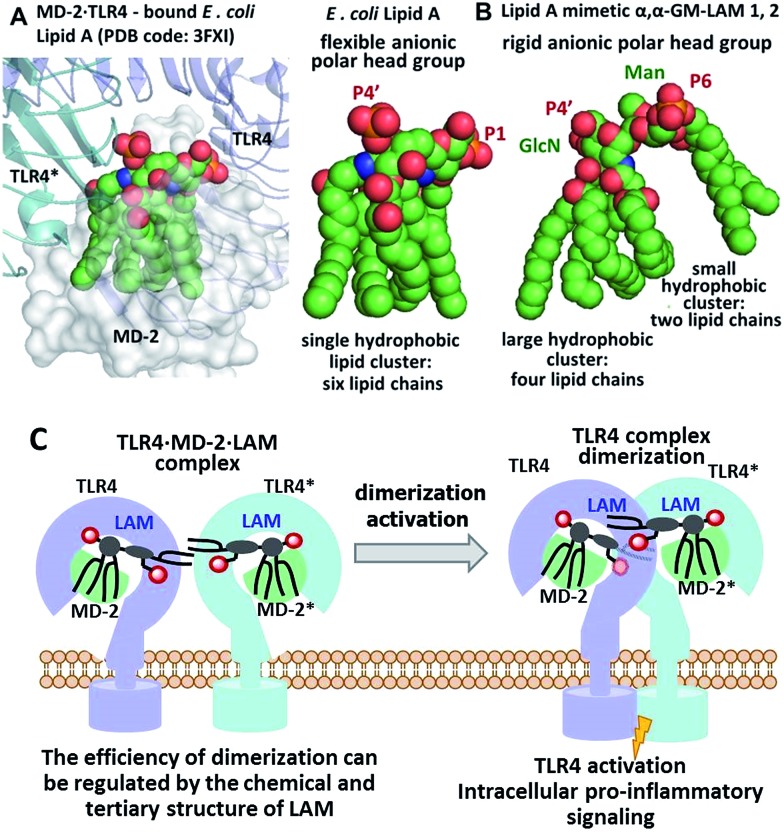
(A) molecular shape of TLR4·MD-2 – bound *E. coli* lipid A (PDB: ; 3FXI); (B) molecular shape of synthetic lipid A mimetic **1**; (C) proposed mode of TLR4 complex dimerization and activation by LAMs. Images were generated with PyMol.

We hypothesized that the predictable and adjustable TLR4-mediated modulation of NF-κB signaling can be attained by a tight regulation of the efficiency of the dimerization of [TLR4·MD-2·ligand] complexes. This could be achieved by an artificial ligand that selectively binds to the hydrophobic pocket of MD-2·TLR4 and simultaneously crosslinks the second MD-2*·TLR4* complex by virtue of hydrophobic and ionic interactions. Since a number of other members of the TLR family (*e.g.*, TLR2/1, TLR2/6, and TLR3) are activated through a crosslinking-induced dimerization by their putative ligands,^40^,^41^ a similar strategy could also be accomplished for the controlled activation of TLR4 (Fig. 3).

Thus, the “ideal” TLR4 agonist should be composed of two separate hydrophobic clusters – one large lipid cluster that can occupy the deep hydrophobic binding pocket of MD-2 42 and a smaller solitary hydrophobic bunch which can get exposed on the surface of MD-2 and induce dimerization with the second TLR4* complex (Fig. 1B, 2B). Both hydrophobic clusters should be attached to a sugar-derived hydrophilic poly-anionic head group which will be involved in protein binding *via* ionic interactions with multiple Arg and Lys residues at the rim of the binding pocket of MD-2.^43^

To fulfill these criteria, we could not rely on the flexible three-bond linked βGlcN(1→6)GlcN backbone of native lipid A which can spontaneously adjust its molecular shape (*i.e.* the relative orientation of the β(1→6)-linked GlcN rings) upon binding by the proteins (Fig. 1A and 2A). The flexibility of the carbohydrate backbone of lipid A would allow for the proximity-induced “sticking” of multiple aliphatic lipid chains attached to both GlcN residues which will form a single hydrophobic cluster (Fig. 3A). Instead, we built our hybrid TLR4 and caspase-4/11 ligands on the basis of an exceptionally rigid α,α-(1↔1′)-linked trehalose-like disaccharide scaffold characterized by a skewed relative arrangement of two pyranose rings which mimics the 3D tertiary structure of the β(1→6)-linked diglucosamine backbone MD-2·TLR4 bound lipid A (Fig. 2).^44^ The skewed 3D topology of α,α-trehalose relies on the favored *gauche*–*gauche* conformation with respect to the values of torsion angles about the α,α-(1↔1′) glycosidic linkage which is governed by the anomeric effect and is only marginally dependent on the nature of functional groups.^45^,^46^

Thus, the twisted molecular shape of the synthetic trehalose-like αGlcN(1↔1′)αMan scaffold allowed for dissecting two hydrophobic clusters in our hybrid TLR4 agonist molecules: one large hydrophobic batch composed of two (*R*)-3-acyloxyacyl residues attached at positions 2′ and 3′ of the α-GlcN ring (corresponding to the acylation pattern of *E. coli* lipid A) and a smaller detached hydrophobic cluster composed of an (*R*)-3-acyloxyacyl residue linked at positions 4- or 6- of the α-Man moiety (Fig. 2B, 3B).

**Fig. 4:**
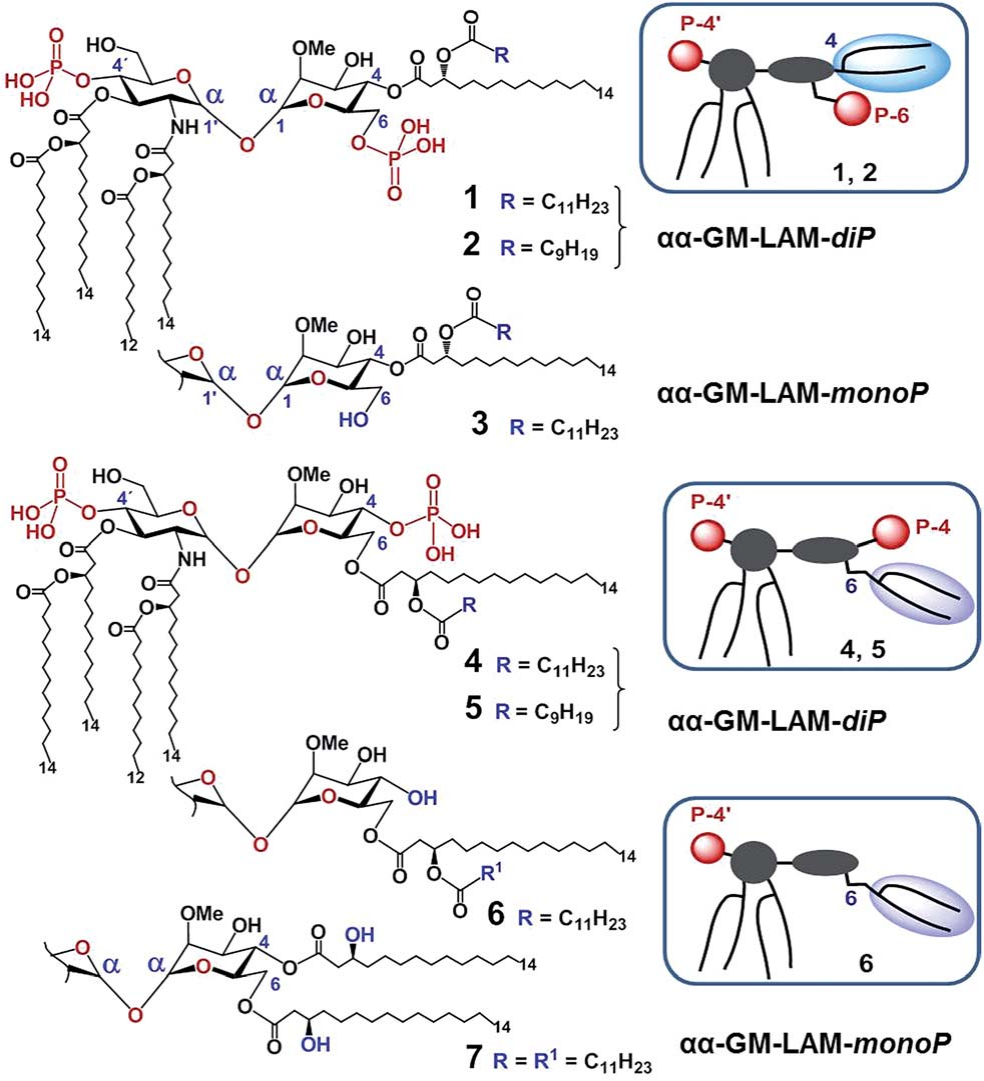
Structure of αα-GM-LAMs based on the αGlcN(1↔1′)αMan scaffold. Compounds **1**, **2**, **4**, and **5** are diphosphates (αα-LAM-*diP*), and compounds **3**, **6**, and **7** are monophosphates (αα-LAM-*monoP*). Schematic representation of the molecular shape of αα-GM-LAMs is given in frames.

According to our model, the four lipid chains attached to the 4′-phosphorylated GlcN ring (large hydrophobic cluster) will be intercalated into the leucine-rich binding pocket of MD-2, whereas the Man moiety comprising two lipid chains (smaller hydrophobic cluster) and a phosphate group will be excluded from the binding pocket and exposed on the surface of MD-2 to induce cross-linking with the second TLR4*·MD-2* complex (Fig. 2B, 3C). To gain insight into the structural requirements responsible for TLR4 and caspase-4/11 activation, seven variably acylated and differently phosphorylated αGlcN(1↔1′)αMan-based lipid A mimetics (thereafter αα-GM-LAMs) were synthesized (Fig. 4).

The glycosylation reaction toward non-reducing α,α-(1↔1′)-linked disaccharides is challenging since the formation of four glycosidically linked products can be expected and, thereby, a simultaneous stereocontrol at two anomeric centers is required.^47^ The synthesis of αα-GM-LAMs demands the application of a set of at least seven orthogonal protecting groups which allows for the installment of multiple functionalities entailed in the target molecules. Since the nature of protecting groups exerts enormous influence on the glycosylation outcome with respect to both stereoselectivity and efficiency,^48^,^49^ the stereocontrol of chemical α,α-1,1′-glycosylation is especially demanding in this particular case.

**Scheme 1:**
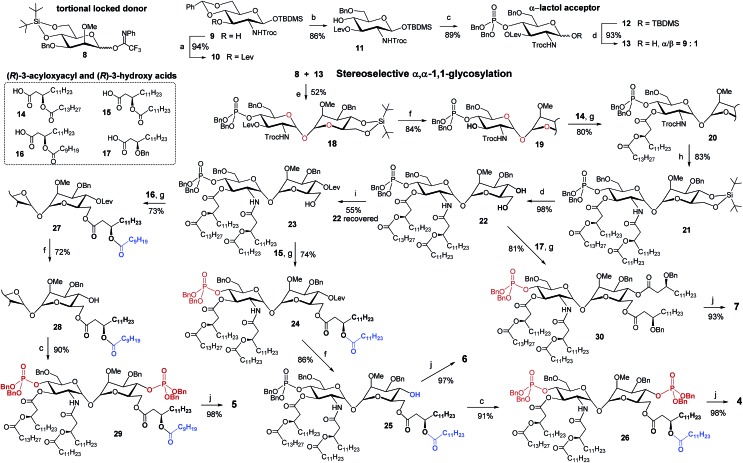
Synthesis of the orthogonally protected αGlcN(1↔1′)αMan scaffold **18** and αα-GM-LAMs **4–7**. Reagents and conditions: (a) LevOH, DIC, DMAP, DCM, 0 °C to rt; (b) Et_3_SiH, (CF_3_CO)_2_O, CF_3_COOH, DCM, 0 °C; (c) (1) (BnO)_2_PN(*i*Pr)_2_, 1*H*-tetrazole, DCM, rt; (2) *m*CPBA, DCM, –78 °C; (d) HF-Py, THF; (e) TMSOTf, MS 4 Å, DCM, 0 °C; (f) H_2_NNH_2_–H_2_O, Pyr, AcOH, rt; (g) DIC, DMAP, DCM, 0 °C; (h) (1) Zn, AcOH, DCM, rt, (2) **15**, EDC, CHCl_3_, rt; (i) LevOH, DIC, DMAP, DCM, 0 °C; (j) H_2_, Pd black, toluene–MeOH.

The stereoselective synthesis of the fully orthogonally protected αGlcN(1↔1′)αMan scaffold **18** was achieved by the glycosylation of the *N*-carbamate α-lactol acceptor **13** by the torsional locked Man-derived imidate donor **8** (Scheme 1). The presence of a carbamate NH able to form a hydrogen bond with an axial oxygen at C-1 enabled the enhancement of the *α*/*β*-ratio (*α*/*β* = 9 : 1) of the GlcN hemiacetal acceptor **13**.^50^ To match the reactivity of the glycosyl donor with the nucleophilicity of the α-lactol acceptor, the electron-donating effect of the *N*-Troc group in **13** was balanced by the electron-withdrawing influence of the 3-*O*-levulinoyl ester group. A TMSOTf-promoted glycosylation of **13** by the *N*-phenyl-trifluoroacetimidate^51^ donor **8** afforded α,α-1,1′-linked **18** in 52% yield (comprehensive explanation of glycosylation approaches is provided in the ESI‡). Sequential deprotection of 3′-hydroxyl and 2′-amino groups combined with successive acylation by (*R*)-3-acyloxyacyl fatty acids of variable lengths (**14–16**) furnished tetra-acylated **21**. Cleavage of the 4,6-*O-tert*-butylsilylene protecting group afforded a key diol **22**. Relying on the conformational constraints around the α,α-1,1′ glycosidic linkage as well as on the specific molecular shape of **22**, we could regioselectively protect the secondary 4-OH group as the levulinoyl ester which gave **23** having a free primary hydroxyl group at position 6. The divergent synthetic route^52^ (detailed description of synthetic approaches is provided in the ESI‡) gave rise to variably functionalized target lipid A mimetics, αα-GM-LAMs **1–7**.

**Fig. 5:**
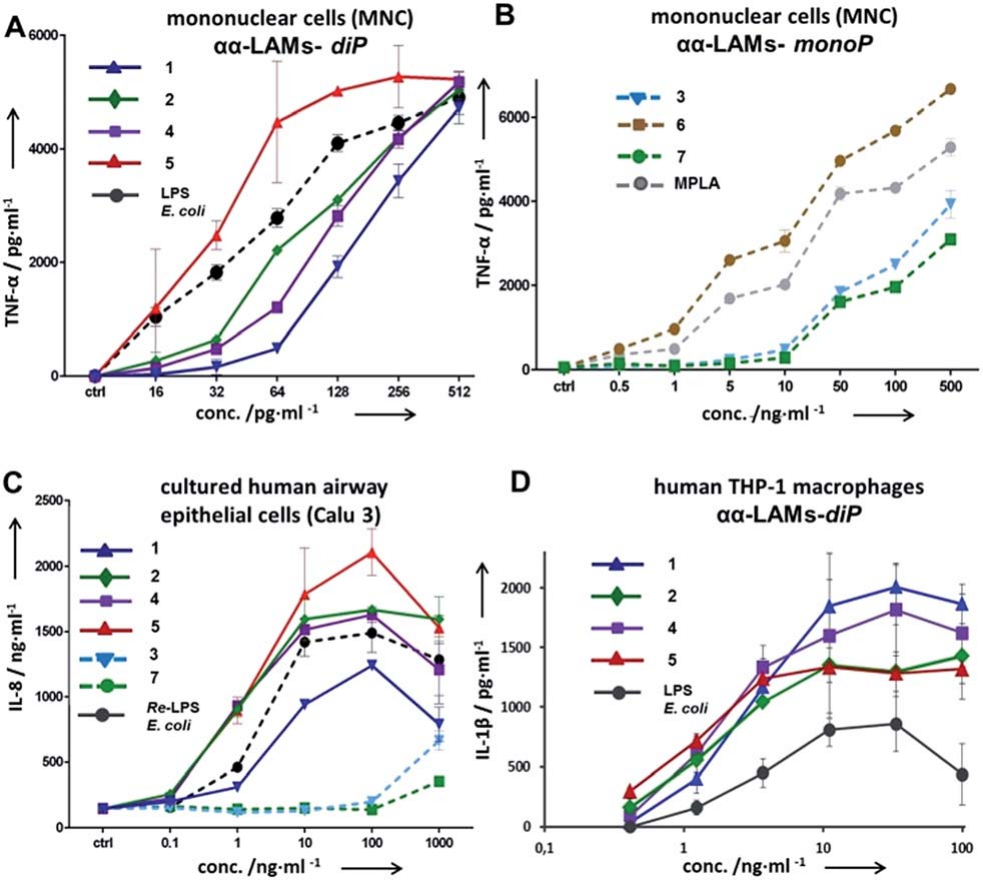
Dose-dependent expression of cytokines in human cell lines induced by αα-LAMs **1–7**. (A) Expression of TNF-α in hMNCs induced by αα-LAM diphosphates **1**, **2**, **4**, and **5**; (B) expression of TNF-α in hMNCs by αα-LAM monophosphates **3**, **6**, and **7**; (C) production of IL-6 in cultured human airway epithelial cells; (D) expression of IL-1β in the hTHP-1 macrophage cell line.

The application of αα-GM-LAMs **1–7** to hTLR4 transfected HEK293 cells confirmed their potent cytokine-inducing activity in a TLR4-dependent manner (ESI-Fig. 2‡). Picomolar concentrations of αα-GM-LAM-*diP***1**, **2**, **4**, and **5** initiated the expression of tumor necrosis factor-α (TNF-α), interleukin-6 (IL-6) and monocyte chemotactic protein-1 (MCP1) in human mononuclear cells (MNCs) and human monocytic macrophage-like cell line THP-1 in a dose-dependent manner (Fig. 5A, ESI-Fig. 3‡). The NF-κB signaling induced by αα-LAMs could be readily and predictably modulated through the modification of the chemical structure *via* switching the sites of attachment (position 4 or 6) of the (*R*)-3-acyloxyacyl chains and phosphate group at the Man moiety, as well as by altering the phosphorylation status of the disaccharide backbone and the length of the secondary acyl chains facing the dimerization interface. 6-*O*-phosphate LAMs **1** and **2** exhibited lower activating potency compared to the corresponding 4-*O*-phosphates **4** and **5**, whereas the shortening of the secondary lipid chain at the Man moiety (LAMs **2** and **5**) allowed for the enhancement of cytokine-inducing capacity, which confirmed an apparent impact of hydrophobic interactions at the TLR4·MD-2·LAM dimerization interface. The monophosphate counterparts, αα-GM-LAM-*monoP***3**, **6** and **7**, performed as weak TLR4 agonists, showing a similar tendency toward higher activity for the 6-*O*-lipidated analogue (compound **6**) which was more potent than αα-LAM **3** and MPLA in inducing a TNF-α response in MNCs (Fig. 5B). Thus, the absence of a phosphate group at the sugar residue facing the dimerization interface allowed for reduced cytokine production, probably, due to the less efficient dimerization of TLR4–ligand complexes.

The cytokine inducing activity in human airway epithelial cells was similarly correlated with the presence and the position of the phosphate group on the Man moiety (Fig. 5C). Monitoring the secretion of IL-1β in the human monocytic macrophage-like cell line THP-1 revealed an inverted activation profile, with αα-GM-LAM **1** being the most potent at higher concentrations, indicating an involvement of different structural factors in ligand recognition for the induction of the IL-1β pathway (Fig. 5D). The observed TLR4 activating effects were species-independent since αα-GM-LAMs induced the production of TNF-α in bone marrow derived mouse macrophages (BMDM) with similar efficiency to that in human cells (ESI-Fig. 4‡). In all instances, αα-GM-LAM-*diP* were powerful similar to LPS (20 kDa heterogeneous glycan) in inducing robust NF-κB signaling at picomolar concentrations, which was though predictably regulated by specific chemical modifications.

**Fig. 6:**
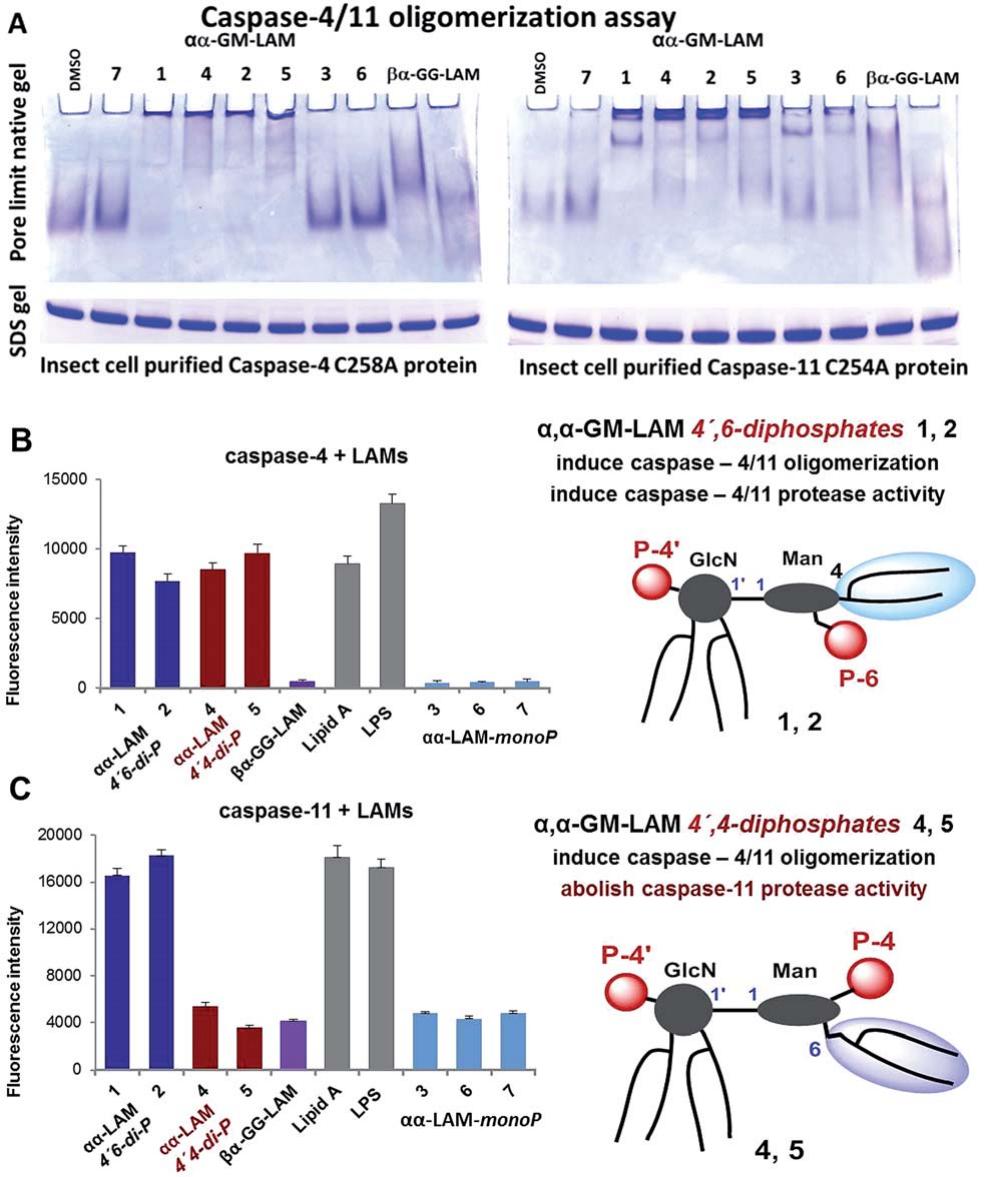
(A) αα-GM-LAM-*diP***1**, **2**, **4** and **5** binding induces the oligomerization of caspase-4/11 as analysed by pore-limit native gel electrophoresis; (B) induction of caspase-4 activation by LAMs; (C) induction of caspase-11 activation by LAMs.

Bisphosphorylated LAMs **1**, **2**, **4** and **5** induced the efficient oligomerization of the caspase-4/11 protein (Fig. 6A) through, most probably, direct binding to the caspase CARD domain (ESI-Fig. 5‡).^25^ In contrast, αα-GM-LAM-*monoP***3** and **6** only weakly interacted with caspase-4/11 as revealed by pore-limit native gel electrophoresis. All four diphosphate LAMs **1**, **2**, **4** and **5** induced caspase-4 protease activity similarly to *E. coli* lipid A and LPS (Fig. 6B). Although the most powerful TLR4 agonists αα-GM-LAM-*diP***4** and **5** induced the oligomerization of caspase-11, in contrast to *E. coli* LPS and α,α-GM-LAMs **1** and **2**, they did not promote caspase-11 protease activity (Fig. 6C). This intriguing finding suggests that caspase-11 oligomerization, in contrast to previous beliefs, is not a prerequisite for its catalytic activation. Since LPS mimetics **4** and **5** are based on the same rigid α,α-(1↔1′)-linked disaccharide scaffold as LAMs **1** and **2**, but differ in the sites of attachment of the phosphate group and the lipid chains at the Man moiety, the overall tertiary structure of the ligand could be decisive for proper binding to the CARD which regulates caspase-11 activation.

Our synthetic TLR4 antagonists, the tetraacylated lipid A mimetics derived from the βGlcN(1↔1′)αGlcN scaffold having a planar topology (βα-GG-LAM),^50^,^53^ induced the formation of smaller aggregates, probably dimers, with both caspases and failed to provoke caspase-4/11 activation. Thus, apart from being perfect therapeutic candidates, LAMs act as versatile probes for studying the structural basis of caspase-4/11 activation.

## Conclusions

We report the crystal structure based design, synthesis and functional studies of glycan-based immunomodulators which induce potent and controllable species-independent (human- and mouse-) TLR4 activation rationalized by the rigid skewed topology of their α,α-(1↔1′)-linked disaccharide backbone and a ligand–protein conformational selection. The molecular shape of LAMs is decisive for both the picomolar affinity for TLR4·MD-2 and predictable modulation of the TLR4-mediated expression of cytokines which can be attained by chemical modifications. LAMs are the first structurally defined synthetic molecules which target inflammatory caspases, thereby offering unique tools for studying the structural basis of caspase-4/11 activation. We provide the first evidence of potent TLR4-mediated NF-κB signaling which is dissociable from the induction of caspase-11 protease activity and associated toxic effects. Lipid A mimetics **4** and **5** are the first powerful TLR4 agonists able to interact with inflammatory caspases, induce caspase-4/11 oligomerization and concurrently abolish caspase-11 protease activity. This finding is of immense importance for the development of novel drugs that target innate immune receptors to cure infectious diseases and inflammation or serve as vaccine adjuvants and immunotherapeutics.

## Conflicts of interest

There are no conflicts to declare.

## Supplementary Material

Supplementary information
