# Supplementary material for: Synthetic glycan-based TLR4 agonists targeting caspase-4/11 for the development of adjuvants and immunotherapeutics
Source: Chem Sci. 2018 Mar 15;9(16):3957–63. doi: 10.1039/c7sc05323a (PMC5941199; doi:10.1039/c7sc05323a)

Electronic Supplementary Material (ESI) for Chemical Science.
This journal is © The Royal Society of Chemistry 2018

This version of the Supplementary Information uploaded on 25th April 2018

Synthetic glycan-based TLR4 agonists targeting caspase-4/11 for the development of adjuvants and immunotherapeutics

Florian Adanitsch, Jianjin Shi, Feng Shao, Rudi Beyaert, Holger Heine and Alla Zamyatina*

Supporting Information

Content

Supplementary Figures 3

Supplementary SI-Figure 1 3

Supplementary SI-Figure 2 4

Supplementary SI-Figure 3 5

Supplementary SI-Figure 4 7

Supplementary SI-Figure 5 8

Synthesis of immunostimulatory Lipid A mimetics (αα-GM-LAMs) 9

Supplementary Scheme 1. 9

Glycosylation reaction towards α(1↔1′)α-linked pyranosides 10

Synthesis of αGlcN(1↔1′)αMan scaffold 11

Synthesis of Lipid A mimetics (LAMs) based on the αGlcN(1↔1′)αMan scaffold. 12

Experimental procedures 13

Experimental Procedures: Biological assays 13

Solubilization of synthetic compounds, preparation of test samples. 13

Stimulation of transient hTLR4/MD-2/CD14 transfected HEK293 cells. 13

Assay in human mononuclear cells (MNC). 13

Assay in human bronchial epithelial cells. 14

Differentiation and stimulation of THP-1 cells. 14

Stimulation of bone marrow-derived mouse macrophages (BMDM). 14

Caspase-4/11 oligomerization analyses by pore-limit native gel. 14

Caspase-4/11 activity measurement. 15

Experimental Procedures: Synthesis 16

General synthetic methods 16

Synthesis of 10 17

Synthesis of 11 17

Synthesis of 12 18

Synthesis of 13 18

Synthesis of 14-17 19

Synthesis of 18 19

Synthesis of 19 20
Synthesis of 20 21
Synthesis of 21 22
Synthesis of 22 23
Synthesis of 23 24
Synthesis of 24 25
Synthesis of 25 26
Synthesis of 26 27
Synthesis of 27 28
Synthesis of 28 29
Synthesis of 29 30
Synthesis of 30 31
Synthesis of 4 32
Synthesis of 5 32
Synthesis of 6 33
Synthesis of 7 34
References 34
NMR and Mass- spectra 36
^{1}H and ^{13}C NMR spectra of target compounds and synthetic intermediates 36
HSQC-NMR and MALDI-TOF spectra of target compounds 36

Supplementary Figures

Supplementary SI-Figure 1

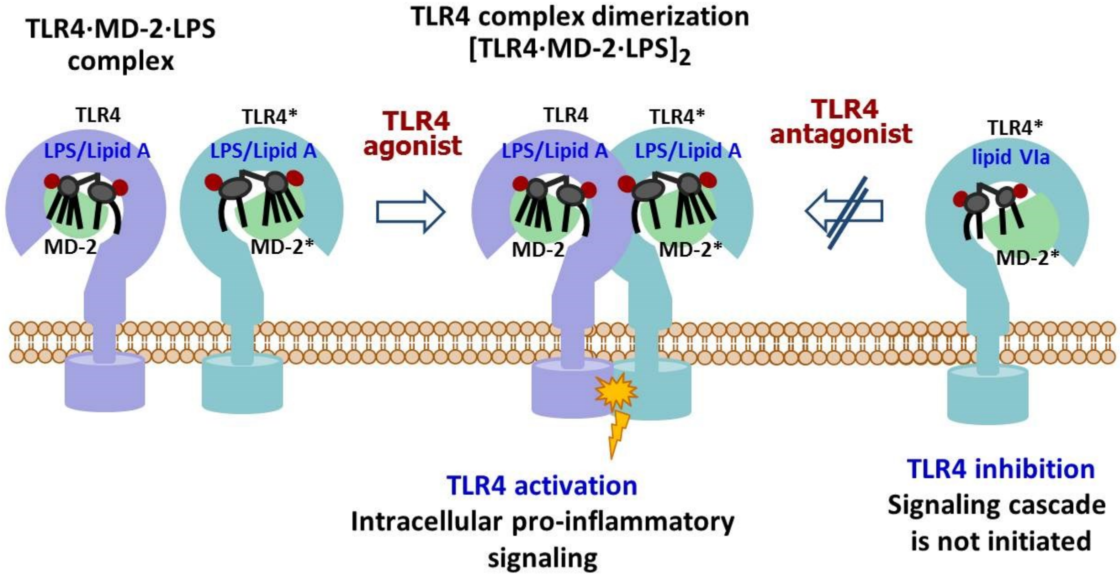

SI-Figure 1A. Schematic representation of the LPS-induced TLR4 complex dimerization. Binding of hexa-acylated Lipid A of *E. coli* results in the dimerization of two TLR4·MD-2·LPS complexes which, in turn, triggers the intracellular pro-inflammatory signaling cascade. The binding of underacylated Lipid A variants such as tetra-acylated lipid IVa blocks the binding pocket of MD-2 without inducing dimerization. Image was generated with ChemDraw and PowerPoint.

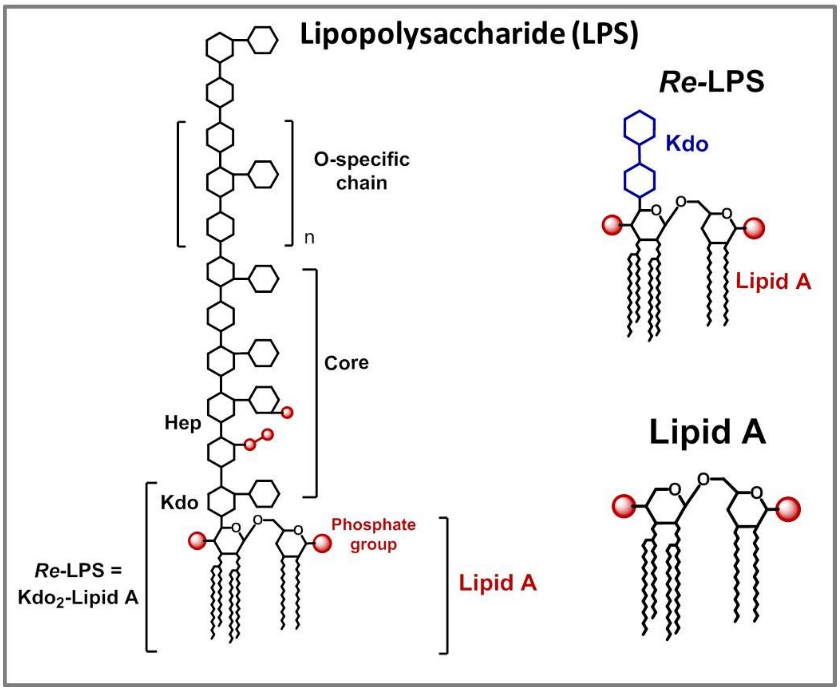

SI-Figure 1B. Schematic structure of LPS, Lipid A and *Re*-LPS (Kdo_2-Lipid A). Image was generated with ChemDraw.

Co-crystal structure of binary TLR4·MD-2·*Ra*-LPS complex (PDB Code: 3FXI)

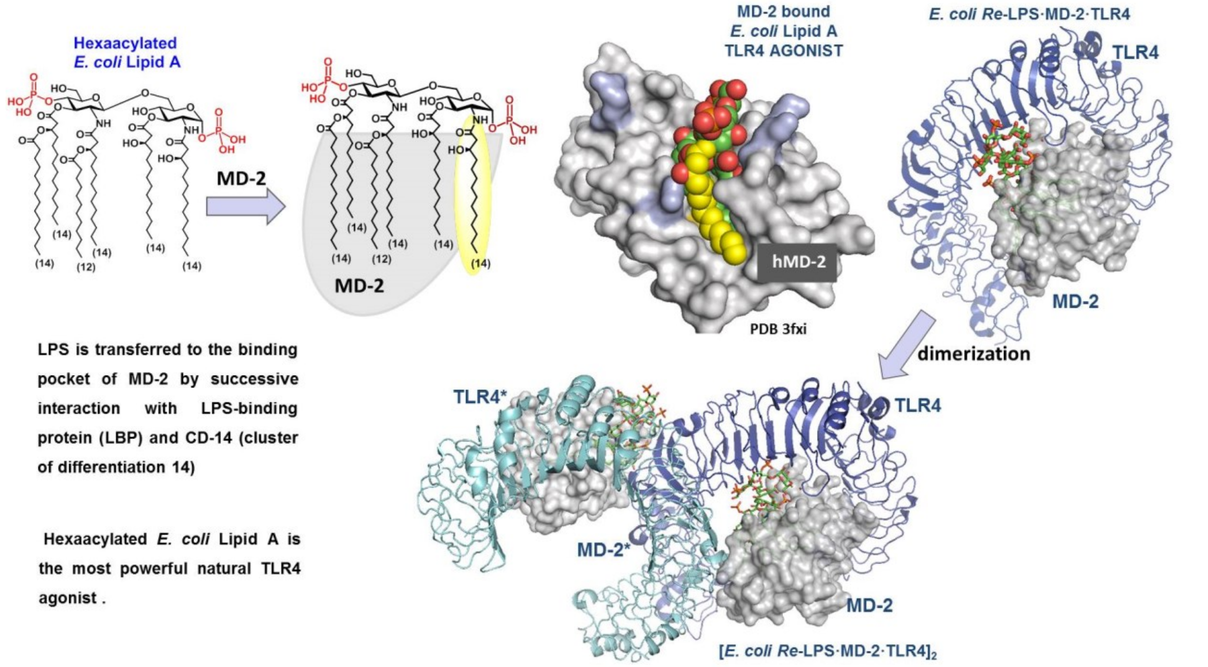

SI-Figure 1C. Structural basis of TLR4 complex dimerization induced by *E. coli* LPS.
Hexa-acylated *E. coli* Lipid A selectively binds to the hydrophobic binding pocket of MD-2 (top) so that the five lipid chains are inserted into the binding cleft of MD-2 and the 6th lipid chain (highlighted in yellow) is exposed on the surface of the protein (PDB code: 3FXI). The polar head group (1,4'-bisphosphorylated βGlcN(1→6)GlcN) is situated above the pocket with the phosphate groups interacting with Lys and Arg residues at the entrance of the binding pocket of MD-2 (only Lipid A portion of LPS is shown for clarity). The exposed lipid chain and the phosphate group at position 1 of the proximal GlcN residue promote dimerization with the second TLR4*·MD-2*·*Ra*-LPS complex (bottom). Image was generated with ChemDraw, PyMol and PowerPoint.

Supplementary SI-Figure 2

The ability of the αα-GM-LAMs **1-7** to induce the TLR4-mediated signaling was first examined in hTLR4/hMD-2/hCD14 transfected human embryonic kidney (HEK)293 cells. The TLR4 activation by compounds **1 - 7** was tested over a wide concentration range (0.01 – 1000 ng/mL) by monitoring the activation of NF-κB regulated signal transduction pathway via measuring the induction of IL-8 expression and compared to responses elicited by *E. coli* LPS (for αα-GM-LAM-*diP* **1, 2, 4, 5**) and by MPLA (for αα-GM-LAM-*monoP* **3, 6** and **7**), respectively.

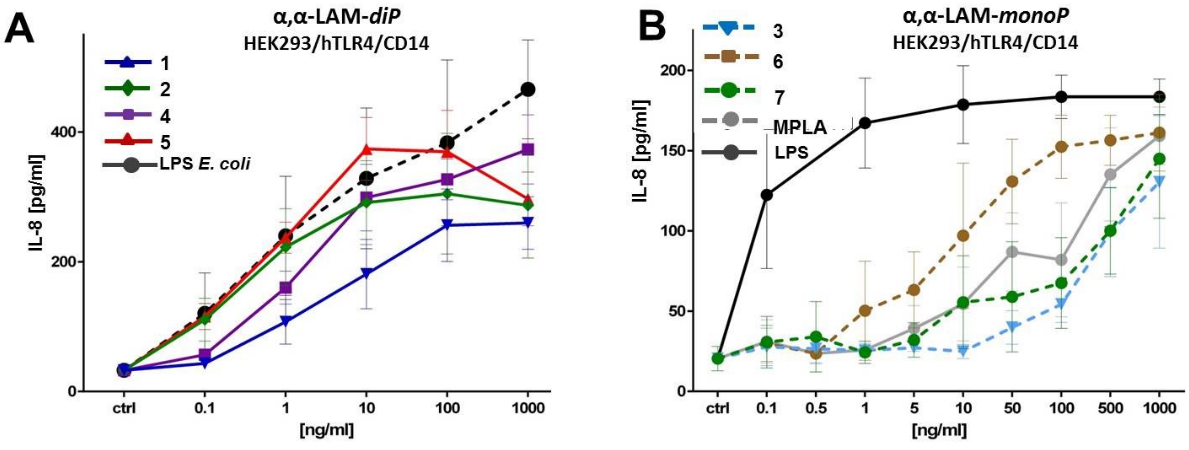

SI-Figure 2. Dose-dependent activation of TLR4 signaling (read-out IL-8) in hTLR4/hMD-2/hCD14 transfected HEK293 cells by (**A**) Lipid A mimetic diphosphates (αα-GM-LAMs-*diP*) **1, 2** and **4, 5** compared to *E. coli* LPS and by (**B**) Lipid A mimetic monophosphates (αα-GM-LAMs-*monoP*) **3, 6, 7** compared to MPLA.

Supplementary SI-Figure 3

The skewed molecular shape of the non-reducing αGlcN(1↔1′)αMan scaffold and the exceptional rigidity of the αα-1,1′ glycosidic linkage are decisive for appropriate fitting of the LAM ligand into the binding pocket of MD-2. According to our model, the four lipid chains (acylation pattern of *E. coli* Lipid A) attached to the planar oriented GlcN ring are inserted into the deep hydrophobic binding groove of MD-2, whereas a twistedly oriented Man ring is exposed on the surface of MD-2. The two protruding lipid chains linked to the Man moiety ((*R*)-3-acyloxyacyl chains at position 4 or 6) and a phosphate group attached at the Man moiety (at position 4 or 6) are available for cross-linking and binding the second TLR4*·MD-2*·LAM receptor complex.

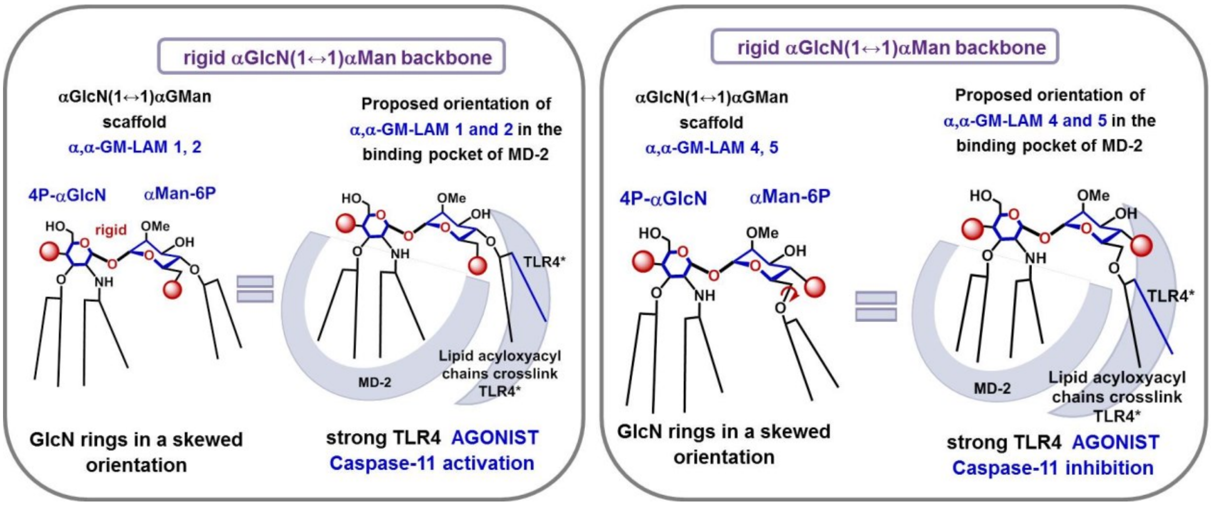

Exceptional rigidity of the αα-1,1′-linkage would not allow for a conformational adjustment of the disaccharide backbone of LAM to the shape of the binding pocket of the receptor protein. In contrast, the native TLR4 ligand Lipid A, which is built on the basis of extremely flexible three-bond β(1→6)-linked diglucosamine, readily adjusts its 3D-molecular shape to facilitate the fitting into the binding pocket of MD-2. This unique feature of LAMs allows for a possibility to regulate the effectiveness and the tightness of TLR4 complexes dimerization by varying the number and length of lipid chains exposed at the dimerization interface as well as by varying the position of the phosphate group and acyloxyacyl chains at the Man moiety (positions 4 and 6) which are presented at the site of contact with the second receptor complex. Thus, the interexchange of phosphate groups and lipid chains between positions 4 and 6 at the Man moiety was expected to result in different binding properties and, therefore, to predictably modulate the intracellular signaling. Importantly, controllable and predictable modulation of the TLR4-mediated signaling could not been achieved by use of either natural Lipid A variants or by synthetic Lipid A analogues which are generally based on the inherently flexible βGlcN(1→6)GlcN backbone or even more flexible GlcN - linear aglycon combinations.[1-4]

To test the impact of LAMs diphosphates **1, 2** and **4, 5** and LAMs monophosphates **3, 6** and **7** on the induction of NF-κB signaling, the release of IL-6 (in addition to release of TNF-α, main text) in human MNC was examined (SI-Figure 3 A). The expression of IL-6 induced by LAMs diphosphates αα-GM-LAMs-*diP* could be efficiently modulated by varying the position of the phosphate group at the Man moiety revealing that the 4-phosphates **4** and **5** are more active than the corresponding 6-phosphates **1** and **2** (compounds possessing lipid chains of equal length are compared). Shortening of the secondary acyl chain (C_{10} vs. C_{12}) resulted in substantial

enhancement of the IL-6 release: compounds **2** and **5** (which expose a C_{10} secondary lipid chain at the dimerization interface) were more efficient in inducing the expression of IL-6 than the corresponding LAMs **1** and **4** with shorter secondary lipid chain (C_{12}); (compounds having equal phosphorylation pattern are compared).

Dampened induction of IL-6 release by LAMs monophosphates (αα-GM-LAMs-*monoP*) correlates with their chemical structure missing a phosphate group at the Man moiety which confirms the importance of ionic interactions at the dimerization interface (SI-Figure 3 B). The modulation of TLR4 activation by αα-GM-LAMs-*monoP* could be achieved through varying the position and the length of lipid chains at C-4 and C-6 of the Man moiety. LAM **6** with acyloxyacyl chain at position 6 at the Man residue revealed the highest potency in inducing expression of IL-6 and was more active than MPLA and compounds **3** (Man-4-*O*-acyloxyacyl) and **7** (Man-4,6-di-*O*-hyroxyacyl).

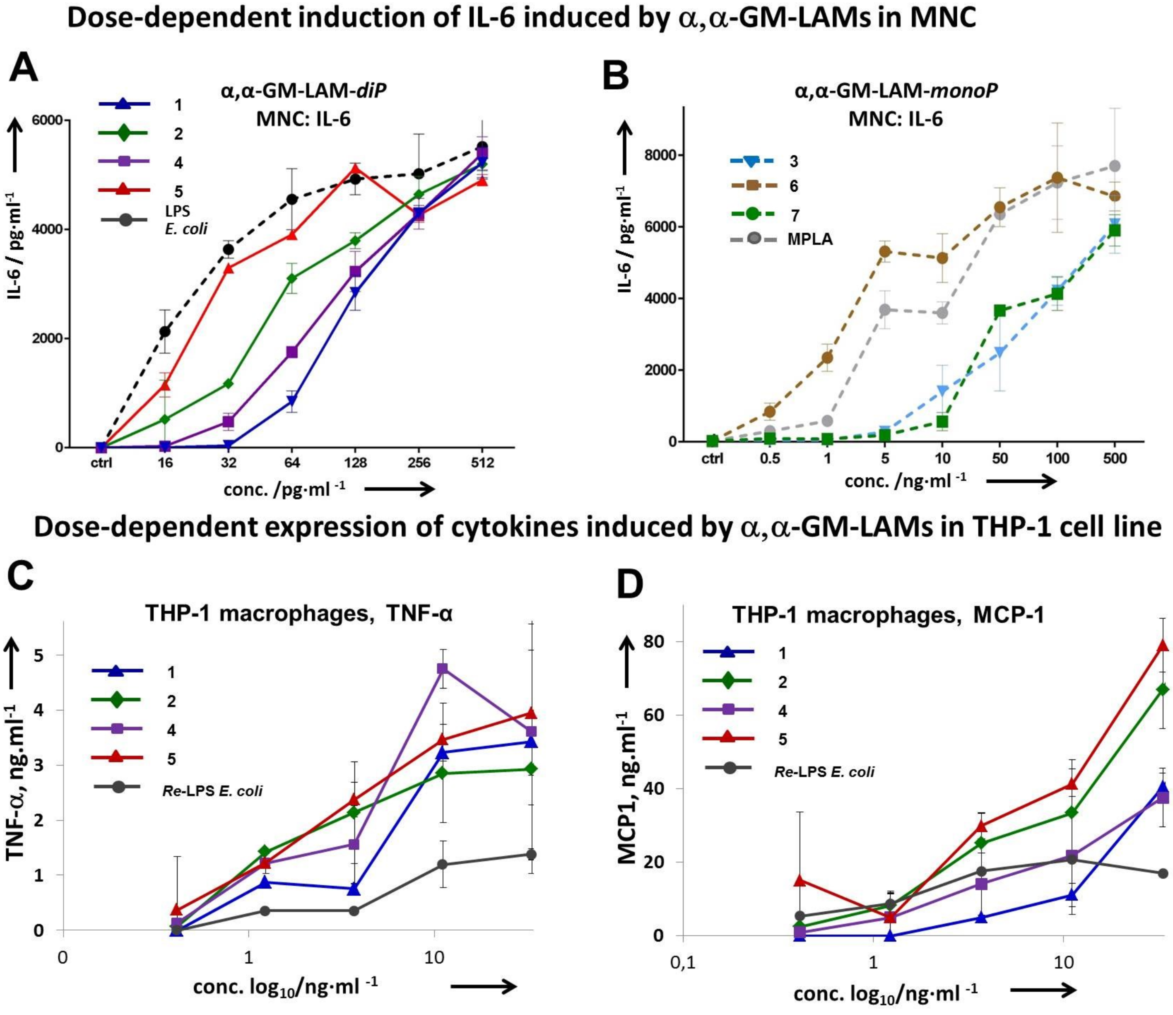

SI-Figure 3. Expression of cytokines induced by αα-GM-LAMs in human mononuclear cells (MNC) and human monocytic macrophage-like cell line THP-1. (**A**) Induction of IL-6 release by αα-GM-LAMs-*diP* **1, 2, 4** and **5** compared *E.coli* LPS in MNC. (**B**) Expression of IL-6 induced by monophoshorylated αα-GM-LAMs-*monoP* **3, 6** and **7** compared MPLA in MNC; (**C**) Dose-dependent expression of TNF-α induced by αα-GM-LAM-*diP* **1, 2, 4** and **5** in THP-1 cell line at the concentration range 0.4 – 300 ng/ml compared to *E. coli Re*-LPS (Kdo$_2$-Lipid A); (**D**) Dose-dependent expression of MCP1 induced by αα-GM-LAM-*diP* **1, 2, 4** and **5** in THP-1 cell line at the concentration range 0.4 – 300 ng/ml compared to *E. coli Re*-LPS (Kdo$_2$-Lipid A).

LAMs diphosphates **1, 2** and **4, 5** were examined for the ability to initiate the expression of tumor necrosis factor-α (TNF-α) and monocyte chemotactic protein-1 (MCP-1) in human monocytic macrophage-like cell line THP-1 which expresses MD-2, CD14 and a variety of cell surface receptors including TLR4. The dose-dependent stimulating activity of αα-GM-LAMs-*diP* was cytokine-specific revealing higher potency in the induction of the expression of TNF-α by compounds **2** and **5** having shorter secondary acyl chains exposed at the dimerization interface (at lower concentrations 0.4 – 100 ng/mL), whereas compounds **1** and **4** with longer lipid chains were less efficient in expressing TNF-α at lower concentrations (SI-Figure 3 C). The release of TNF-α induced by αα-GM-LAMs-*diP* was clearly more effective than the production of this cytokine by *Re*-LPS (Kdo_2-Lipid A).

The expression of the MyD88-dependent chemokine MCP-1 is associated with the activation of the intracellular TLR4·MD-2 complex.[5] Noteworthy, both the position of the phosphate group at the dimerization interface (at C-4 or C-6 of Man residue) and the length of the secondary acyl chain were responsible for the modulation of TLR4 stimulating activity which was monitored by measuring the levels of MCP-1 (SI-Figure 3 D). LAMs **2** and **5** with shorter secondary acyl chain (C_{10}) were generally more active than LAMs **1** and **4** having longer secondary acyl chain (C_{12}). As to phosphorylation pattern, the 4-phosphates **4** and **5** were more potent in induction of MCP-1 than the corresponding 6-phopshates **1** and **2** (compounds having lipid chains of equal length are compared).

Supplementary SI-Figure 4

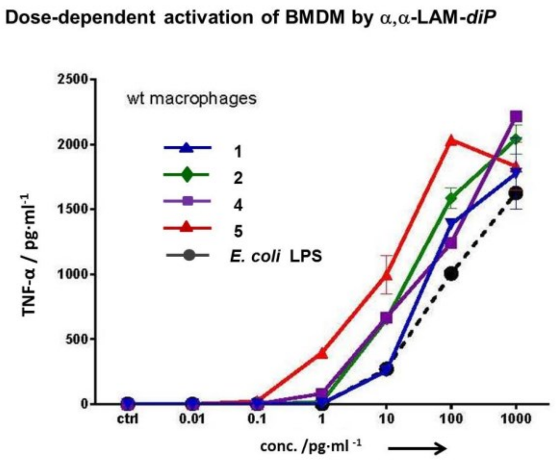

Bisphosphorylated LAMs **1, 2, 4** and **5** induced the release of TNF-α in mouse bone-marrow derived macrophages (BMDM) with high efficiency. Thus, αα-GM-LAMs are recognized and bound by both mouse and human TLR4 systems and effectively induce TLR4-mediated signaling also in mouse immune cells. Species-independent TLR4 modulation is one of the still not-yet-solved challenges in innate immunity and one of the major objectives of the present study.

SI-Figure 4. Dose-dependent induction of TNF-α induced by αα-GM-LAM-*diP* **1, 2, 4** and **5** in mouse BMDM

Supplementary SI-Figure 5

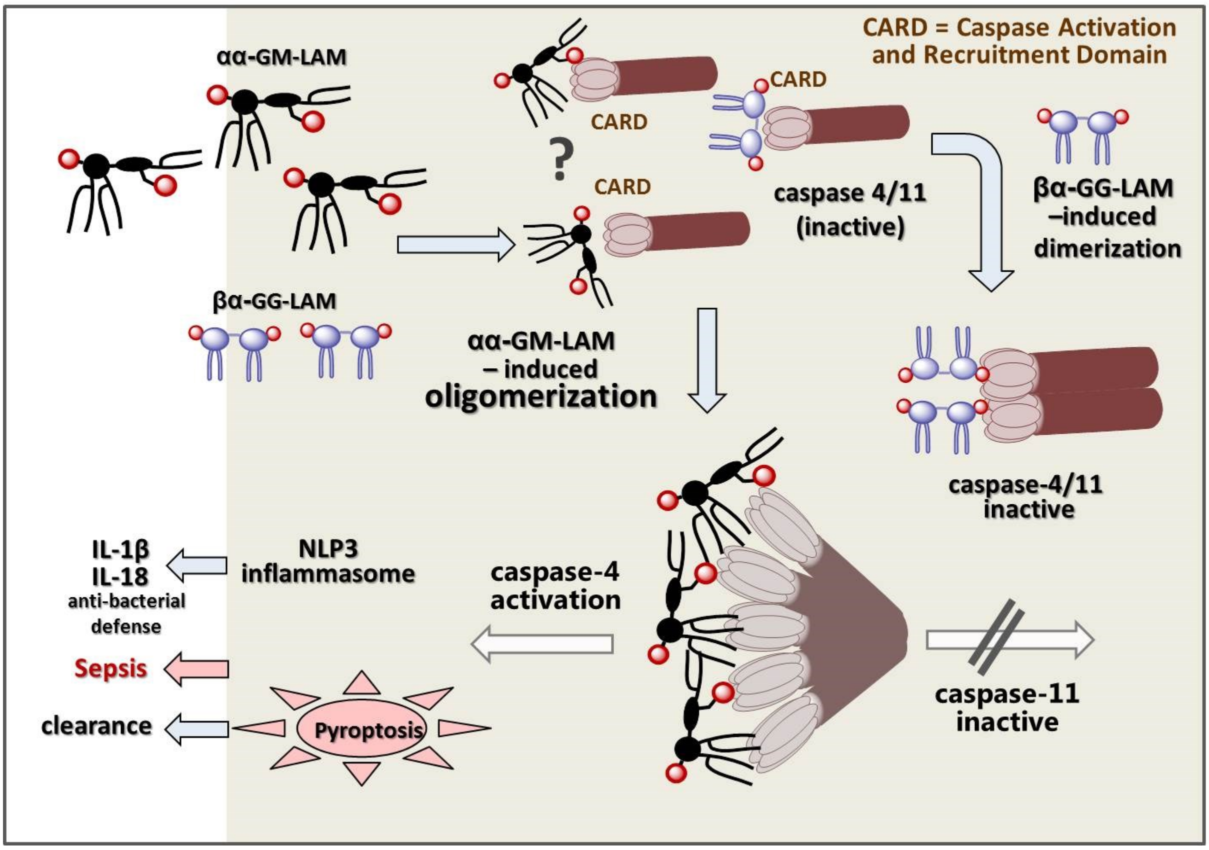

SI-Figure 5. Schematic representation of the interaction of αα-GM-LAM (TLR4 agonists) and ββ-GG-LAM (TLR4 antagonists) with inflammatory caspases. Image was generated with ChemDraw and PowerPoint.

Synthesis of immunostimulatory Lipid A mimetics (αα-GM-LAMs)

SI-Scheme 1.

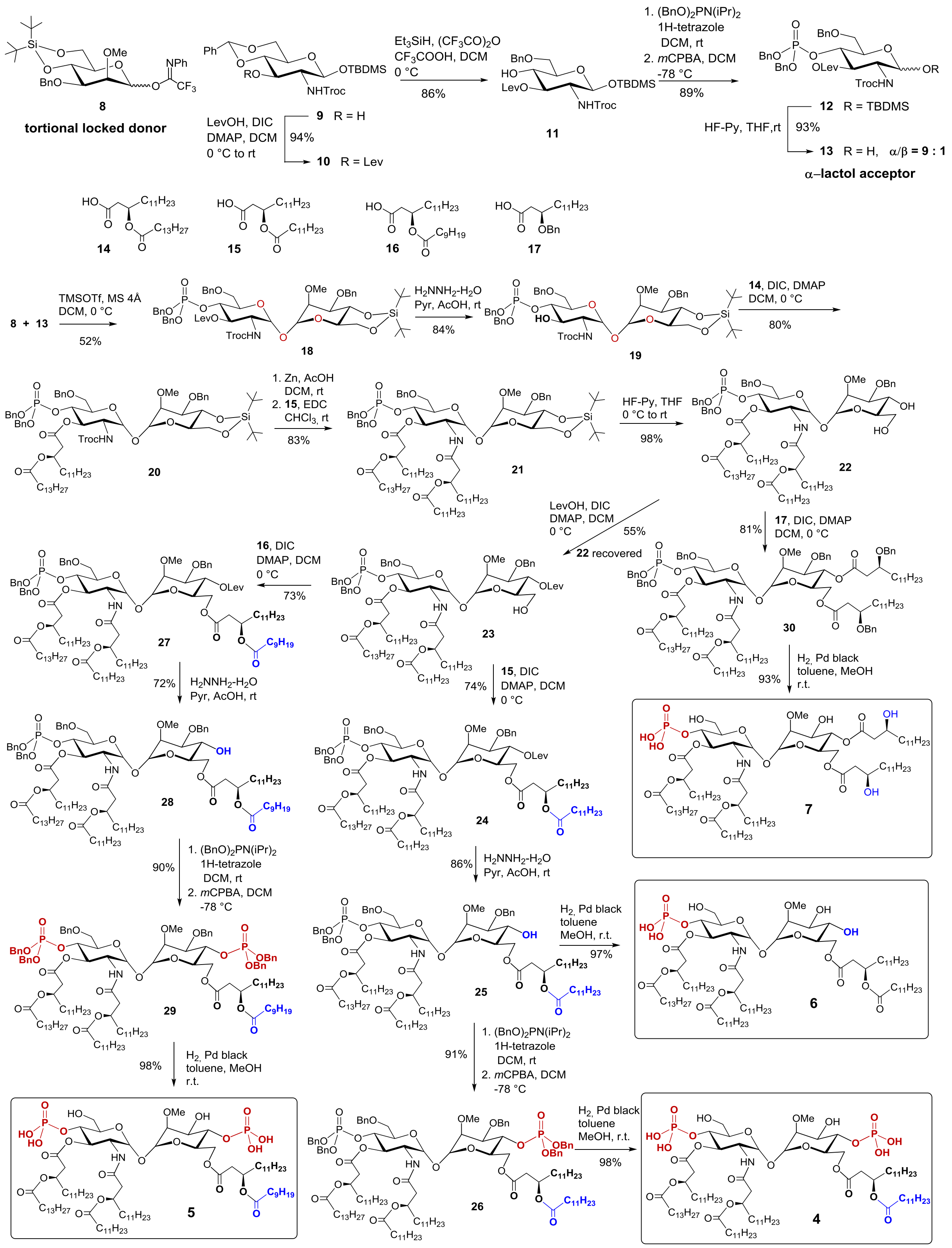

Scheme 1. Divergent synthesis of compounds **4, 5, 6** and **7**

Glycosylation reaction towards α(1↔1′)α-linked pyranosides

On the basis of a number of preliminary experiments we established special guidelines for the assembly of unsymmetric α(1↔1′)α-linked pyranosideses (for gluco- and manno- configuration):

1. Locking the glycosyl donor in $^4C_1$ conformation by application of a cyclic 4,6-*O*-protecting group is advantageous for unambiguous stereocontrol of 1-1′ glycosylation reaction. Application of "unlocked" 2-azido-protected GlcN-based donors or 2-*O*-Lev/2-*O*-Ac-protected Man-based donors resulted in a very poor stereoselectivity of glycosylation reaction (i.e. in addition to the desired α,α-1,1′-linked disaccharide, substantial amounts of the unseparable αβ-, βα- and ββ-counterparts were obtained).
2. Such conformational lock exerts a disarming effect on the glycosyl donor reactivity[6] which should be compensated by introduction of "arming" substituents/protecting groups.
3. For the α(1↔1′)α glycosylation involving Man-based donors:
   Electronically and stericaly disarmed 4,6-*O*-benzylidene acetal protected Man-derived donors are notorious for their extraodinary (1,2-*cis*) β-*manno*-stereoselectivity. Thus, to foster the α-selective glycosylation, the conformational lock could be attained by use of an alternative cyclic 4,6-di-*O*-*tert*-butylsilylene protecting group.
4. The oxocarbenium ion must be accessed by the α-lactol acceptor from the α-face in an S_N1 or S_N2 manner. However, the steric constrains around an arising α(1↔1′)α glycosidic linkage render the α-face less accesible which imposes specific requirements to the nature of the glycosyl donor. In our preliminary experiments, the *N*-phenylimidate donors provided best results in terms of stereoselectivity compared to thioethyl, thiophenyl and sulfoxide donors (the latter showed poorer stereoselectivity and provided much lower yields).
5. *N*-Phenylimidate Man-derived donors were found to be the most efficient and ensured better stereoselectivity and higher yields. Since the glycosylation reaction using *N*-Phenylimidate donors can be performed at the temperatures above 0° C, an application of "disarmed" and less nucleophilic lactol acceptors (which did not react at temperatures below 0° C) was possible. To match the reactivity of the glycosyl donor with the nucleophilicity of the α-lactol acceptor, the protection group pattern (at least seven orthogonal protecting groups) entailed in both donor and acceptor molecules had to be carefully adjusted. The "armed" glycosyl donors were combined with more reactive α-lactol acceptors and the relatively "disarmed" glycosyl donors were coupled to less nucleophilic α-lactol acceptors to result in the formation of the desired α,α-1,1′-linked glycosylation product.
6. Skewed molecular shape of the newly formed disaccharide (e.g. αGlcN(1↔1′)αMan) inflicts specific steric hindrance for the attack of the oxocarbenium ion from the α-face which results in a preferable formation of a β-linked product when tortional locked acceptors are applied. Conformational flexibility of the acceptor is important to relieve the conformational consrain and to facilitate an attack from the α-face. On this reason, a combination of the tortional locked Man-based donor with the tortional locked GlcN acceptor (e.g. acceptor entailing cyclic 4,6-*O*-protecting group) resulted in low yields of the α(1↔1′)α linked product. Application of a tortional unlocked GlcN acceptor is preferable.
7. The 2*N*-carbamate protected GlcN-hemiacetals revealed the highest α/β ratio (up to α/β = 9:1) of the anomeric 1-OH group, apparently, due to the presence of a carbamate N−H group capable of hydrogen bonding with the axial oxygen at C-1. Therefore, the 2*N*-Troc or 2*N*-Fmoc protected GlcN-hemiacetal acceptors are preffered.

Synthesis of αGlcN(1↔1′)αMan scaffold

The synthesis of the orthogonally protected αGlcN(1↔1)αMan scaffold relied on the application of the tortional locked 4,6-di-*O*-*tert*-butylsilylene protected Man-derived imidate donor **8** entailing both "arming" and moderately "disarming" protecting groups. Especially, a small protecting group for the the axial 2-OH (2-*O*-Me) was neccesary to eliminate sterical constrains in the arising αGlcN(1↔1′)αMan scaffold. Application of bulkier protecting groups (a participating *O*-Lev ester and a non-participating *O*-Bn group did not result in the formation of a disired α(1↔1′)α linkaged disaccharide).[7]

The GlcN-based 2-*N*-Troc protected lactol acceptors were previously applied by us for the synthesis of βα-(1↔1′)-linked disaccharides.[8] The presence of a carbamate NH able to form a hydrogen bond with the axial oxygen at C-1 allows for enhancement of the α/β-ratio in favor of the α-configured lactol. Interestingly, electron-donating groups at position O-3 (e.g. TBDMS) shifted the α/β-ratio in the direction of β-configured hemiacetal whereas electron-withdrawing substituents (e.g. Ac or Lev protecting groups) favored the α-configuration of the GlcN-based hemiacetal acceptor.

The synthesis of the GlcN based acceptor **13** was performed in 10 steps starting from glucosamine hydrochloride (Scheme 1). The 2-*N*-Troc protected 4,6-*O*-benzylidene acetal **8** was reacted with levulinic acid using DIC and cat. DMAP as activating reagent which furnished 3-*O*-Lev protected **10**. Regioselective reductive opening of benzylidene acetal in **10** was performed with $Et_3SiH/(CF_3CO)_2O$ in the presence of CF_3COOH to give **11.** Phosphorylation of the liberated 4-OH group using phosphoramidite procedure provided **12**. Finally, removal of the anomeric TBDMS group by pyridinium hydrofluoride (HF•Py) afforded orthogonally protected glucosamine hemiacetal acceptor **13** (α/β = 9 : 1).

A TMSOTf - promoted glycosylation of **13** by *N*-phenyl trifluoroacetimidate mannose-based donor **8** in DCM at 0 °C furnished, after careful optimization of reaction conditions (solvent, temperature, sequence of addition, excess of the donor), an αGlcN(1↔1′)αMan disaccharide **14** in 52% yield. The major by-product (15%) constituted an αGlcN(1↔1′)βMan counterpart. Appropriate combination of protecting groups in the newly formed α(1↔1′)α disaccharide **14** allowed for uncomplicated isolation by column chronmatography on silica gel and easy separation of the by-products. The yields for the stereoselective α(1↔1)α glycosylation (e.g., for the synthesis of α,α-trehalose and derivatives) reported in the literature vary from 20 to 60% and refer to simply and mainly symmetrically protected glucoses (peracetylated or perbenzylated).[9] Improved yields for the α(1↔1′)α glycosylation using non-symetric protecting group pattern were reported for intramolecular aglicon delivery technique (IAD).[10,11] However, the isolation of pure disaccharide product following IAD glycosylation was inefficient and the yields (~ 60%) were reported for a crude reaction mixture, whereas the isolation of target α,α-1,1′-linked disaccharide in pure form was possible only after peracetylation which gave ~ 40% overall yield. Since IAD technique would be rather complicated and less efficient for the α(1↔1)α glycosylation involving aminosugar, we did not select this approach. The highest yields for the synthesis of nonsymetrical 1,1′-disacchrides were achived by application of STol-glycoside donors (Glc-, Gal-, GlcN- and GalN-based donors) and picolyl-protected TMS-α-glycoside acceptors.[12] Still, this approach required the presence of at least four-five benzyl groups in the arising 1,1′-disaccharide which was not compatible with the synthesis of our target structures. We are quite confident that the obtained by us 52% isolated yield of fully orthogonally protected αGlcN(1↔1′)αMan scaffold could be assessed as good or even excellent.

Synthesis of Lipid A mimetics (LAMs) based on the αGlcN(1↔1′)αMan scaffold.

The deprotection of 3-*O*-levulinoyl group in **18** was performed by treatment with hydrazine hydrate buffered with pyridine and acetic acid to give **19**, which was subsequently acylated at *O*-3 by reaction with (*R*)-3-(tetradecanoyloxy)tetradecanoic acid **14** in the presence of DIC and DMAP. Gradual addition of a diluted solutions of the long-chain fatty acid **14** and DIC and reduced temperature (0 °C) was crucial for not obtaining the concomitant co-migrating 3-*O*-tetradecanoyl- and 3-*O*-alkenoyl- by-products**.**[7] Application of higher amounts of DIC and/or fatty acid with the aim to accelerate the reaction resulted in an augmented formation of 3-*O*-alkenoyl by-product, which was rationalized by β-elimination or rearrangement[13] of the *in situ* formed *O*-acyl-oxyacylisourea intermediate. The formation of 3-*O*-alkanoyl by-products in the DIC/DMAP–mediated acylation was previously reported, although the elimination towards 3-*O*-alkenoyl- by-products was not previously detected.[2] The reductive cleavage of the *N*-Troc group in **20** with Zn in acetic acid gave an intermediate amine which was subjected to acylation with (*R*)-3-(dodecanoyloxy)tetradecanoic acid **15** under the action of EDC to result in fully protected tetraacylated disaccharide **21**. Next, the HF•Py mediated cleavage of the 4,6-di-*O*-*tert*-butylsilylene (DTBS) group furnished the key diol **22**.

Due to a staggered gauche-gauche conformation of αGlcN(1↔1′)αMan disaccharide (specific 3D-arrangement with respect to the dihedrals about α,α-(1↔1′) glycosidic linkage resulting in a skewed relative orientation of two Glc rings for variably substituted α,α-trehaloses is proved by crystal structures and molecular dynamic simulation studies [14-21]) the primary OH group at position 6 in diol **22** is sterically hindered. Additionally, 6-OH group is electronically disarmed due to the intra-molecular hydrogen bonding with 2′-*N*H and C=O of the acyl chain attached at the GlcN moiety. Thus, it should be possible to regioselectively protect the secondary 4-OH group in **22** leaving the primary 6-OH group unaffected.

Our initial attempts to perform the regioselective phosphorylation of the 4-OH group in **22** using phosphoramidite approach resulted in a double phosphorylation at position 4- and 6 independently of the structure of the phosphoramidite reagent used. Notwithstanding, the 4- and 6- OH groups in diol **22** could be differentiated using acylation reaction under Steglich conditions. Thus, the secondary hydroxyl group at position 4 in the diol **22** could be regioselectively temporarily protected as levulinoyl ester to give **23**. The primary 6-OH group in **23** was subsequently acylated with either (*R*)-3-(dodecanoyloxy)tetradecanoic acid **15** in the presence of DIC and DMAP to furnish **24** or with (*R*)-3-(decanoyloxy)tetradecanoic acid **16** under similar conditions to provide hexaacylated compound **27**. The levulinoyl group at C-4 was subsequently cleaved with hydrazine hydrate in pyridine – acetic acid to furnish **25** and **28**, respectively. The liberated 4-OH group was phosphorylated by reaction with dibenzyl *N,N*-diisopropylphosphoramidite in the presence of 1*H*-tetrazole followed by oxidation of the intermediate phosphite triester with *m*CPBA at -78 °C to give fully protected diphosphates **26** and **29**, respectively.

Global deprotection of the hexaacylated diphosphates **26** and **29**, as well as hexaacylated monophosphates **25** and **30** by hydrogenation over Pd black in toluene-methanol furnished target LAMs **4, 5, 6** and **7**. For biological studies, the final compounds were purified by size-exclusion chromatography on BioBeads SX1 (BioRad) using chloroform – toluene – methanol mixtures as eluent. Compounds **1 - 3** were prepared as previously reported by us.[7]

Experimental procedures

Experimental Procedures: Biological assays

Solubilization of synthetic compounds, preparation of test samples.

Compounds **1 – 7** (3.0 mg) were dissolved in DMSO (TLR4 grade, 6 mL) to provide 0.5 mg/mL solutions in DMSO which were subsequently aliquoted (0.1 mg, 200 µL of a solution in DMSO) in 1 mL glass vials and lyophilized. The content of the vial (0.1 mg of compounds **1 – 7**) was reconstituted in DMSO (100 µL) under vortex (10 min). Aliquots (50 µL) were placed in 1 mL glass vials and diluted with 450 µL of cell medium (RPMI or DMEM) supplemented with 10% FCS (fetal calf serum) under vortex (10 min) to provide 500 µL of a 100 µg/mL stock solution (Stock I). Stock I was used for further dilutions with aqueous buffers or cell medium (as indicated below for each particular experiment) to provide aqueous solutions of compounds **1 -7** which were used for dose-dependent cell activation assays. Glass vials were used for all dilutions. The final concentration of DMSO in the cell cultures did not exceed 0.01%. Stability and integrity of compounds **1 – 7** in aqueous buffers (100 µg/mL) at the temperatures 25 – 37 °C at 12h, 36 h and 72h incubation time was confirmed using MALDI-TOF spectroscopy.

Stimulation of transient hTLR4/MD-2/CD14 transfected HEK293 cells.

HEK293 cells were transfected for 24 h with plasmids coding for human TLR4, human MD-2 (kind gift of K. Miyake, Tokyo, Japan), and human CD14 (kind gift of D. Golenbock, Worcester, USA), using Lipofectamin2000 (Invitrogen GmbH, Karlsruhe, Germany) according to the manufacture's instruction. Solutions of compounds **1 – 7** were prepared as described above using DMEM cell medium supplemented with 10% FCS. Next, transiently transfected cells were stimulated with increasing concentrations of compounds **1 – 7** or *E. coli* O111:B4 LPS for αα-GM-LAM-*diP* **1, 2, 4**, and **5** and *S. minnesota* R595 MPLA (Invivogen) for αα-GM-LAM-*monoP* **3, 6, 7** for 20h. Recombinant human TNF-α (kind gift of D. Männel, Regensburg, Germany) served as transfection-independent control. IL-8 production was measured by human IL-8 CytoSet ELISA (Invitrogen GmbH, Karlsruhe, Germany) according to the manufacture's instruction. Data were combined from n=2 independent experiments, error bars indicate standard deviation of the mean.

Assay in human mononuclear cells (MNC).

Peripheral blood mononuclear cells (MNC) from healthy human volunteers (prepared from heparinized blood by gradient centrifugation using Biocoll, Merck, Darmstadt, Germany) were incubated at a concentration of 1 x 10^6/mL in 96-well tissue culture plates at a volume of 150 µL using RPMI-1640 medium supplemented with 100 U/mL penicillin, 100 µg/mL streptomycin (both PAA Laboratories GmbH), and 10% of heat-inactivated FCS (Merck Millipore, Biochrom AG, Berlin, Germany). Solutions of compounds **1 – 7** were prepared as described above using PRMI cell medium supplemented with 10% FCS. Cells were then stimulated with increasing concentrations of compounds **1 – 7** or *E. coli* O111:B4 LPS for αα-GM-LAM-*diP* **1, 2, 4,** and **5** and MPLA for αα-GM-LAM-*monoP* **3, 6, 7**. After a culture period of 20 hours at 37 °C, culture supernatants were harvested and the IL-6 and TNF-α content was determined using an ELISA according to the manufacturers' protocol (Invitrogen GmbH, Karlsruhe, Germany). Data shown are exemplary for n=3 independent experiments, error bars indicate standard deviation of the mean.

Assay in human bronchial epithelial cells.

Calu-3 cells (a human lung epithelial cell line; ATCC) were seeded in 96 well plates at 10E5 cells/well in 100 µl of complete medium (RPMI1640 (PAA), 1% PS (PAA), 10% FCS (Biochrom, Berlin, Germany). On the next day, cells were washed once with complete medium and stimulated with increasing concentrations of compounds **1 – 7** or *E. coli* O111:B4 LPS for αα-GM-LAM-*diP* **1, 2, 4,** and **5** and MPLA for αα-GM-LAM-*monoP* **3, 6, 7**. The total volume of the well after stimulation reached 200µl. The cells were incubated for 20-24 h and the supernatants were analyzed for IL-8 and IL-6 by ELISA (Invitrogen GmbH, Karlsruhe, Germany). Data were combined from n=2 independent experiments, error bars indicate standard error of the mean.

Differentiation and stimulation of THP-1 cells.

The THP-1 human monocyte-like cell line was obtained from Dr. Rene Devos (Roche Research Ghent) and originally purchased from ATCC. The phorbol ester 12-*O*-tetradecanoylphorbol-13-acetate (TPA) was purchased from Sigma. THP-1 cells were grown in RPMI-1640 cell-culture medium (Life Technologies) that was supplemented with 2 mM L-glutamine, 100 U/mL penicillin, 100 µg/mL streptomycin, and 10% FCS. Cells were seeded in a 96 well plate at 10E5 cells/well in 150 µl complete medium and stimulated by 200 nM TPA for 24 h to induce the differentiation into macrophage-like cells.[22] On the next day the cells were washed twice with complete culture medium to discard the cells that did not adhere, refreshed with 200 µl complete medium and left for 1h to recover. Solutions of compounds **1 – 7** were prepared as described above using RPMI-1640-cell-culture medium supplemented with 10% FCS. Cells were stimulated with αα-GM-LAM-*diP* **1, 2, 4,** and **5** at the indicated concentration and with *E. coli* Re-LPS (or *E. coli* O111:B4 LPS, both purchased from InvivoGen), which were added as solutions in 10 µl complete medium. The total volume of the well after stimulation reached 220 µl. The cells were incubated for 18 h and the supernatants were analyzed for IL-1β, TNF-α and MCP-1 by ELISA (BD Biosciences). Data are the mean of two samples (n=2) and are representative for two independent experiments. Error bars indicate standard deviation.

Stimulation of bone marrow-derived mouse macrophages (BMDM).

Immortalized C57BL/6 wt mouse macrophage cell line was kindly provided by D.T. Golenbock[23] (Worcester, MA, USA) and propagated in RPMI medium (PAA, Linz, Austria) containing 10% FCS, 20 mM HEPES buffer, 2 mM L-Glutamin (both PAA, Linz, Austria) and 20 µg/ml gentamicin (Sigma, Deisenhofen, Germany). Subsequently, cells were stimulated with increasing concentrations of compounds **1-7** or *E. coli* O111:B4 LPS for 20h. TNF-α production was measured by human TNF-α CytoSet ELISA (Invitrogen GmbH, Karlsruhe, Germany) according to the manufacture's instruction. Data shown are exemplary for n=2 independent experiments, error bars indicate standard deviation of the mean.

Caspase-4/11 oligomerization analyses by pore-limit native gel.

Pore-limit native gel electrophoresis was used to analyze the ability of αα-GM-LAMs **1 -7** to induce caspase-4/11 (catalytic inactive, purified from insect cells) oligomerization. The experiments were performed according to procedures described by us previously using *E coli* Lipid A and *E coli* LPS as standards.[24] Briefly, 4% - 20% polyacrylamide gels were prepared and run in a buffer containing 50 mM Tris-HCl (pH 9.0), 7 mM EDTA and 2 mM boric acid at a voltage of 80-100 V for 6-8 h. Recirculation system was used between the two buffer reservoirs.

Caspase-4/11 activity measurement.

The protease activity of caspase-4/11 was measured in the assay buffer (50 mM HEPES (pH 7.5), 150 mM NaCl, 3 mM EDTA and 0.005% (v/v) Tween-20 and 10 mM DTT) as described by us earlier.[24] Briefly, each ligand (αα-GM-LAMs **1 -7**, *E.coli* Lipid A or *E.coli* LPS) was incubated with catalytically competent caspase-4/11 (0.3 µM caspase-11 or 0.1 µM caspase-4, purified from insect cells) in a 100-µL reaction at 30°C for 30 min. After the incubation, a generic substrate zVAD-AMC was added to a final concentration of 75 µM and incubated at 37°C for 30 min. Hydrolysis of zVAD-AMC (generating fluorogenic AMC) by recombinant caspase proteins was measured by monitoring the emission at 450 nm after excitation at 365 nm on a fluorescent reader (PerkinElmer EnSpire™ Multimode Plate Reader). Fluorescence signal of the mock reaction containing only the substrate was treated as the background signal.

Experimental Procedures: Synthesis

General synthetic methods

Reagents and solvents were purchased from commercial suppliers and used without further purification unless otherwise stated. Dichloromethane was distilled from CaH_2 and stored over activated 4 Å molecular sieves (MS). THF was distilled over Na/benzophenone directly before use. Toluene was distilled successively over P_2O_5 and CaH_2 and stored over activated 4 Å MS. Other solvents were dried by storage over activated MS for at least 48 h prior to use [acetonitrile (3 Å) and DMF (3 Å)]. Residual moisture was determined by colorimetric titration on a Mitsubishi CA-21 Karl Fischer apparatus and did not exceed 20 ppm for dry solvents. Reactions were monitored by TLC performed on silica gel 60 F254 HPTLC pre-coated glass plates with a 25 mm concentration zone (Merck). Spots were visualized by UV light followed by dipping into a H_2SO_4-*p*-anisaldehyde solution or a ninhydrin-EtOH solution and subsequent charring at 250°C. Solvents were removed under reduced pressure at 30°C. HPLC was performed with linear solvent gradients on a YMC Pack SIL-06 250×20mm, S-5 µm, 6nm column (Column A, loadings 50 mg – 150 mg), or on a YMC Pack SIL-06 250×10mm, S-5 µm, 6nm column (Column B, loadings 5 mg – 50 mg). Preparative MPLC was performed on silica gel 60 (230–400 mesh, Merck). Size exclusion chromatography was performed on BioRad Sephadex LH-20 or Bio-Beads SX1 supports. NMR spectra were recorded at 298 K on a Bruker Avance III 600 spectrometer (^{1}H at 600.22 MHz; ^{13}C at 150.92 MHz; ^{31}P at 242.97 MHz) or on Bruker DPX 400 spectrometer (^{1}H at 400.13 MHz; ^{13}C at 100.61 MHz; ^{31}P at 161.68 MHz) or on Bruker AC 300 spectrometer (^{1}H at 300.13 MHz; ^{13}C at 75.49 MHz; ^{31}P at 121.49 MHz) using standard Bruker NMR software. Chemical shifts are reported in ppm, ^{1}H-NMR spectra in $CDCl_3$ are referenced to internal TMS, ^{13}C-spectra are referenced to the corresponding solvent signal (77.00 ppm for $CDCl_3$). NMR spectra in other solvents are referenced to residual solvent signals (for MeOD: 3.31 ppm, 49.00 ppm, ^{1}H- and ^{13}C-NMR, respectively). ^{31}P-Spectra in $CDCl_3$ are referenced according to IUPAC recommendations 2001 from a referenced ^{1}H-spectrum. In the disaccharides the mannose NMR signals are indicated by primes. High resolution mass spectrometry (HRMS) was carried out from 1–10 $mg \cdot l^{-1}$ acetonitrile solutions via LC-TOF MS (Agilent 1200SL HPLC and Agilent 6210 ESI–TOF, Agilent Technologies). The mass spectrometer was tuned with Agilent tune mix to provide a mass accuracy below 2 ppm. The data were analyzed using Agilent Mass Hunter Software. MALDI-TOF was performed in the negative mode using a Bruker Autoflex Speed TOF-TOF instrument with 6-aza-2-thiothymine (ATT) as matrix. Spectra were processed with the manufacturer's software (Bruker Flexanalysis 3.3.80) using the SNAP algorithm with a signal/noise threshold of 6 (unsmoothed). Optical rotation was measured on a Perkin Elmer 243 B polarimeter, equipped with a Haake water circulation bath and a Haake D1 immersion circulator for temperature control of the measuring cell. $[\alpha]_D^{20}$ Values are given in units of deg $dm^{-1}cm^3g^{-1}$.

Synthesis of 10

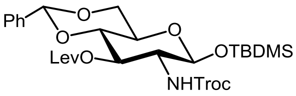

***tert*-Butyldimethylsilyl 4,6-*O*-benzylidene-2-deoxy-3-*O*-levulinoyl-2-(2,2,2-trichloroethoxy-carbonylamino)-β-D-glucopyranoside (10).**

To a stirred solution of **9** (3.48 g, 6.25 mmol), DMAP (38 mg, 0.31 mmol) and levulinic acid (1.4 mL, 13.6 mmol) in dry DCM (20 mL), DIC (1.1 mL, 7.1 mmol) was added at 0 °C. After 5 min the mixture was allowed to warm to r.t. and stirred for 5.5 h. The solids were removed by filtration over Celite and the filtrate was concentrated. The residue was purified by column chromatography on silica gel (hexane – EtOAc, 3 : 1) to afford **10** (3.87 g, 5.91 mmol, 95%) as white amorphous solid. R_f = 0.61 (toluene : EtOAc, 4:1); $[\alpha]_D^{20}$ = -31 (*c* 1.1, $CHCl_3$);

^{1}H-NMR (300 MHz, $CDCl_3$): δ 7.47-7.41 (m, 2H, Ph), 7.38-7.33 (m, 3H, Ph), 5.50 (s, 1H, C*H*Ph), 5.32 (t, 1H, $^3J_{3,4}$ = $^3J_{3,2}$ = 10.0 Hz, H-3), 5.21 (d, 1H, $^2J_{NH,2}$ = 9.3 Hz, N*H*), 4.86 (d, 1H, $^3J_{1,2}$ = 7.9 Hz, H-1), 4.73 (s, 2H, C*H*$_2$, Troc), 4.30 (dd, 1H, $^3J_{6a,5}$ = 5.0 Hz, $^2J_{6a,6b}$ = 10.5 Hz, H-6a), 3.80 (t, 1H, $^3J_{6b,5}$ = $^2J_{6b,6a}$ = 10.3 Hz, H-6b), 3.71 (t, 1H, $^3J_{4,5}$ = $^3J_{5,4}$ = 9.4 Hz, H-4), 3.68-3.59 (m, 1H, H-2), 3.50 (dt, 1H, $^3J_{5,6a}$ = 4.9 Hz, $^3J_{5,6b}$ = $^3J_{5,4}$ = 9.7 Hz, H-5), 2.83-2.49 (m, 4H, 2×C*H*$_2$), 2.13 (s, 3H, C*H*$_3$, Lev), 0.88 (s, 9H, 3×C*H*$_3$, TBDMS), 0.11 (s, 3H, C*H*$_3$, TBDMS), 0.09 (s, 3H, C*H*$_3$, TBDMS).

^{13}C-NMR (75 MHz, $CDCl_3$): δ 206.03 (*C*O, Lev), 172.69 (*C*O, Lev), 154.19 (*C*O, Troc), 136.96 (*C*q, Ph), 129.04, 128.18, 126.17 (5×*C*H, Ph), 101.37 (*C*HPh), 96.78 (C-1), 95.40 (CCl_3, Troc), 78.78 (C-4), 74.72 (*C*H$_2$, Troc), 71.38 (C-3), 68.58 (C-6), 66.38 (C-5), 59.01 (C-2), 37.97 (*C*H$_2$, Lev), 29.69 (*C*H$_3$, Lev), 27.99 (*C*H$_2$, Lev), 25.50 (3×*C*H$_3$, TBDMS), 17.83 (*C*q, TBDMS), -4.24, -5.33 (2×*C*H$_3$, TBDMS).

HRMS (+ESI) m/z: calcd for $C_{27}H_{42}Cl_3N_2O_9Si$ $[M+NH_4]^+$ 671.1720, found 671.1726.

Synthesis of 11

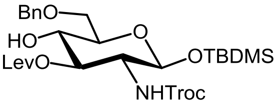

***tert*-Butyldimethylsilyl 6-*O*-benzyl-2-deoxy-3-*O*-levulinoyl-2-(2,2,2-trichloroethoxycarbonylamino)-β-D-glucopyranoside (11).**

To a solution of **10** (2.95 g, 4.50 mmol), trifluoroacetic anhydride (1.9 mL, 13.5 mmol) and triethylsilane (1.6 mL, 23.2 mmol) in dry DCM (44 mL) trifluoroacetic acid (1.8 mL, 23.5 mmol) was added at 0 °C. The mixture was stirred for 8 h at 0 °C. Aq. sat. $NaHCO_3$ (50 mL) was added and the mixture was warmed to r.t. The mixture was diluted with DCM (150 mL) and the organic layer was washed with water (100 mL) and brine (100 mL). The organic phase was dried over Na_2SO_4, filtered and concentrated. The residue was purified by column chromatography on silica gel (toluene – EtOAc, 3:1) to afford **11** (2.56 g, 3.90 mmol, 86%) as colorless syrup. R_f = 0.24 (toluene – EtOAc, 2:1); $[\alpha]_D^{20}$ = -20 (*c* 1.0, $CHCl_3$).

^{1}H-NMR (600 MHz, $CDCl_3$): δ 7.36-7.32 (m, 4H, CH_2Ph), 7.31-7.27 (m, 1H, CH_2Ph), 5.14 (d, 1H, $^3J_{NH,2}$ = 9.4 Hz, N*H*), 5.04 (dd, 1H, $^3J_{3,4}$ = 9.3 Hz, $^3J_{3,2}$ = 11.0 Hz, H-3), 4.73-4.71 (m, 1H, C*H*$_2$, Troc), 4.73 (d, 1H, $^3J_{1,2}$ = 8.2 Hz, H-1), 4.67 (AB, ^2J = 11.9 Hz, Troc), 4.61 (AB, 1H, ^2J = 12.1 Hz, C*H*$_2$Ph), 4.58 (AB, 1H, ^2J = 12.1 Hz, C*H*$_2$Ph), 3.80-3.72 (m, 3H, H-4, H-6a, H-6b), 3.63-3.58 (m, 1H, H-2), 3.56-3.53 (m, 1H, H-5), 3.27 (br. s, 1H, 4-O*H*), 2.83-2.74 (m, 2H, C*H*$_2$, Lev), 2.61-2.51 (m, 2H, C*H*$_2$, Lev), 2.16 (s, 3H, C*H*$_3$, Lev), 0.88 (s, 9H, 3×C*H*$_3$, TBDMS), 0.13 (s, 3H, C*H*$_3$, TBDMS), 0.09 (s, 3H, C*H*$_3$, TBDMS);

^{13}C-NMR (151 MHz, $CDCl_3$): δ 207.31 (*C*O, Lev), 173.35 (*C*O, Lev), 154.19 (*C*O, Troc), 137.90 (*C*q, C*H*$_2$Ph), 128.40, 127.70, 127.59 (5×*C*H, CH_2Ph), 96.42 (C-1), 95.45 (*C*Cl$_3$), 75.52 (C-3), 74.63 (*C*H$_2$, Troc), 74.37 (C-5), 73.67 (*C*H$_2$Ph), 70.51 (C-6), 70.11 (C-4), 57.81 (C-2), 38.32 (*C*H$_2$, Lev), 29.72 (*C*H$_3$, Lev), 28.22 (*C*H$_2$, Lev),

25.55 (3×CH_3, TBDMS), 17.88 (*C*q, TBDMS), -4.14, -5.31 (2×CH_3, TBDMS);

HRMS (+ESI) m/z: calcd for $C_{27}H_{44}Cl_3N_2O_9Si$ $[M+NH_4]^+$ 673.1876, found 673.1881.

Synthesis of 12

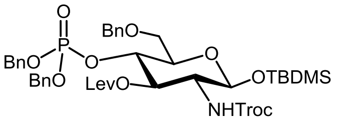

***tert*-Butyldimethylsilyl 6-*O*-benzyl-4-*O*-[bis(benzyloxy)phosphoryl]-2-deoxy-3-*O*-levulinoyl-2-(2,2,2-trichloroethoxycarbonylamino)-β-D-glucopyranoside (12).**

To a stirred solution of **11** (2.56 g, 3.90 mmol) in dry DCM (40 mL) dibenzyl *N,N*-diisopropylphosphoramidite (2.0 mL, 5.37 mmol, 90%) and 1*H*-tetrazole (13 mL, 5.85 mmol, 0.45 M in CH_3CN) were added at r.t. After 2 h the mixture was cooled to -78 °C and a solution of *m*-CPBA (1.5 g, 6.08 mmol) in DCM (40 mL) was added. After stirring for 1 h, the reaction was quenched by addition of Et_3N (1.3 mL) and the mixture was warmed to r.t. The mixture was diluted with EtOAc (150 mL) and washed with aq. citric acid (0.25 M, 50 mL), aq. sat.$NaHCO_3$ (2x50 mL), water (2x50 mL) and brine (50 mL). The organic layer was dried over Na_2SO_4, filtered and concentrated. The residue was purified by column chromatography on silica gel (toluene – EtOAc, 10:1) to afford **12** (3.18 g, 3.47 mmol, 89%) as a white solid. R_f = 0.40 (toluene – EtOAc, 4:1); $[\alpha]_D^{20}$ = -7.0 (*c* 1.0, $CHCl_3$);

^{1}H-NMR (600 MHz, $CDCl_3$): δ 7.35-7.23 (m, 15H, CH_2Ph), 5.34 (dd, 1H, J = 9.2 Hz, J = 10.6 Hz, H-3), 5.11 (d, 1H. $^3J_{NH,2}$ = 8.8 Hz, N*H*), 4.95-4.90 (m, 4H, OP(O)(OCH_2Ph)$_2$), 4.87 (d, 1H, $^3J_{1,2}$ = 7.9 Hz, H-1), 4.75 (AB, 1H, ^2J = 12.0 Hz, CH_2, Troc), 4.67 (AB, 1H, ^2J = 12.0 Hz, CH_2, Troc), 4.52 (AB, 1H, ^2J = 11.9 Hz, CH_2Ph), 4.51-4.46 (m, 1H , H-4), 4.47 (AB, 1H, ^2J = 11.9 Hz, CH_2Ph), 3.77-3.74 (m, 1H, H-6a), 3.65-3.61 (m, 2H, H-5, H-6b), 3.51-3.46 (m, 1H, H-2), 2.61-2.56 (m, 1H, CH_2, Lev), 2.52-2.44 (m, 2H, CH_2, Lev), 2.40-2.35 (m, 1H, CH_2, Lev), 2.08 (s, 3H, CH_3, Lev), 0.88 (s, 9H, 3×CH_3, TBDMS), 0.13 (s, 3H, CH_3, TBDMS), 0.09 (s, 3H, CH_3, TBDMS);

^{13}C-NMR (151 MHz, $CDCl_3$): δ 206.21 (*C*O, Lev), 172.64 (*C*O, Lev), 153.98 (*C*O, Troc), 138.14 (*C*q, CH_2Ph), 135.63, 135.58 (2×*C*q, OP(O)(OCH_2Ph)$_2$), 128.59, 128.57, 128.28, 127.99, 127.93, 127.49, 127.47 (15×*C*H, CH_2Ph), 95.76 (C-1), 74.66 (*C*H_2, Troc), 74.21 (C-4, $J_{C,P}$ = 6.0 Hz), 74.05 (C-5, $J_{C,P}$ = 6.2 Hz), 73.39 (*C*H_2Ph), 72.50 (C-3), 69.61, 69.57, 69.53 (2×OP(O)(O*C*H_2Ph)$_2$), 68.63 (C-6), 58.54 (C-2), 37.79 (*C*H_2, Lev), 29.65 (*C*H_3, Lev), 27.96 (*C*H_2, Lev), 25.55 (3×*C*H_3, TBDMS), 17.89 (*C*q, TBDMS), -4.21, -5.32 (2×*C*H_3, TBDMS);

^{31}P-NMR (243 MHz, $CDCl_3$): δ -2.28;

HRMS (+ESI) m/z: calcd for $C_{41}H_{53}Cl_3NNaO_{12}PSi$ $[M+Na]^+$ 938.2032, found 938.2027.

Synthesis of 13

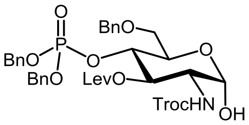

6-*O*-Benzyl-4-*O*-[bis(benzyloxy)phosphoryl]-2-deoxy-3-*O*-levulinoyl-2-(2,2,2 trichloroethoxycarbonyl-amino)-α-D-glucopyranose (13).

To a solution of compound **13** (1.40 g, 1.53 mmol) in dry THF (14 mL) placed in a PTFE-vial a solution of HF•pyridine (800 µL) was added at 0 °C. After 10 min the mixture was warmed to r.t. and stirred for 15 h. The reaction mixture was diluted with EtOAc (150 mL) and washed with aq. sat. $NaHCO_3$ (150 mL) and brine (150 mL). The organic layer was dried over Na_2SO_4, filtered and concentrated. The residue was purified by column chromatography on silica gel (toluene – EtOAc, 3:2) to afford compound **13** (1.14 g, 1.42 mmol, 93%, α:β = 10:1) as white amorphous solid. R_f = 0.48 and 0.28 (toluene – EtOAc, 1:1); $[\alpha]_D^{20}$ = +15 (*c* 1.0, $CHCl_3$);

^{1}H-NMR (300 MHz, $CDCl_3$, α-anomer): δ 7.34-7.23 (m, 15H, CH_2Ph), 5.38 (dd, 1H, $^{3}J_{3,4}$ = 9.2 Hz, $^{3}J_{3,4}$ = 10.7 Hz, H-3), 5.30 (d, 1H, $^{3}J_{NH,2}$ = 10.1 Hz, N*H*), 5.26 (d, 1H, $^{3}J_{1,2}$ = 3.2 Hz, H-1), 4.93-4.88 (m, 4H, OP(O)(OCH_2Ph)$_2$), 4.80 (AB, 1H, ^{2}J = 12.0 Hz, CH_2, Troc), 4.71 (AB, 1H, ^{2}J = 12.0 Hz, CH_2, Troc), 4.52 (AB, 1H, ^{2}J = 12.1 Hz, CH_2Ph), 4.49-4.41 (m, 1H, H-4), 4.43 (AB, 1H, ^{2}J = 12.1 Hz, CH_2Ph), 4.19-4.13 (m, 1H, H-5), 3.97 (td, 1H, $^{3}J_{2,1}$ = 3.5 Hz, $^{3}J_{2,3}$ = $^{3}J_{2,NH}$ = 10.2 Hz, H-2), 3.73 (dd, 1H, $^{3}J_{6a,5}$ = 2.0 Hz, $^{2}J_{6a,6b}$ = 10.9 Hz, H-6a), 3.62 (dd, 1H, $^{3}J_{6b,5}$ = 5.8 Hz, $^{2}J_{6b,6a}$ = 10.8 Hz, H-6b), 3.48 (br. s, 1H, 1-O*H*), 2.63-2.32 (m, 4H, 2×CH_2, Lev), 2.07 (s, 3H, CH_3, Lev);

^{13}C-NMR (75 MHz, $CDCl_3$, α-anomer): δ 206.10 (*C*O, Lev), 172.89 (*C*O, Lev), 154.18 (*C*O, Troc), 137.58 (*C*q, *C*H$_2$Ph), 135.53 (*C*q, $^{3}J_{C,P}$ = 2.8 Hz, OP(O)(OCH$_2$Ph)$_2$), 135.43 (*C*q, $^{3}J_{C,P}$ = 3.2 Hz, OP(O)(OCH$_2$Ph)$_2$), 128.64, 128.62, 128.60, 128.58, 128.38, 128.04, 127.98, 127.86, 127.79 (15×*C*H, CH$_2$Ph), 95.45 (*C*Cl$_3$, Troc), 91.54 (C-1), 74.75 (*C*H$_2$, Troc), 73.98 (C-4, $^{2}J_{C4,P}$ = 6.4 Hz), 73.47 (*C*H$_2$Ph), 71.26 (C-3), 69.67 (*C*H$_2$, $^{2}J_{C,P}$ = 5.8 Hz, OP(O)(O*C*H$_2$Ph)$_2$), 69.58 (*C*H$_2$, $^{2}J_{C,P}$ = 5.8 Hz, OP(O)(O*C*H$_2$Ph)$_2$), 69.37 (C-5, $^{3}J_{C5,P}$ = 5.8 Hz), 68.56 (C-6), 54.10 (C-2), 37.56 (*C*H$_2$, Lev), 29.69 (*C*H$_3$, Lev), 27.90 (*C*H$_2$, Lev);

^{31}P-NMR (121 MHz, $CDCl_3$, α-anomer): δ -2.34;

HRMS (+ESI) m/z: calcd for $C_{35}H_{40}Cl_3NO_{12}P$ $[M+H]^+$ 802.1348, found 802.1348.

Synthesis of 14-17

(*R*)-3-(alkanoyloxy)alkanoic acids **14-17** were prepared according to the reported procedures.[2,25]

Synthesis of 18

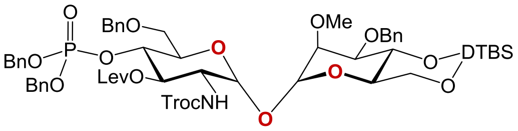

3-*O*-Benzyl-4,6-*O*-di-*tert*-butylsilylene-2-*O*-methyl-α-D-mannopyranosyl-(1↔1)-6-*O*-benzyl-4-*O*-[bis(benzyloxy)phosphoryl]-2-deoxy-3-*O*-levulinoyl-2-(2,2,2-trichloroethoxycarbonylamino)-α-D-glucopyranoside (18)

To a stirred solution of a glycosyl donor **8** (641 mg, 1076 µmol) and a glycosyl acceptor **13** (639 mg, 796 µmol) in dry DCM (15 mL) grinded activated 4 Å molecular sieves were added and the mixture was stirred for 2 h at r.t. The mixture was cooled to 0 °C and a solution of TMSOTf (10 µL, 55 µmol) in dry DCM [100 µL of a freshly prepared stock solution of TMSOTf (50 µL) in DCM (500 µL)] was added. The reaction mixture was stirred at 0 °C for 30 min and subsequently quenched by addition of Et_3N (10 µL). The mixture was warmed to r.t., diluted with EtOAc (100 mL), the solids were removed by filtration over Celite, and the filtrate was washed with aq. sat. $NaHCO_3$ (50 mL) and brine (50 mL). The organic layer was dried over Na_2SO_4, filtered and concentrated. The residue was purified by column chromatography on silica gel (toluene - EtOAc, 3:1 → 0:1 and hexane – EtOAc, 2:1 → 1:1) to afford **18** (496 mg, 410 µmol, 52%) as a colorless syrup. R_f = 0.41 (hexane – EtOAc, 1:1); $[\alpha]_D^{20}$ = +64 (*c* 1.0, $CHCl_3$);

^{1}H-NMR (600 MHz, $CDCl_3$): δ 7.35 (d, 2H, *J* = 7.5 Hz, CH$_2$Ph), 7.29-7.15 (m, 18H, CH$_2$Ph), 5.13 (dd, 1H, ^{3}J = 9.3 Hz, ^{3}J = 10.7 Hz, H-3), 5.08 (d, 1H, $^{3}J_{1,2}$ = 3.7 Hz, H-1), 5.01 (d, 1H, $^{3}J_{1',2'}$ = 1.3 Hz, H-1′), 4.96-4.88 (m, 7H, N*H*, CH_2Ph, OP(O)(OCH_2Ph)$_2$), 4.71 (AB, 1H, ^{2}J = 12.1 Hz, CH_2, Troc), 4.48 (AB, 1H, ^{2}J = 12.1 Hz, CH_2, Troc), 4.43-38 (m, 1H, H-4), 4.42 (AB, 1H, ^{2}J = 11.8 Hz, CH_2Ph), 4.34 (AB, 1H, ^{2}J = 11.8 Hz, CH_2Ph), 4.23 (t, 1H, $^{3}J_{4',3'}$ = $^{3}J_{4',5'}$ = 9.5 Hz, H-4′), 4.00 (ddd, 1H, $^{3}J_{2,1}$ = 3.6 Hz, *J* = 9.5 Hz, *J* = 10.7 Hz, H-2), 3.93 (dd, 1H, $^{3}J_{6a',5'}$ = 4.9, $^{2}J_{6a',6b'}$ = 10.1Hz, H-6a′), 3.86 (t, 1H, $^{3}J_{6b',5'}$ = $^{2}J_{6b',6a'}$ = 10.2 Hz, H-6b′), 3.68-3.66 (m, 2H, H-5, H-6a), 3.62-3.56 (m, 1H, H-5′) 3.59 (dd, 1H, $^{3}J_{3',2'}$ = 3.2 Hz, $^{3}J_{3',4'}$ = 9.5 Hz, H-3′), 3.53 (dd, 1H, *J* = 6.1 Hz, *J* = 11.3 Hz, H-6b), 3.30 (s, 3H, CH_3, Me), 3.29-3.28 (m, 1H, H-2′), 2.54-2.39 (m, 4H, 2×CH_2, Lev), 2.03 (s, 3H, CH_3, Lev), 0.99 (s, 9H, 3×CH_3, DTBS), 0.94 (s, 9H, 3×CH_3, DTBS);

^{13}C-NMR (151 MHz, $CDCl_3$): δ 205.73 (*C*O, Lev), 173.20 (*C*O, Lev), 154.22 (*C*O, Troc), 138.74, 137.75 (2×*C*q, CH$_2$Ph), 135.51 (*C*q, $^{3}J_{C,P}$ = 3.4 Hz, OP(O)(OCH$_2$Ph)$_2$), 135.46 (*C*q, $^{3}J_{C,P}$ = 3.9 Hz, OP(O)(OCH$_2$Ph)$_2$), 128.73,

128.71, 128.65, 128.37, 128.34, 128.06, 128.02, 127.97, 127.74, 127.69, 127.63 (20×*C*H, CH_2Ph), 95.30 (*C*Cl_3, Troc), 93.90 (C-1′, $^{1}J_{C,H}$ = 172 Hz), 93.27 (C-1, $^{1}J_{C,H}$ = 174 Hz), 79.69 (C-2′), 77.84 (C-3′), 74.70 (*C*H_2, Troc), 74.66 (C-4′), 74.15, 73.55 (2x*C*H_2, *C*H_2Ph), 73.45 (C-4, $^{2}J_{C4,P}$ = 6.1 Hz), 71.29 (C-3), 70.67 (C-5, $^{3}J_{C5,P}$ = 5.6 Hz), 69.76 (*C*H_2, $^{2}J_{C,P}$ = 5.2 Hz, OP(O)(O*C*H_2Ph)$_2$), 69.73 (*C*H_2, $^{2}J_{C,P}$ = 4.4 Hz, OP(O)(O*C*H_2Ph)$_2$), 69.34 (C-5′), 68.24 (C-6), 66.31 (C-6′), 59.65 (*C*H_3, Me), 53.76 (C-2), 37.56 (*C*H_2, Lev), 29.69 (*C*H_3, Lev), 27.90 (*C*H_2, Lev), 27.46, 27.12 (6×*C*H_3, DTBS), 22.58, 19.88 (2×*C*q, DTBS);

^{31}P-NMR (243 MHz, $CDCl_3$): δ -2.0;

HRMS (+ESI) m/z: calcd for $C_{57}H_{74}Cl_3NO_{17}PSi$ $[M+H]^+$ 1208.4325, found 1208.3533.

Synthesis of 19

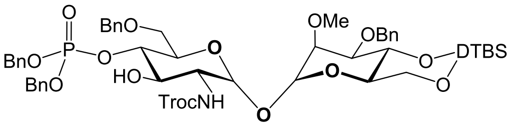

3-*O*-Benzyl-4,6-*O*-di-*tert*-butylsilylene-2-*O*-methyl-α-D-mannopyranosyl-(1↔1)-6-*O*-benzyl-4-*O*-[bis(benzyloxy)phosphoryl]-2-deoxy-2-(2,2,2-trichloroethoxycarbonylamino)-α-D-glucopyranoside (19).

To a stirred solution of **18** (463 mg, 360 µmol) in a mixture of pyridine – AcOH (7 mL, 3:2) hydrazine monohydrate (35 µL, 720 µmol) was added. The reaction mixture was stirred for 50 min at r.t. and acetone (2 mL) was added. The mixture was stirred for 10 min, diluted with EtOAc (50 mL) and washed with aq. citric acid (0.25 M, 70 mL), aq. sat. $NaHCO_3$ (70 mL) and brine (50 mL). The organic layer was dried over Na_2SO_4, filtered and concentrated. The residue was purified by column chromatography on silica gel (hexane – EtOAc, 2:1 → 1:1) to give **19** (340 mg, 305 µmol, 85%) as a colorless syrup. R_f = 0.46 (hexane – EtOAc, 1 : 1); $[\alpha]_D^{20}$ = +68 (*c* 0.9, $CHCl_3$);

^{1}H-NMR (600 MHz, $CDCl_3$): δ 7.41-7.23 (20×C*H*, CH_2Ph), 5.21 (d, 1H, $^{3}J_{1,2}$ = 3.5 Hz, H-1), 5.08-4.99 (m, 7H, H-1′, 3×C*H$_2$*, OP(O)(OC*H$_2$*Ph)$_2$, C*H$_2$*Ph), 4.88 (d, 1H, $^{3}J_{NH,2}$ = 8.2 Hz, N*H*), 4.77 (AB, 1H, ^{2}J = 12.0 Hz, C*H$_2$*, Troc), 4.53 (AB, 1H, ^{2}J = 12.0 Hz, Troc), 4.46 (s, 2H, C*H$_2$*Ph), 4.31 (t, 1H, $^{3}J_{4',3'}$ = $^{3}J_{4',5'}$ = 9.5 Hz, H-4′), 4.24 (d, 1H, $^{3}J_{4,3}$ = $^{3}J_{4,5}$ = $^{3}J_{H,P}$ = 8.4 Hz, H-4), 4.01 (dd, 1H, $^{3}J_{6a',5'}$ = 5.0 Hz, $^{2}J_{6a',6b'}$ = 10.1 Hz, H-6a′), 3.93 (t, 1H, $^{3}J_{6b',5'}$ = $^{2}J_{6b',6a'}$ = 10.2 Hz, H-6b′), 3.92-3.88 (m, 1H, H-2), 3.76-3.72 (m, 1H, H-3), 3.68-3.62 (m, 3H, H-5, H-5′, H-6a), 3.59 (dd, 1H, $^{3}J_{3',2'}$ = 3.1 Hz, $^{3}J_{3',4'}$ = 9.5 Hz, H-3′), 3.53 (dd, 1H, *J* = 5.4 Hz, *J* = 10.9 Hz, H-6b), 3.39 (s, 3H, C*H$_3$*, Me), 3.32 (br. s, 1H, H-2′), 1.07 (s, 9H, 3×C*H$_3$*, DTBS), 1.02 (s, 9H, 3×C*H$_3$*, DTBS);

^{13}C-NMR (151 MHz, $CDCl_3$): δ 154.78 (*C*O, Troc), 138.79, 137.71 (2×*C*q, CH_2Ph), 135.22, 135.18 (2×*C*q, OP(O)(OCH_2Ph)$_2$), 128.95, 128.81, 128.75, 128.66, 128.36, 128.27, 127.94, 127.91, 127.73, 127.57 (20×*C*H, CH_2Ph), 95.30 (*C*Cl_3, Troc), 93.82 (C-1′), 93.33 (C-1), 79.83 (C-2′), 77.49 (C-3′), 77.17 (C-4), 74.74 (C-4′), 74.64, 73.86, 73.63 (2×*C*H_2Ph, *C*H_2 Troc), 70.80 (C-3), 70.54 (*C*H_2, $^{2}J_{C,P}$ = 5.7 Hz, OP(O)(O*C*H_2Ph)$_2$), 70.31 (C-5, $^{3}J_{C5,P}$ = 9.6 Hz), 70.07 (*C*H_2, $^{2}J_{C,P}$ = 5.7 Hz, OP(O)(O*C*H_2Ph)$_2$), 69.30 (H-5′), 68.27 (C-6), 66.34 (C-6′), 59.58 (CH_3, Me), 54.91 (C-2), 27.48, 27.14 (6×*C*H_3, DTBS), 22.61, 19.89 (2×*C*q, DTBS);

^{31}P-NMR (243 MHz, $CDCl_3$): δ 0.52;

HRMS (-ESI) m/z: calcd for $C_{52}H_{57}Cl_4NO_{15}PSi$ $[M+Cl]^-$ 1144.2777, found 1144.2781.

Synthesis of 20

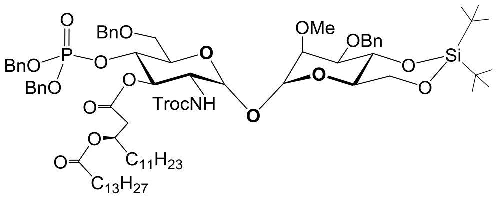

3-*O*-Benzyl-4,6-*O*-di-*tert*-butylsilylene-*O*-methyl-α-D-mannopyranosyl-(1↔1)-6-*O*-benzyl-4-*O*-[bis(benzyloxy)phosphoryl]-2-deoxy-3-*O*-[(*R*)-3-(tetradecanoyloxy)tetradecanoyl]-2-(2,2,2-trichloroethoxycarbonylamino)-α-D-glucopyranoside (20)

To a stirred solution of **19** (338 mg, 304 µmol) and DMAP (3.7 mg, 30 µmol) in dry DCM (5 mL) a solution of (*R*)-3-(tetradecanoyloxy)tetradecanoic acid **14** (243 mg, 533 µmol) in dry DCM (2 mL) and a solution of DIC (75 mg, 518 µmol) in dry DCM (2 mL) were gradually added over a period of 6 h at 0° C. The reaction mixture was additionally stirred for 2 h at 0° C, and subsequently diluted with DCM (150 mL), washed with aq. citric acid (0.25 M, 100mL), aq. sat. $NaHCO_3$ (100mL) and brine (100 mL). The organic layer was dried over Na_2SO_4, filtered and concentrated. The residue was purified by column chromatography on silica gel (hexane – EtOAc, 4:1 → 3:1) to afford **20** (380 mg, 118µmol, 81%) as a colorless syrup. R_f = 0.50 (hexane – EtOAc, 2:1); $[\alpha]_D^{20}$ = +44 (*c* 1.0, $CHCl_3$);

^{1}H-NMR (600 MHz, $CDCl_3$): δ 7.43-7.41 (m, 2×C*H*, CH_2Ph), 7.36-7.22 (m, 18×C*H*, CH_2Ph), 5.53 (d, 1H, $^3J_{NH,2}$ = 8.1 Hz, N*H*), 5.23 (d, 1H, $^3J_{1,2}$ = 3.6 Hz, H-1), 5.20 (dd, 1H, $^3J_{3,4}$ = 9.1 Hz, $^3J_{3,2}$ = 11.1 Hz, H-3), 5.14-5.10 (m, 1H, β^{Myr}-C*H*), 5.07 (d, 1H, $^3J_{1',2'}$ = 1.6 Hz, H-1′), 5.04-4.93 (m, 6H, 3×CH_2, OP(O)(OCH_2Ph)$_2$, CH_2Ph), 4.77 (AB, 1H, ^2J = 11.9 Hz, CH_2, Troc), 4.48 (AB, 1H, ^2J = 11.9 Hz, CH_2, Troc), 4.48 (AB, 1H, ^2J = 11.9 Hz, CH_2Ph), 4.47-4.42 (m, 1H, H-4), 4.39 (AB, 1H, ^2J = 11.9 Hz, CH_2Ph), 4.30 (t, 1H, $^3J_{4',3'}$ = $^3J_{4',5'}$ = 9.5 Hz, H-4′), 4.03 (ddd, 1H, $^3J_{2,1}$ = 3.5 Hz, $^3J_{2,3}$ = 11.3, $^3J_{2,NH}$ = 8.1 Hz, H-2), 3.98 (dd, 1H, $^3J_{6a',5'}$ = 5.1 Hz, $^2J_{6a',6b'}$ = 10.2 Hz, H-6a′), 3.93 (t, 1H, $^3J_{6b',5'}$ = $^2J_{6b',6a'}$ = 10.2 Hz, H-6b), 3.77-3.74 (m, 2H, H-5, H-6a), 3.69 (dd, 1H, $^3J_{3',2'}$ = 3.2 Hz, $^3J_{3',4'}$ = 9.5 Hz, H-3′), 3.69-3.66 (m, 1H, H-5′), 3.59 (dd, *J* = 5.9 Hz, *J* = 11.0 Hz, H-6b), 3.36 (s, 3H, CH_3, Me), 3.34 (dd, $^3J_{2',1'}$ = 1.6 Hz, $^3J_{2',3'}$ = 3.1 Hz, H-2′), 2.48 (dd, 1H, ^3J = 8.0 Hz, ^2J = 15.9 Hz, α^{Myr}-CH_2), 2.40 (dd, 1H, ^3J = 4.5 Hz, ^2J = 16.0 Hz, α^{Myr}-CH_2), 2.19 (t, 2H, *J* = 7.7 Hz, α^{Myr}-CH_2), 1.57-1.47 (m, 4H, β^{Myr}-CH_2, γ^{Myr}-CH_2), 1.30-1.22 (m, 40H, 20×CH_2), 1.06 (s, 9H, 3×CH_3, DTBS), 1.02 (s, 9H, 3×CH_3, DTBS), 0.89-0.87 (m, 6H, 2×ω^{Myr}-CH_3);

^{13}C-NMR (151 MHz, $CDCl_3$): δ 173.20, 170.97 (2×*C*O), 154.36 (*C*O, Troc), 138.79, 137.83 (2×*C*q, CH_2Ph), 135.53, 135.48 (2×*C*q, OP(O)(OCH$_2$Ph)$_2$), 128.72, 128.71, 128.63, 128.37, 128.32, 128.18, 128.04, 127.97, 127.70, 127.66, 127.59 (20x*C*H, CH_2Ph), 95.39 (*C*Cl$_3$, Troc), 94.06 (C-1′), 93.08 (C-1), 79.78 (C-2′), 78.06 (C-3′), 74.75 (C-4′), 74.42, 74.29 (2×*C*H$_2$, *C*H$_2$Ph), 73.63 (C-4, $^2J_{C4,P}$ = 6.0 Hz), 73.47 (*C*H$_2$, Troc), 70.73 (C-5, $^3J_{C5,P}$ = 5.8 Hz), 70.64 (C-3, $^3J_{C3,P}$ = 2.1 Hz), 70.00 (β^{Myr}-*C*H), 69.79 (*C*H$_2$, $^2J_{C,P}$ = 6.0 Hz, OP(O)(O*C*H$_2$Ph)$_2$), 69.74 (*C*H$_2$, $^2J_{C,P}$ = 5.2 Hz, OP(O)(O*C*H$_2$Ph)$_2$), 69.21 (C-5′), 68.25 (C-6), 66.32 (C-6′), 59.68 (*C*H$_3$, Me), 53.88 (C-2), 39.10 (α^{Myr}-*C*H$_2$), 34.42 (α^{Myr}-*C*H$_2$), 34.23 (γ^{Myr}-*C*H$_2$), 31.91, 29.70, 29.68, 29.66, 29.64, 29.62, 29.57, 29.55, 29.54, 29.40, 29.35, 29.31, 29.19 (17×*C*H$_2$), 27.49, 27.16 (6×CH$_3$, DTBS), 25.15, 25.01, 22.68 (3×*C*H$_2$), 22.61, 19.86 (2×*C*q, DTBS), 14.10 (2×ω^{Myr}-*C*H$_3$);

^{31}P-NMR (243 MHz, $CDCl_3$): δ -1.64;

HRMS (+ESI) m/z: calcd for $C_{80}H_{120}Cl_3NO_{18}PSi$ $[M+H]^+$ 1546.7072, found 1546.7067;

Synthesis of 21

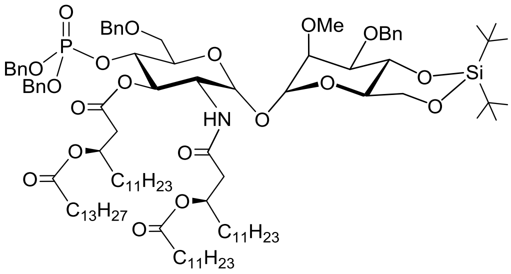

3-*O*-Benzyl-4,6-*O*-di-*tert*-butylsilylene-2-*O*-methyl-α-D-mannopyranosyl-(1↔1)-6-*O*-Benzyl-4-*O*-[bis(benzyloxy)phosphoryl]-2-deoxy-2-[(*R*)-3-(dodecanoyloxy)tetradecanonylamino]-3-*O*-[(*R*)-3-(tetradecanoyloxy)tetradecanoyl]-α-D-glucopyranoside (21).

To a solution of **20** (338 mg, 218 µmol) in dry DCM (4 mL) zinc dust (1 g) and AcOH (4 mL) were added at r.t. The mixture was vigorously stirred for 90 min, diluted with DCM (100 mL), the solids were removed by filtration over Celite and the filtrate was washed with aq. sat. $NaHCO_3$ (50 mL) and brine (50 mL). The organic layer was dried over Na_2SO_4, filtered and concentrated to afford crude amine (302 mg) as a colourless syrup. R_f= 0.28 (hexane – EtOAc, 1 : 1).

^{1}H-NMR (600 MHz, $CDCl_3$) δ 7.43-7.41 (m, 2H, CH_2Ph), 7.36-7.21 (m, 18H, CH_2Ph), 5.27 (br. s, 1H, $β^{Myr}$-C*H*), 5.22-5.11 (m, 3H, H-1, H-1′, H-3), 5.01 (AB, 1H, ^2J = 11.9 Hz, CH_2Ph), 4.96-4.93 (m, 4H, 2×CH_2, OP(O)(OCH_2Ph)$_2$), 4.77 (AB, 1H, ^2J = 11.9 Hz, CH_2Ph), 4.48 (AB, 1H, ^2J = 11.8 Hz, CH_2Ph), 4.39 (AB, 1H, ^2J = 11.8 Hz, CH_2Ph), 4.37 (q, 1H, $^3J_{4,3}$ = $^3J_{4,5}$ = $^3J_{H,P}$ = 9.3 Hz, H-4), 4.30 (t, 1H, $^3J_{4',3'}$ = $^3J_{4',5'}$ = 9.5 Hz, H-4′), 4.13-4.05 (br s, 1H, H-6a′), 3.94 (t, $^3J_{6b',5'}$ = $^2J_{6b',6a'}$ = 10.2 Hz, H-6b′), 3.85 (dt, 1H, $^3J_{5',6a'}$ = 4.7 Hz, $^3J_{5',6b'}$ = $^3J_{5',4'}$ = 9.9 Hz, H-5′), 3.78 (ddd, 1H, $^3J_{5,6a}$ = 1.8 Hz, $^3J_{5,6b}$ = 5.8 Hz, $^3J_{5,4}$ = 10.0 Hz, H-5), 3.73 (dd, 1H, $^3J_{6a,5}$ = 1.8 Hz, $^2J_{6a,6b}$ =10.8 Hz, H-6a), 3.69 (dd, 1H, $^3J_{3',2'}$ = 3.2 Hz, $^3J_{3',4'}$ = 9.5 Hz, H-3′), 3.57 (dd, 1H, $^3J_{6b,5}$ = 5.7 Hz, $^2J_{6b,6a}$ = 10.9 Hz, H-6b), 3.41 (dd, 1H, $^3J_{2',1'}$ = 1.7 Hz, $^3J_{2',3'}$ = 3.2 Hz, H-2′), 3.39 (s, 3H, CH_3, OMe), 2.60-2.55 (m, 1H, $α^{Myr}$-CH_2), 2.45-2.38 (m, 1H, $α^{Myr}$-CH_2), 2.23-2.22 (t, 2H, J = 7.6 Hz, $α^{Myr}$-CH_2), 1.60-1.47 (m, 4H, $β^{Myr}$-CH_2, $γ^{Myr}$-CH_2), 1.30-1.24 (m, 38H, 19×CH_2,), 1.06 (s, 9H, 3×CH_3, DTBS), 0.98 (s, 9H, 3×CH_3, DTBS), 0.89- 0.86 (m, 6H, 2$ω^{Myr}$-CH_3);
^{31}P-NMR (243MHz, $CDCl_3$) δ -1.73;
HRMS (+ESI) m/z: calcd for $C_{77}H_{119}NO_{16}PSi$ $[M+H]^+$ 1371.8030, found 1371.8009;
The crude amine (302 mg, 218 µmol) was dissolved in dry $CHCl_3$ (700 µL) and a solution of (*R*)-3-(dodecanoyloxy)tetradecanoic acid **15** (110 mg, 264 µmol) in $CHCl_3$ (2 mL) and a solution of EDC•HCl (75 mg, 330 µmol) in $CHCl_3$ (2 mL) were added gradually over 3 h at r.t. The mixture was additionally stirred for 1 h, diluted with $CHCl_3$ (100 mL), washed with brine – H_2O (1:1, 70 mL) and brine (70 mL). The organic layer was dried over Na_2SO_4, filtered and concentrated. The residue was purified by column chromatography on silica gel (hexane – EtOAc, 3:1 → 1:1) to afford **21** (323 mg, 181 µmol, 83% for 2 steps) as a colorless syrup. R_f = 0.44 (hexane – EtOAc, 2 : 1, v/v); $[α]_D^{20}$ = +35 (*c* 1.0, $CHCl_3$);

^{1}H-NMR (600 MHz, $CDCl_3$): δ 7.43-7.42 (m, 2H, CH_2Ph), 7.35-7.22 (m, 18H, CH_2Ph), 6.10 (d, 1H, $^3J_{NH,2}$ = 7.9 Hz, N*H*), 5.23-5.12 (m, 2H, 2×$β^{Myr}$-C*H*), 5.20 (d, 1H, $^3J_{1,2}$ = 3.6 Hz, H-1), 5.17 (dd, $^3J_{3,4}$ = 9.0 Hz, $^3J_{3,2}$ = 11.1 Hz, H-3), 5.08 (d, 1H, $^3J_{1',2'}$ = 1.5 Hz, H-1′), 5.01 (AB, 1H, ^2J = 11.9 Hz, CH_2Ph), 4.96-4.92 (m, 4H, 2×CH_2, OP(O)(OCH_2Ph)$_2$), 4.77 (AB, 1H, ^2J = 11.9 Hz, CH_2Ph), 4.48 (AB, 1H, ^2J = 11.8 Hz, CH_2Ph), 4.39 (AB, 1H, ^2J = 11.8 Hz, CH_2Ph), 4.38-4.35 (m, 1H, H-4), 4.30 (t, $^3J_{4',3'}$ = $^3J_{4',5'}$ = 9.5 Hz, H-4′), 4.25 (ddd, 1H, $^3J_{2,1}$ = 3.6 Hz, $^3J_{2,NH}$ = 7.8 Hz, $^3J_{2,3}$ = 11.2 Hz, H-2), 4.03 (dd, 1H, $^3J_{6a',5'}$ = 5.0 Hz, $^2J_{6a',6b'}$ = 10.3 Hz, H-6a′), 3.93 (t, 1H, $^3J_{6b',5'}$ = $^2J_{6b',6a'}$ = 10.4 Hz, H-6b′), 3.76-3.74 (m, 2H, H-5, H-6a), 3.71 (dd, 1H, $^3J_{3',2'}$ = 3.3 Hz, $^3J_{3',4'}$ = 9.6 Hz, H-3′), 3.63 (td, 1H, $^3J_{5',6a'}$ = 4.9 Hz, $^3J_{5',4'}$ = $^3J_{5',6b'}$ = 9.9 Hz, H-5′), 3.56 (dd, 1H, J = 6.1 Hz, J = 10.8 Hz, H-6b), 3.39 (dd, 1H, $^3J_{2',1'}$ = 1.6 Hz, $^3J_{2',3'}$ = 3.2 Hz, H-2′), 3.34 (s, 3H, CH_3, Me), 2.48-2.32 (m, 4H, 2×$α^{Myr}$-CH_2), 2.28-2.19 (m, 4H, $α^{Myr}$-CH_2, $α^{Lau}$-CH_2), 1.60-1.46 (m, 8H, 2×$γ^{Myr}$-CH_2, $β^{Myr}$-CH_2, $β^{Lau}$-CH_2), 1.28-1.23 (m, 72H, 36×CH_2, fatty acids), 1.07 (s, 9H, 3×CH_3, DTBS), 1.01 (s, 9H, 3×CH_3, DTBS), 0.89-0.86 (m, 12H, 3×$ω^{Myr}$-CH_3, $ω^{Lau}$-CH_3);

^{13}C-NMR (151 MHz, $CDCl_3$) δ 173.53, 173.26, 171.26, 169.79 (4×*C*O), 138.90, 137.89 (2×*C*q, CH_2Ph) 135.56, 135.51 (2×*C*q, OP(O)($OCH_2Ph)_2$) 128.71, 128.63, 128.36, 128.31, 128.18, 128.05, 127.85, 127.63, 127.62, 127.59 (20×*C*H, CH_2Ph), 93.80 (C-1′), 93.11 (C-1), 79.74 (C-2′), 78.25 (C-3′), 74.65 (C-4′), 74.11 (*C*H_2Ph), 73.86 (C-4), 73.47 (*C*H_2Ph), 70.90, 70.76 (C-3, C-5), 70.09, 70.01 (2×β^{Myr}-*C*H), 69.77, 69.75, 69.74, 69.72 (2×CH_2, OP(O)(O$CH_2Ph)_2$), 69.33 (C-5′), 68.44 (C-6), 66.36 (C-6′), 59.57 (*C*H_3, Me), 51.66 (C-2), 40.95, 39.29 (2×α^{Myr}-*C*H_2), 34.58, 34.46 (α^{Myr}*C*H_2, α^{Lau}*C*H_2, 2×γ^{Myr}*C*H_2), 31.93, 31.92, 29.72, 29.69, 29.66, 29.62, 29.57, 29.52, 29.39, 29.36, 29.25, 29.22, 25.26, 25.19, 25.05, 25.04, 22.68 (38×*C*H_2) 27.53, 27.29 (6×CH_3, DTBS), 22.64, 19.87 (2×*C*q, DTBS), 14.09 (3×ω^{Myr}-*C*H_3, ω^{Lau}-*C*H_3);

^{31}P-NMR (242.97MHz, $CDCl_3$): δ -1.67;

HRMS (+ESI) m/z: calcd for $C_{103}H_{166}NNaO_{19}PSi$ $[M+Na]^+$ 1780.1453, found 1780.1430.

Synthesis of 22

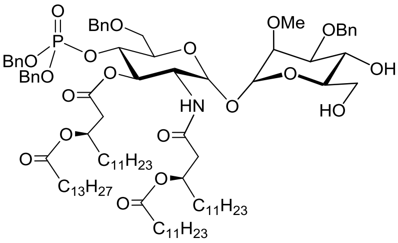

3-*O*-Benzyl-2-*O*-methyl-α-D-mannopyranosyl-(1↔1)-6-*O*-benzyl-4-*O*-[bis(benzyloxy)phosphoryl]-2-deoxy-2-[(*R*)-3-(dodecanoyloxy)tetradecanoylamino]-3-*O*-[(*R*)-3-(tetradecanoyloxy)tetradecanoyl]-α-D-glucopyranoside (22).

To a stirred solution of **21** (323 mg, 181 μmol) in dry THF (7 mL) in a PTFE-vial, a solution of HF•pyridine (100 μL, 70%) was added at 0 °C. The reaction mixture was stirred at 0 °C for 10 min, then warmed to r.t. and stirred for 30 min. The reaction mixture was diluted with $CHCl_3$ (150 mL) and washed with aq. sat. $NaHCO_3$ (70 mL) and brine (70 mL). The organic layer was dried over Na_2SO_4, filtered and concentrated. The residue was purified by column chromatography on silica gel (toluene – EtOAc, 2:3) to afford **22** (291 mg, 177 μmol, 98%). R_f = 0.22 (toluene – EtOAc, 1 : 1); $[\alpha]_D^{20}$ = +40.5 (*c* 1.0, $CHCl_3$);

^{1}H-NMR (600MHz, $CDCl_3$) δ = 7.43-7.22 (m, 20H, CH_2Ph), 6.34 (d, 1H, $^3J_{NH,2}$ = 8.2 Hz, N*H*), 5.26 (d, 1H, $^3J_{1,2}$ = 3.7 Hz, H-1), 5.22 (dd, 1H, $^3J_{3,4}$ = 9.1 Hz, $^3J_{3,2}$ = 11.0 Hz, H-3), 5.14-5.10 (m, 1H, β^{Myr}-C*H*), 5.13 (d, 1H, $^3J_{1',2'}$ = 1.6 Hz, H-1′), 5.05-5.01 (m, 1H, β^{Myr}-C*H*), 4.97-4.93 (m, 4H, 2×C*H_2*, OP(O)(OC*H_2*Ph$)_2$), 4.79 (AB, 1H, ^2J = 11.6 Hz, C*H_2*Ph), 4.73 (AB, 1H, ^2J = 11.7 Hz, C*H_2*Ph), 4.49 (AB, 1H, ^2J = 11.8 Hz, C*H_2*Ph), 4.42 (q, 1H, $^3J_{4,3}$ = $^3J_{4,5}$ = $^3J_{H,P}$ = 9.2 Hz, H-4), 4.40 (AB, 1H, ^2J = 11.9 Hz, C*H_2*Ph), 4.26 (ddd, 1H, $^3J_{1,2}$ = 3.5 Hz, $^3J_{2,NH}$ = 8.0 Hz, $^3J_{2,3}$ = 11.2 Hz, H-2), 3.92-3.88 (m, 1H, H-4′), 3.84-3.79 (m, 2H, H-3′, H-6a′), 3.76-3.74 (m, 3H, H-5, H-6a, H-6b′), 3.60-3.56 (m, 2H, H-5′, H-6b), 3.30 (s, 3H, C*H_3*, Me), 3.30-3.28 (m, 1H, H-2′), 2.70 (s, 1H, OH), 2.48-2.42 (m, 3H, α^{Myr}-C*H_2*), 2.39 (dd, 1H, *J* = 6.2 Hz, *J* = 15.3 Hz, α^{Myr}-C*H_2*), 2.34-2.26 (m, 2H, α^{Lau}-C*H_2*), 2.23-2.20 (m, 2H, α^{Myr}-C*H_2*), 1.65-1.46 (m, 8H, 2×γ^{Myr}C*H_2*, β^{Myr}C*H_2*, β^{Lau}C*H_2*), 1.31-1.21 (m, 72H, 36×C*H_2*), 0.89-0.86 (m, 12H, 3×ω^{Myr}-C*H_3*, ω^{Lau}-C*H_3*);

^{13}C-NMR (151 MHz, $CDCl_3$) δ 174.65, 173.48, 171.15, 170.08 (4×CO), 138.10, 137.87 (2×Cq, CH_2Ph), 135.47 (2×Cq, OP(O)($OCH_2Ph)_2$), 128.72, 128.63, 128.60, 128.31, 128.17, 128.04, 128.01, 127.63, 127.59 (20×*C*H, CH_2Ph), 93.14 (C-1′), 92.56 (C-1), 78.78 (C-3′), 77.38 (C-2′), 73.76 (C-4, C-5′), 73.48, 72.76 (2×*C*H_2Ph), 71.33 (β^{Myr}-*C*H), 71.02 (C-3), 70.62 (C-5, $^3J_{C5,P}$ = 5.0 Hz), 70.01 (β^{Myr}-*C*H), 69.82 (2×*C*H_2, OP(O)(O*C*$H_2Ph)_2$), 68.42 (C-6), 67.84 (C-4′), 63.04 (C-6′), 59.03 (*C*H_3, Me), 51.70 (C-2), 41.50, 39.09 (2×α^{Myr}-*C*H_2), 34.74, 34.60, 34.41, 34.23 (α^{Myr}*C*H_2, α^{Lau}*C*H_2, 2×γ^{Myr}*C*H_2), 31.92, 31.91, 29.70, 29.68, 29.65, 29.61, 29.58, 29.54, 29.47, 29.43, 29.37, 29.34, 29.20, 29.18, 25.28, 25.17, 25.01, 24.99, 22.67 (38×*C*H_2), 14.08 (3×ω^{Myr}-*C*H_3, ω^{Lau}-*C*H_3);

^{31}P-NMR (243 MHz, $CDCl_3$) δ -1.80;

HRMS (+ESI) m/z: calcd for $C_{95}H_{150}NNaO_{19}P$ $[M+Na]^+$ 1663.0432, found 1663.0424.

Synthesis of 23

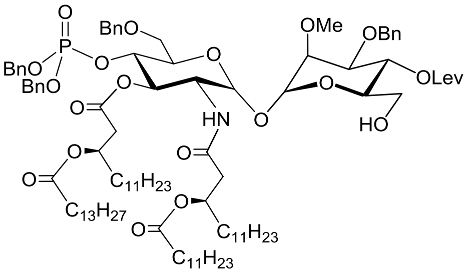

3-*O*-Benzyl-4-*O*-levulinoyl-2-*O*-methyl-α-D-mannopyranosyl-(1↔1)-6-*O*-benzyl-4-*O*-[bis(benzyloxy)phosphoryl]-2-deoxy-2-[(*R*)-3-(dodecanoyloxy)tetradecanoylamino]-3-*O*-[(*R*)-3-(tetradecanoyloxy)tetradecanoyl]-α-D-glucopyranoside (23).

To a stired solution of **22** (71 mg, 43 μmol), levulinic acid (11 mg, 94 μmol) and DMAP (0.3 mg, 2.5 μmol) in dry DCM (10 mL) a solution of DIC (13.8 mg, 109 μmol) in dry toluene (20 mg×mL^{-1}) was added over a period of 5 h via syringe pump at 0 °C. The mixture was diluted with DCM (100 mL), washed with aq. citric acid (0.25 M, 50 mL), aq. sat. $NaHCO_3$ (50 mL), and brine (50 mL). The organic layer was dried over Na_2SO_4, filtered and concentrated. The residue was purified by column chromatography on silica gel (hexane – EtOAc, 1:1 → 1:2) and by HPLC (column A: hexane – EtOAc, 1:1→1:2) to afford **23** (41 mg, 24 μmol, 55%) as a colorless syrup. R_f = 0.39 (toluene – EtOAc, 1:2); $[\alpha]_D^{20}$ = +34 (*c* 0.9, $CHCl_3$);

^{1}H-NMR (600 MHz, $CDCl_3$): δ 7.39-7.21 (m, 20H, CH_2Ph), 6.37 (d, 1H, $^2J_{NH,2}$ = 8.0 Hz, N*H*), 5.24-5.19 (m, 3H, H-1, H-3, H-4′), 5.18-5.14 (m, 1H, β^{Myr}-C*H*), 5.12 (d, 1H, $^3J_{1',2'}$ = 2.0 Hz, H-1′), 5.07-5.03 (m, 1H, β^{Myr}-C*H*), 4.97- 4.94 (m, 4H, OP(O)(OCH_2Ph)$_2$), 4.71 (AB, 1H, ^2J = 11.9 Hz, CH_2Ph), 4.66 (AB, 1H, ^2J = 12.0 Hz, CH_2Ph), 4.49 (AB, 1H, ^2J = 11.8 Hz, CH_2Ph), 4.43 (dt, *J* = 9.0 Hz, *J* = 9.6 Hz, H-4), 4.41 (AB, 1H, ^2J = 11.9 Hz, CH_2Ph), 4.25 (ddd, 1H, $^3J_{2,1}$ = 3.5 Hz, $^3J_{2,NH}$ = 7.9 Hz, $^3J_{2,3}$ = 11.2 Hz, H-2), 3.97 (dd, 1H, $^3J_{3',2'}$ = 3.1 Hz, $^3J_{3',4'}$ = 9.5 Hz, H-3′), 3.76-3.73 (m, 2H, H-5, H-6a), 3.65-3.62 (m, 1H, H-5′), 3.62-3.57 (m, 3H, H-6a′, H-6b′, H-6b), 3.32-3.31 (m, 1H, H-2′), 3.31 (s, 3H, CH_3, Me), 2.78 (ddd, 1H, *J* = 6.1 *J* = 7.6 Hz, *J* = 18.3 Hz, CH_2, Lev), 2.70 (dt, 1H, *J* = 6.1 Hz, *J* = 18.3 Hz , CH_2, Lev), 2.57 (ddd, 1H, *J* = 5.7 Hz, *J* = 7.8 Hz, *J* = 17.2 Hz, CH_2, Lev), 2.52-2.47 (m, 3H, α^{Myr}-CH_2, CH_2, Lev), 2.44 (dd, 1H, *J* = 7.0 Hz, *J* = 15.1 Hz, α^{Myr}-CH_2), 2.37 (dd, 1H, *J* = 5.6 Hz, *J* = 15.1 Hz, α^{Myr}-CH_2), 2.33-2.25 (m, 2H, α^{Lau}-CH_2), 2.20 (t, 2H, *J* = 7.6 Hz, α^{Myr}-CH_2), 2.16 (s, 3H, CH_3, Lev), 1.62-1.46 (m, 8H, β^{Myr}-CH_2, β^{Lau}-CH_2, 2×γ^{Myr}-CH_2), 1.32-1.22 (m, 72H, 36×CH_2), 0.89-0.86 (m, 12H, 3×ω^{Myr}-CH_3, ω^{Lau}-CH_3);

^{13}C-NMR (151 MHz, $CDCl_3$): δ 205.99 (*C*O, Lev), 173.76, 173.41, 172.54, 171.36, 169.93 (5×*C*O), 138.10, 137.89 (2×*C*q, CH_2Ph), 135.56, 135.51 (2×*C*q, OP(O)(OCH$_2$Ph)$_2$), 128.71, 128.64, 128.51, 128.32, 128.12, 128.03, 127.90, 127.64, 127.59 (20×*C*H, CH_2Ph), 94.10 (C-1′), 93.47 (C-1), 77.95 (C-2′), 76.00 (C-3′), 73.69 (C-4, $^2J_{C4,P}$ = 5.9 Hz), 73.48, 72.72 (2×*C*H$_2$, CH_2Ph), 72.70 (C-5′), 71.08 (C-3), 70.89 (β^{Myr}-*C*H), 70.69 (C-5, $^3J_{C5,P}$ = 5.4 Hz), 69.91 (β^{Myr}-*C*H), 69.76, 69.72 (2×*C*H$_2$, OP(O)(O*C*H$_2$Ph)$_2$), 68.60 (C-4′), 68.36 (C-6), 61.65 (C-6′), 59.20 (*C*H$_3$, Me), 51.78 (C-2), 41.43 (α^{Myr}-*C*H$_2$), 39.04 (α^{Myr}-*C*H$_2$), 37.89 (*C*H$_2$, Lev), 34.62, 34.52, 34.43, 34.27 (α^{Myr}-*C*H$_2$, α^{Lau}-*C*H$_2$, 2×γ^{Myr}-*C*H$_2$), 31.92, 29.71, 29.66, 29.64, 29.62, 29.58, 29.56, 29.51, 29.39, 29.36, 29.34, 29.25, 29.19, 27.92, 25.35, 25.17, 25.08, 25.00, 22.68 (39×*C*H$_2$, *C*H$_3$, Lev), 14.10 (3×ω^{Myr}-*C*H$_3$, ω^{Lau}-*C*H$_3$);

^{31}P-NMR (243 MHz, $CDCl_3$): δ -1.71;
HRMS (+ESI) m/z: calcd for $C_{100}H_{156}NNaO_{21}P$ $[M+Na]^+$ 1761.0800, found 1761.0804.

Synthesis of 24

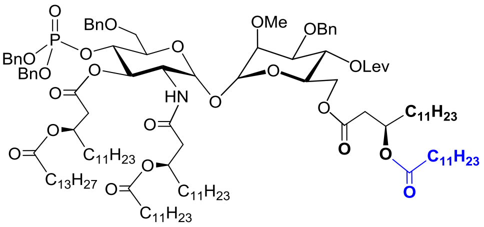

3-*O*-Benzyl-6-*O*-[(*R*)-3-(dodecanoyloxy)tetradecanoyl]-4-*O*-levulinoyl-2-*O*-methyl-α-D-mannopyranosyl-(1↔1)-6-*O*-benzyl-4-*O*-[bis(benzyloxy)phosphoryl]-2-deoxy-2-[(*R*)-3-(dodecanoyloxy)-tetradecanoylamino]-3-*O*-[(*R*)-3-(tetradecanoyloxy)tetradecanoyl]-α-D-glucopyranoside (24).

To a stirred solution of **23** (30 mg, 17 µmol) and DMAP (0.4 mg, 3 µmol) in dry DCM (500 µL) a solution of (*R*)-3-(dodecanoyloxy)tetradecanoic acid **15** (15 mg, 36 µmol) in dry DCM (500 µL) and a solution of DIC (9 mg, 72 µmol) in dry DCM (400 µL) were gradually added over 5h at 0 °C. The mixture was additionally stirred for 1 h, diluted with EtOAc (30 mL) and washed with aq. citric acid (0.25 M, 20 mL), aq. sat. $NaHCO_3$ (20 mL) and brine (20 mL). The organic layer was dried over Na_2SO_4, filtered and concentrated. The residue was purified by HPLC (column A: hexane – EtOAc, 3:1 → 2:1) to afford **24** (27 mg, 12.7 µmol, 74%). R_f = 0.38 (toluene – EtOAc, 4:1, v/v); $[\alpha]_D^{20}$ = +47 (*c* 1.0, $CHCl_3$);

^{1}H-NMR (600 MHz, $CDCl_3$): δ 7.39-7.21 (m, 20H, CH_2Ph), 6.41 (d,1H, $^3J_{NH,2}$ = 8.0 Hz, N*H*), 5.27-5.16 (m, 5H, H-1, H-3, H-4′, 2×β^{Myr}-C*H*), 5.12 (d, 1H, $^3J_{1',2'}$ = 1.9 Hz, H-1′), 5.08-5.04 (m, 1H, β^{Myr}-C*H*), 4.98-4.94 (m, 4H, OP(O)(OCH_2Ph$)_2$), 4.70 (AB, 1H, ^2J = 11.9 Hz, CH_2Ph), 4.65 (AB, 1H, ^2J = 11.9 Hz, CH_2Ph), 4.48 (AB, 1H, ^2J = 11.8 Hz, CH_2Ph), 4.46-4.41 (m, 1H, H-4), 4.40 (AB, 1H, ^2J = 11.9 Hz, CH_2Ph), 4.27-4.22 (m, 2H, H-2, H-6a′), 4.01-3.98 (m, 2H, H-3′, H-6b′), 3.81 (ddd, 1H, *J* = 2.0 Hz, *J* = 5.4 Hz, *J* = 10.0 Hz, H-5′), 3.75-3.71 (m, 2H, H-5, H-6a), 3.58 (dd, 1H, *J* = 5.8 Hz, *J* = 11.0 Hz, H-6b), 3.30 (s, 3H, CH_3, Me), 3.28 (t, 1H, *J* = 2.5 Hz, H-2′), 2.76-2.65 (m, 2H, CH_2, Lev), 2.61-2.47 (m, 6H, 2×α^{Myr}-CH_2, CH_2, Lev), 2.43 (dd, 1H, *J* = 7.3 Hz, *J* = 15.3 Hz, α^{Myr}-CH_2), 2.37 (dd, 1H, *J* = 5.0, *J* = 15.4 Hz, α^{Myr}-CH_2), 2.35-2.27 (m, 2H, α^{Lau}-CH_2), 2.23 (t, 2H, *J* = 7.6 Hz, α-CH_2), 2.19 (t, 2H, *J* = 7.6 Hz, α-CH_2), 2.15 (s, 3H, CH_3, Lev), 1.62-1.47 (m, 12H, β^{Myr}-CH_2, 2×β^{Lau}-CH_2, 3×γ^{Myr}-CH_2), 1.32-1.22 (m, 106H, 53×CH_2), 0.89-0.86 (m, 18H, 2×ω^{Lau}-CH_3, 4×ω^{Myr}-CH_3);

^{13}C NMR (151 MHz, $CDCl_3$) δ 205.95 (*C*O, Lev), 173.82, 173.37, 173.05, 171.65, 171.34, 170.31, 169.69 (7×*C*O), 138.10, 137.90 (2×*C*q, CH_2Ph), 135.56, 135.52 (2×*C*q, OP(O)(OCH_2Ph$)_2$), 128.70, 128.64, 128.50, 128.32, 128.16, 128.13, 128.02, 127.87, 127.62, 127.57 (20×*C*H, CH_2Ph), 92.98 (C-1′), 92.39 (C-1), 77.66 (C-2′), 76.24 (C-3′), 73.68 (C-4, $^2J_{C4,P}$ = 5.7 Hz), 73.47, 72.76 (2×*C*H_2Ph), 71.08 (C-3), 70.61 (C-5, β^{Myr}-*C*H), 70.10, 69.86 (C-5′, 2×β^{Myr}-*C*H), 69.77, 69.74 (OP(O)(O*C*H_2Ph$)_2$), 68.35 (C-6), 67.70 (C-4′), 62.41 (C-6′), 59.24 (*C*H_3, Me), 51.67 (C-2), 41.58, 38.95, 38.74 (3×α^{Myr}-*C*H_2), 37.90 (*C*H_2, Lev), 34.79, 34.53, 34.48, 34.45, 34.28, 34.03 (β^{Myr}-*C*H_2, 2×β^{Lau}-*C*H_2, 3×γ^{Myr}-*C*H_2), 31.93, 29.70, 29.67, 29.63, 29.57, 29.53, 29.47, 29.44, 29.40, 29.37, 29.29, 29.20, 27.94, 25.40, 25.19, 25.10, 25.01, 22.69 (57×*C*H_2, *C*H_3, Lev), 14.10 (2×ω^{Lau}-*C*H_3, 4×ω^{Myr}-*C*H_3);

^{31}P NMR (243 MHz, $CDCl_3$) δ -1.70;

HRMS (+ESI) m/z: calcd for $C_{126}H_{204}NNaO_{24}P$ $[M+Na]^+$ 2169.4403, found 2169.4383.

Synthesis of 25

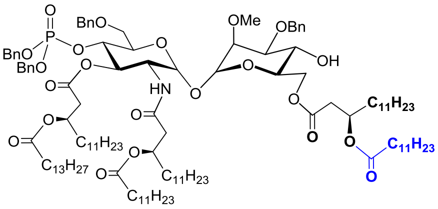

3-*O*-Benzyl-6-*O*-[(*R*)-3-(dodecanoyloxy)tetradecanoyl]-2-*O*-methyl-α-D-mannopyranosyl-(1↔1)-6-*O*-benzyl-4-*O*-[bis(benzyloxy)phosphoryl]-2-deoxy-2-[(*R*)-3-(dodecanoyloxy)tetradecanoylamino]-3-*O*-[(*R*)-3-(tetradecanoyloxy)tetradecanoyl]-α-D-glucopyranoside (25).

To a stirred solution of **24** (17 mg, 7.9 µmol) in pyridine – AcOH (5:4, 1 mL) hydrazine monohydrate (6 µL, 64% hydrazine) was added at r.t. The mixture was stirred for 1 h, then acetone (2 mL) was added and the mixture was stirred for 5 min. The solution was diluted with EtOAc (20 mL) and washed with 2 M aq. HCl (2 M, 15 mL), aq. sat. $NaHCO_3$ (15 mL) and brine (15 mL). The organic layer was dried over Na_2SO_4, filtered and concentrated. The residue was purified by HPLC (column B: hexane – EtOAc, 3:1 → 2:1) to afford **25** (14 mg, 6.8 µmol, 86%) as a colorless syrup. R_f = 0.34 (hexane - EtOAc, 2:1); $[\alpha]_D^{20}$ = +28 (*c* 0.73, $CHCl_3$);

^{1}H NMR (600 MHz, $CDCl_3$) δ 7.44-7.43 (m, 2H, CH_2Ph), 7.37-7.21 (m, 18H, CH_2Ph), 6.35 (d, 1H, $^3J_{NH,2}$ = 8.2 Hz, 2-N*H*), 5.24-5.19 (m, 3H, H-1, H-3, β^{Myr}-C*H*), 5.16-5.12 (m, 2H, H-1′, β^{Myr}-C*H*), 5.08-5.04 (m, 1H, β^{Myr}-C*H*), 4.96 (d, 2H, *J* = 8.1 Hz, OP(O)(OCH_2Ph)$_2$), 4.94 (d, 2H, *J* = 8.2 Hz, OP(O)(OCH_2Ph)$_2$), 4.85 (AB, 1H, ^2J = 11.7 Hz, CH_2Ph), 4.74 (AB, 1H, ^2J = 11.6 Hz, CH_2Ph), 4.48 (AB, 1H, ^2J = 11.8 Hz, CH_2Ph), 4.44-4.39 (m, 1H, H-4), 4.40 (AB, 1H, ^2J = 12.0 Hz, CH_2Ph), 4.35 (dd, 1H, $^3J_{6a',5'}$ = 5.0 Hz, $^2J_{6a',6b'}$ = 12.1 Hz, H-6a′), 4.27 (ddd, 1H, $^3J_{2,1}$ = 3.9 Hz, $^3J_{2,NH}$ = 8.3 Hz, $^3J_{2,3}$ = 10.9 Hz, H-2), 4.19 (dd, 1H, $^3J_{6b',5'}$ = 1.8 Hz, $^3J_{6b',6a'}$ = 12.0 Hz, H-6b′), 3.93 (t, 1H, $^3J_{4',3'}$ = $^3J_{4',5'}$ = 9.7 Hz, H-4′), 3.85 (dd, 1H, $^3J_{3',2'}$ = 3.2 Hz, $^3J_{3',4'}$ = 9.7 Hz, H-3′), 3.75-3.71 (m, 2H, H-5, H-6a), 3.67 (ddd, 1H, $^3J_{5',6b'}$ = 1.8 Hz, $^3J_{5',6a'}$ = 4.9 Hz, $^3J_{5',4'}$ = 9.7 Hz, H-5′), 3.58 (dd, 1H, *J* = 5.9 Hz, *J* = 10.9 Hz, H-6b), 3.29 (s, 3H, CH_3, Me), 3.27 (dd, 1H, $^3J_{2',1'}$ = 1.9 Hz, $^3J_{2',3'}$ = 2.9 Hz, H-2′), 3.04 (br. s, 1H, 4′-OH), 2.60 (dd, 1H, *J* = 4.7 Hz, *J* = 15.2 Hz, α^{Myr}-CH_2), 2.54 (dd, 1H, *J* = 8.2 Hz, *J* = 15.2 Hz, α^{Myr}-CH_2), 2.47 (d, 2H, *J* = 6.3 Hz, α^{Myr}-CH_2), 2.44-2.37 (m, 2H, α^{Myr}-CH_2), 2.37-2.27 (m, 2H, α-CH_2), 2.25 (t, 2H, *J* = 7.6 Hz, α-CH_2), 2.20 (t, 2H, *J* = 7.6 Hz, α-CH_2), 1.63-1.45 (m, 12H, 3×γ^{Myr}-CH_2, β^{Myr}-CH_2, 2×β^{Lau}-CH_2), 1.33-1.19 (m, 106H, 53×CH_2), 0.89-0.86 (m, 18H, 4×ω^{Myr}-CH_3, 2×ω^{Myr}-CH_3);

^{13}C NMR (151 MHz, $CDCl_3$) δ 174.37, 173.42, 173.37, 171.18, 170.64, 169.72 (6×*C*O), 138.33, 137.92 (2×*C*q, CH_2Ph), 135.56, 135.51 (2×*C*q, OP(O)(OCH_2Ph)$_2$), 128.69, 128.63, 128.54, 128.30, 128.27, 128.14, 128.02, 127.92, 127.61, 127.56 (20×*C*H, CH_2Ph), 92.68 (C-1′), 92.12 (C-1), 78.58 (C-3′), 77.76 (C-2′), 73.75 (C-4, $^2J_{C4,P}$ = 5.6 Hz), 73.45, 73.19 (2×*C*H_2Ph), 72.10 (C-5′), 71.09 (C-3), 70.88 (β^{Myr}-*C*H), 70.60 (C-5, $^3J_{C5,P}$ = 5.6 Hz), 70.54, 69.94 (2×β^{Myr}-*C*H), 69.75 (OP(O)(OCH_2Ph)$_2$, $^2J_{C,P}$ = 5.3 Hz), 69.71 (OP(O)(OCH_2Ph)$_2$, $^2J_{C,P}$ = 5.3 Hz,), 68.40 (C-6), 66.38 (C-4′), 63.37 (C-6′), 59.04 (*C*H_3, Me), 51.59 (C-2), 41.68, 39.43, 39.01 (3×α^{Myr}-*C*H_2), 34.89, 34.59, 34.46, 34.43, 34.23, 34.21 (3×γ^{Myr}-*C*H_2, α^{Myr}-*C*H_2, 2×α^{Lau}-*C*H_2), 31.94, 31.92, 29.72, 29.69, 29.67, 29.64, 29.63, 29.59, 29.57, 29.52, 29.40, 29.37, 29.35, 29.31, 29.26, 29.20, 29.16, 25.28, 25.19, 25.12, 25.05, 25.01, 24.99, 22.69 (56×*C*H_2), 14.10 (4×ω^{Myr}-*C*H_3, 2×ω^{Lau}-*C*H_2);

^{31}P NMR (243 MHz, $CDCl_3$): δ -1.72;

HRMS (+ESI) m/z: calcd for $C_{121}H_{198}NNaO_{22}P$ $[M+Na]^+$ 2071.4035, found 2071.4005.

Synthesis of 26

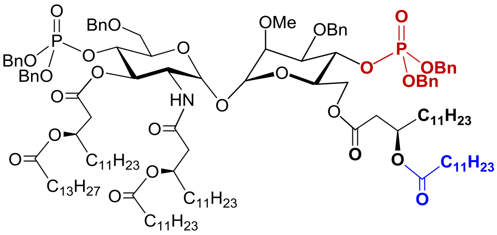

3-*O*-Benzyl-4-*O*-[bis(benzyloxy)phosphoryl]-6-*O*-[(*R*)-3-(dodecanoyloxy)tetradecanoyl]-2-*O*-methyl-α-D-mannopyranosyl-(1↔1)-6-*O*-benzyl-4-*O*-[bis(benzyloxy)phosphoryl]-2-deoxy-2-[(*R*)-3-(dodecanoyloxy)tetradecanoylamino]-3-*O*-[(*R*)-3-(tetradecanoyloxy)tetradecanoyl]-α-D-glucopyranoside (26).

To a stirred solution of **25** (12 mg, 5.7 μmol) and dibenzyl *N,N*-diisopropylphosphoramidite (10 μL, 26 μmol, 90%) in dry DCM (500 μL) a solution of 1*H*-tetrazole (70 μL, 32 μmol, 0.45 M in CH_3CN) was added at r.t. The reaction mixture was stirred for 3h, cooled to -78 °C and a solution of *m*-CPBA (10 mg, 41 μmol, 70 %) in DCM [333 μL of a stock solution prepared from *m*-CPBA (30 mg) and DCM (1 mL)] was added. The reaction mixture was stirred for 20 min at -78 °C, and subsequently quenched by addition of Et_3N (10 μL). The mixture was allowed to warm to r.t., diluted with EtOAc (20 mL) and washed with aq. ciric acid (0.25 M, 20 mL), aq. sat. $NaHCO_3$ (20 mL) and brine (20 mL). The organic layer was dried over Na_2SO_4 and concentrated. The residue was purified by column column chromatography on silica gel (toluene – EtOAc, 10:1 → 5:1) and size exclusion chromatography on Sephadex SX1 (2 cm × 60 cm, toluene – DCM, 3:1) to afford **26** (12 mg, 5.2 μmol, 91%) as colorless syrup. R_f = 0.30 (toluene – EtOAc, 4:1); $[\alpha]_D^{20}$ = +33 (*c* 1.0, $CHCl_3$);

^{1}H-NMR (600 MHz, $CDCl_3$): δ 7.41-7.39 (m, 2H, CH_2Ph), 7.34-7.19 (m, 28H, CH_2Ph), 6.64 (d, 1H, $^2J_{NH,2}$ = 8.5 Hz, N*H*), 5.26 (dd, 1H, $^3J_{3,4}$ = 9.0 Hz, $^3J_{3,2}$ = 11.0 Hz, H-3), 5.20-5.15 (m, 2H, 2×β^{Myr}-C*H*), 5.16 (d, 1H, $^3J_{1,2}$ = 3.7 Hz, H-1), 5.11 (d, 1H, $^3J_{1',2'}$ = 1.9 Hz, H-1′), 5.10-5.06 (m, 1H, β^{Myr}-C*H*), 5.01-4.90 (m, 8H, 2×OP(O)(OCH_2Ph)$_2$), 4.69 (AB, 1H, ^2J = 11.5 Hz, CH_2Ph), 4.67-4.63 (m, 1H, H-4′), 4.65 (AB, 1H, ^2J = 11.6 Hz, CH_2Ph), 4.47 (AB, 1H, ^2J = 11.8 Hz, CH_2Ph), 4.42 (t, 1H, $^3J_{4,3}$ = $^3J_{4,5}$ = $^3J_{4,P}$ = 9.3 Hz, H-4), 4.39 (AB, 1H, ^2J = 11.8 Hz, CH_2Ph), 4.34-4.30 (m, 2H, H-2, H-6a′), 4.26 (dd, 1H, $^3J_{6b',5}$ = 5.5 Hz, $^2J_{6b',6a}$ = 12.2 Hz, H-6b′), 4.22 (dd, 1H, $^3J_{3',2'}$ = 3.2 Hz, $^3J_{3',4'}$ = 9.5 Hz, H-3′), 4.01 (ddd, 1H, $^3J_{5',6a'}$ = 2.0 Hz, $^3J_{5',6b'}$ = 5.5 Hz, $^3J_{5',4'}$ = 9.7 Hz, H-5′), 3.74 (dd, 1H, $^3J_{6a,6b}$ = 1.7 Hz, $^2J_{6a,6b}$ = 10.7 Hz, H-6a), 3.72-3.69 (m, 1H, H-5), 3.57 (dd, 1H, $^3J_{6b,5}$ = 5.9 Hz, $^2J_{6b,6a}$ = 10.8 Hz, H-6b), 3.25 (s, 3H, CH_3, Me), 3.23 (t, 1H, *J* = 2.6 Hz, H-2′), 2.59 (dd, 1H, *J* = 7.6 Hz, *J* = 16.0 Hz, α^{Myr}-CH_2), 2.56-2.49 (m, 3H, α^{Myr}-CH_2), 2.45 (d, 2H, *J* = 6.1 Hz, α^{Myr}-CH_2), 2.29-2.20 (m, 4H, 2×α-CH_2), 2.17 (t, 2H, *J* = 7.6 Hz, α^{Myr}-CH_2), 1.59-1.47 (m, 12H, 3×γ^{Myr}-CH_2, β^{Myr}-CH_2, 2×β^{Lau}-CH_2), 1.33-1.16 (m, 106H, 53×CH_2), 0.89-0.86 (m, 18H, 4×ω^{Myr}-CH_3, 2×ω^{Lau}-CH_3);

^{13}C-NMR (151 MHz, $CDCl_3$): δ 173.78, 173.27, 173.02, 171.05, 170.19, 170.02 (6×*C*O), 137.93 (2×*C*q, CH_2Ph), 135.81 (*C*q, $^3J_{C,P}$ = 6.9 Hz, OP(O)(OCH_2Ph)$_2$), 135.76 (*C*q, $^3J_{C,P}$ = 7.2 Hz, OP(O)(OCH_2Ph)$_2$), 135.58, 135.53 (2×*C*q, OP(O)(OCH_2Ph)$_2$), 128.68, 128.67, 128.62, 128.48, 128.45, 128.34, 128.33, 128.32, 128.29, 128.14, 128.00, 127.92, 127.88, 127.67, 127.59, 127.54 (30×*C*H, CH_2Ph), 92.48 (C-1), 92.23 (C-1′), 77.27 (C-2′), 76.56 (C-3′), 73.81, 73.74 (C-4, C-4′, $^2J_{C,P}$ = 6.0 Hz, $^2J_{C,P}$ = 6.1 Hz), 73.42, 72.48 (2×*C*H_2Ph), 71.02 (C-3), 70.67, 70.65 (C-5, β^{Myr}-*C*H), 70.26 (C-5′, $^3J_{C3,P}$ = 4.2 Hz, C-5′), 70.08, 69.81 (2×β^{Myr}-*C*H), 69.76 (OP(O)(O*C*H_2Ph)$_2$, $^2J_{C,P}$ = 5.4 Hz), 69.70 (OP(O)(O*C*H_2Ph)$_2$, $^2J_{C,P}$ = 5.5 Hz), 69.41 (OP(O)(O*C*H_2Ph)$_2$, $^2J_{C,P}$ = 5.4 Hz), 69.23 (OP(O)(O*C*H_2Ph)$_2$ $^2J_{C,P}$ = 5.3 Hz), 68.41 (C-6), 62.77 (C-6′), 58.96 (*C*H_3, Me), 51.28 (C-2), 41.37, 38.84, 38.66 (3×α^{Myr}-*C*H_2), 34.74, 34.47, 34.46, 34.42, 34.24, 33.94 (3×γ^{Myr}-*C*H_2, α^{Myr}-*C*H_2, 2×α^{Lau}-*C*H_2), 31.93, 31.92, 29.73, 29.71, 29.69, 29.66, 29.64, 29.62, 29.61, 29.56, 29.52, 29.46, 29.43, 29.40, 29.38, 29.36, 29.35, 29.33, 29.32, 29.26, 29.19, 25.31, 25.17, 25.07, 25.04, 25.00, 22.68 (56×*C*H_2), 14.10 (4×ω^{Myr}-*C*H_3, 2×ω^{Lau}-*C*H_3);

^{31}P-NMR (243 MHz, $CDCl_3$): δ -1.66, -2.38;

HRMS (+ESI) m/z: calcd for $C_{135}H_{211}NNaO_{25}P_2$ $[M+Na]^+$ 2331.4638, found 2331.4672.

Synthesis of 27

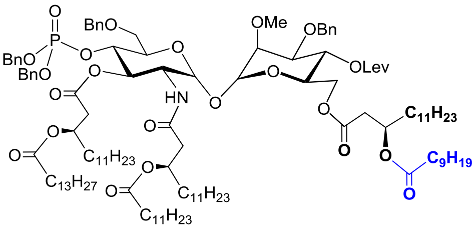

3-*O*-Benzyl-6-*O*-[(*R*)-3-(decanoyloxy)tetradecanoyl]-4-*O*-levulinoyl-2-*O*-methyl-α-D-mannopyranosyl-(1↔1)-6-*O*-benzyl-4-*O*-[bis(benzyloxy)phosphoryl]-2-deoxy-2-[(*R*)-3-(dodecanoyloxy)-tetradecanoylamino]-3-*O*-[(*R*)-3-(tetradecanoyloxy)tetradecanoyl]-α-D-glucopyranoside (27).

To stirred solution of **23** (27 mg, 15.5 µmol) and DMAP (0.4 mg, 3 µmol) in dry DCM (500 µL) a solution of (*R*)-3-(decanoyloxy)tetradecanoic acid **16** (10 mg, 25 µmol) in dry DCM (1 mL) and a solution of DIC (7 mg, 55 µmol) in dry DCM (1 mL) were gradually added over a period of 5h at 0 °C. The mixture was additionally stirred for 1 h, diluted with EtOAc (20 mL) and washed with aq. citric acid (0.25 M, 20 mL), aq. sat. $NaHCO_3$ (20 mL) and brine (20 mL). The organic layer was dried over Na_2SO_4, filtered and concentrated. The residue was purified by HPLC (column A: hexane – EtOAc, 3:1 → 2:1) to afford **27** (24 mg, 11 µmol, 73%) as a colourless syrup. R_f = 0.59 (toluene – EtOAc, 4:1); $[\alpha]_D^{20}$ = +32 (*c* 0.6, $CHCl_3$);

^{1}H-NMR (600 MHz, $CDCl_3$) δ 7.39-7.21 (m, 20H, CH_2Ph), 6.39 (d, 1H, $^3J_{NH,2}$ = 8.0 Hz, N*H*), 5.27-5.16 (m, 5H, H-1, H-3, H-4′, 2×β^{Myr}-C*H*), 5.12 (d, 1H, $^3J_{1',2'}$ = 1.9 Hz, H-1′), 5.08-5.04 (m, 1H, β^{Myr}-C*H*), 4.97 (d, 2H, *J* = 8.2 Hz, OP(O)(OCH_2Ph)$_2$), 4.94 (d, 2H, *J* = 8.2 Hz, OP(O)(OCH_2Ph)$_2$), 4.70 (AB, 1H, ^2J = 11.9 Hz, CH_2Ph), 4.65 (AB, 1H, ^2J = 11.9 Hz, CH_2Ph), 4.48 (AB, 1H, ^2J = 11.8 Hz, CH_2Ph), 4.46-4.41 (m, 1H, H-4), 4.40 (AB, 1H, ^2J = 11.9 Hz, CH_2Ph), 4.27-4.22 (m, 2H, H-2, H-6a′), 4.01-3.98 (m, 2H, H-3′, H-6b′), 3.81 (ddd, 1H, *J* = 1.9 Hz, *J* = 5.6 Hz, *J* = 10.0 Hz, H-5′), 3.75-3.71 (m, 2H, H-5, H-6a), 3.58 (dd, 1H, *J* = 5.8 Hz, *J* = 11.0 Hz, H-6b), 3.30 (s, 3H, CH_3, Me), 3.28 (t, 1H, *J* = 2.6 Hz, H-2′), 2.76-2.65 (m, 2H, CH_2, Lev), 2.61-2.47 (m, 6H, 2×α^{Myr}-CH_2, CH_2, Lev), 2.43 (dd, 1H, *J* = 7.3 Hz, *J* = 15.4 Hz, α^{Myr}-CH_2), 2.37 (dd, 1H, *J* = 5.0 Hz, *J* = 15.4 Hz, α^{Myr}-CH_2), 2.35-2.27 (m, 2H, α-CH_2), 2.24 (t, 2H, *J* = 7.6 Hz, α-CH_2), 2.19 (t, 1H, *J* = 7.6 Hz, α-CH_2), 2.15 (s, 3H, CH_3, Lev), 1.62-1.45 (m, 12H, 3×γ^{Myr}-CH_2, β^{Myr}-CH_2, β^{Lau}-CH_2, β^{Cap}-CH_2), 1.33-1.20 (m, 102H, 51×CH_2), 0.89-0.86 (m, 18H, 4×ω^{Myr}-CH_3, ω^{Lau}-CH_3, ω^{Cap}-CH_3);

^{13}C-NMR (151 MHz, $CDCl_3$) δ 205.95 (*C*O, Lev), 173.82, 173.36, 173.04, 171.64, 171.34, 170.30, 169.69 (7×*C*O), 138.10, 137.89 (2×*C*q, CH_2Ph), 135.56, 135.51 (2x*C*q, OP(O)(OCH_2Ph)$_2$), 128.70, 128.63, 128.50, 128.31, 128.15, 128.12, 128.02, 127.87, 127.62, 127.57 (20×*C*H, CH_2Ph), 92.98 (C-1′), 92.39 (C-1), 77.66 (C-2′), 76.23 (C-3′), 73.68 (C-4, $^2J_{C4,P}$ = 6.0 Hz), 73.47, 72.76 (2×*C*H_2Ph), 71.07 (C-3), 70.64, 70.61 (C-5, β^{Myr}-*C*H), 70.10, 69.86 (2×β^{Myr}-*C*H), 69.76, 69.74, 69.73, 69.71 (OP(O)(O*C*H_2Ph)$_2$), 68.35 (C-6), 67.70 (C-4′), 62.40 (C-6′), 59.23 (*C*H_3, Me), 51.67 (C-2), 41.57, 38.95, 38.74 (3×α^{Myr}-*C*H_2), 37.90 (*C*H_2, Lev), 34.79, 34.53, 34.48, 34.44, 34.28, 34.03 (3×γ^{Myr}-*C*H_2, α^{Myr}-*C*H_2, α^{Lau}-*C*H_2, α^{Cap}-*C*H_2), 31.94, 31.93, 31.88, 29.75, 29.73, 29.70, 29.67, 29.64, 29.62, 29.57, 29.54, 29.47, 29.43, 29.40, 29.37, 29.35, 29.32, 29.29, 29.19, 29.18, 27.94, 25.40, 25.19, 25.10, 25.09, 25.01, 22.69, 22.67 (55×*C*H_2, *C*H_3, Lev), 14.10 (4×ω^{Myr}-*C*H_3, ω^{Lau}-*C*H_3, ω^{Cap}-*C*H_3);

^{31}P-NMR (243 MHz, $CDCl_3$) δ -1.70;

HRMS (+ESI) m/z: calcd for $C_{124}H_{200}NNaO_{24}P$ $[M+Na]^+$ 2141.4090, found 2141.3982.

Synthesis of 28

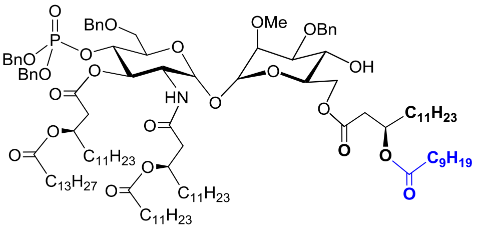

3-*O*-Benzyl-6-*O*-[(*R*)-3-(decanoyloxy)tetradecanoyl]-2-*O*-methyl-α-D-mannopyranosyl-(1↔1)-6-*O*-benzyl-4-*O*-[bis(benzyloxy)phosphoryl]-2-deoxy-2-[(*R*)-3-(dodecanoyloxy)tetradecanoylamino]-3-*O*-[(*R*)-3-(tetradecanoyloxy)tetradecanoyl]-α-D-glucopyranoside (28).

To a stirred solution of **27** (22 mg, 10 µmol) in pyridine – AcOH (5:4, 800 µL) hydrazine monohydrate (20 µL, 64% hydrazine) was added. The mixture was stirred for 2.5 h at r.t, then acetone (2 mL) was added and the mixture was stirred for 5 min. The solution was diluted with EtOAc (20 mL) and washed with aq. HCl (2 M, 20 mL), aq. sat. $NaHCO_3$ (20 mL) and brine (20 mL). The organic layer was dried over Na_2SO_4, filtered and concentrated. The residue was purified by HPLC (column A: hexane – EtOAc, 4:1 → 3:1) to afford **28** (15 mg, 7.4 µmol, 72%) as a colourless syrup. R_f = 0.52 (toluene – EtOAc, 3:1); $[\alpha]_D^{20}$ = +32 (*c* 1.0, $CHCl_3$);

^{1}H-NMR (600 MHz, $CDCl_3$): δ 7.44-7.43 (m, 2H, CH_2Ph), 7.37-7.21 (m, 18H, CH_2Ph), 6.34 (d, 1H, $^3J_{NH,2}$ = 8.2 Hz, N*H*), 5.24-5.19 (m, 3H, H-1, H-3, β^{Myr}-C*H*), 5.16-5.12 (m, 2H, H-1′, β^{Myr}-C*H*), 5.08-5.04 (m, 1H, β^{Myr}-C*H*), 4.96 (d, 2H, *J* = 8.1 Hz, OP(O)(OCH_2Ph)$_2$), 4.94 (d, 2H, *J* = 8.2 Hz, OP(O)(OCH_2Ph)$_2$), 4.85 (AB, 1H, ^2J = 11.6 Hz, CH_2Ph), 4.74 (AB, 1H, ^2J = 11.7 Hz, CH_2Ph), 4.48 (AB, 1H, ^2J = 11.9 Hz, CH_2Ph), 4.44-4.39 (m, 1H, H-4), 4.40 (AB, 1H, ^2J = 12.0 Hz, CH_2Ph), 4.35 (dd, 1H, $^3J_{6a',5'}$ = 5.0 Hz, $^2J_{6a',6b'}$ = 12.1 Hz, H-6a′), 4.26 (ddd, 1H, $^3J_{2,1}$ = 3.9 Hz, $^3J_{2,NH}$ = 8.3 Hz, $^3J_{2,3}$ = 10.8 Hz, H-2), 4.19 (dd, 1H, $^3J_{6b',5'}$ = 1.7 Hz, $^2J_{6b',6a'}$ = 12.1 Hz, H-6b′), 3.93 (t, 1H, $^3J_{4',3'}$ = $^3J_{4',5'}$ = 9.7 Hz, H-4′), 3.85 (dd, 1H, $^3J_{3',2'}$ = 3.1 Hz, $^3J_{3',4'}$ = 9.7 Hz, H-3′), 3.75-3.71 (m, 2H, H-5, H-6a), 3.66 (ddd, 1H, $^3J_{5',6b'}$ = 1.8 Hz, $^3J_{5',6a'}$ = 4.9 Hz, $^3J_{5',4'}$ = 9.8 Hz, H-5′), 3.58 (dd, 1H, *J* = 5.9 Hz, *J* = 10.9 Hz, H-6b), 3.29 (s, 3H, CH_3, Me), 3.27-3.26 (m, 1H, H-2′), 3.04 (br. s, 1H, 4′-OH), 2.60 (dd, 1H, *J* = 4.8 Hz, *J* = 15.2 Hz, α^{Myr}-CH_2), 2.54 (dd, 1H, *J* = 8.2 Hz, *J* = 15.2 Hz, α^{Myr}-CH_2), 2.48 (d, 2H, *J* = 6.2 Hz, α^{Myr}-CH_2), 2.44-2.38 (m, 2H, α^{Myr}-CH_2), 2.38-2.27 (m, 2H, α-CH_2), 2.25 (t, 2H, *J* = 7.6 Hz, α-CH_2), 2.20 (t, 2H, *J* = 7.6 Hz, α-CH_2), 1.63-1.45 (m, 12H, 3×γ^{Myr}-CH_2, β^{Myr}-CH_2, β^{Lau}-CH_2, β^{Cap}-CH_2), 1.32-1.20 (m, 102H, 51×CH_2), 0.89-0.86 (m, 18H, 4×ω^{Myr}-CH_3, ω^{Lau}-CH_3, ω^{Cap}-CH_3);

^{13}C NMR (151 MHz, $CDCl_3$): δ 174.37, 173.40, 173.36, 171.17, 170.63, 169.71 (6×*C*O), 138.33, 137.91 (2×*C*q, CH_2Ph), 135.55, 135.51 (2×*C*q, OP(O)(OCH_2Ph)$_2$), 128.69, 128.62, 128.53, 128.30, 128.26, 128.13, 128.02, 127.91, 127.60, 127.55 (20×*C*H, CH_2Ph), 92.68 (C-1′), 92.11 (C-1), 78.58 (C-3′), 77.76 (C-2′), 73.75 (C-4, $^2J_{C,P}$ = 5.6 Hz), 73.45, 73.19 (2×*C*H_2Ph), 72.10 (C-5′), 71.09 (C-3), 70.87 (β^{Myr}-*C*H), 70.60 (C-5, $^3J_{C5,P}$ = 5.3 Hz), 70.54 (β^{Myr}-*C*H), 69.93 (β^{Myr}-*C*H), 69.74 (OP(O)(O*C*H_2Ph)$_2$, $^2J_{C,P}$ = 5.4 Hz), 69.71 (OP(O)(O*C*H_2Ph)$_2$, $^2J_{C,P}$ = 5.4 Hz), 68.39 (C-6), 66.38 (C-4′), 63.37 (C-6′), 59.03 (*C*H_3, Me), 51.59 (C-2), 41.68, 39.42, 39.00 (3×α^{Myr}-*C*H_2), 34.89, 34.58, 34.44, 34.42, 34.23, 34.20 (3×γ^{Myr}-*C*H_2, α^{Myr}-*C*H_2, α^{Lau}-*C*H_2, α^{Cap}-*C*H_2) 31.93, 31.92, 31.87, 29.72, 29.69, 29.66, 29.62, 29.58, 29.56, 29.51, 29.46, 29.41, 29.39, 29.36, 29.35, 29.29, 29.25, 29.20, 29.14, 25.27, 25.19, 25.12, 25.05, 25.00, 24.98, 22.68, 22.66 (54×*C*H_2), 14.09 (4×ω^{Myr}-*C*H_3, ω^{Lau}-*C*H_3, ω^{Cap}-*C*H_3);

^{31}P-NMR (243 MHz, $CDCl_3$): δ -1.72;

HRMS (+ESI) m/z: calcd for $C_{119}H_{194}NNaO_{22}P$ $[M+Na]^+$ 2043.3722, found 2043.3787.

Synthesis of 29

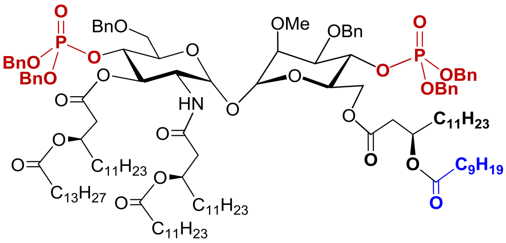

3-*O*-Benzyl-4-*O*-[bis(benzyloxy)phosphoryl]-6-*O*-[(*R*)-3-(decanoyloxy)tetradecanoyl]-2-*O*-methyl-α-D-mannopyranosyl-(1↔1)-6-*O*-benzyl-4-*O*-[bis(benzyloxy)phosphoryl]-2-deoxy-2-[(*R*)-3-(dodecanoyloxy)tetradecanoylamino]-3-*O*-[(*R*)-3-(tetradecanoyloxy)tetradecanoyl]-α-D-glucopyranoside (29).

To a stirred solution of **28** (14 mg, 7 µmol) and dibenzyl *N,N*-diisopropylphosphoramidite (6 µL, 16 µmol, 90%) in dry DCM (500 µL) a solution of 1*H*-tetrazole (78 µL, 35 µmol, 0.45 M in CH_3CN) was added at r.t. The mixture was stirred for 1 h, cooled to -78 °C and a solution of *m*-CPBA (10 mg, 41 µmol, 70%) in DCM [370 µL of a stock solution prepared from *m*-CPBA (27 mg) and DCM (1 mL)] was added. After stirring for 1 h at -78 °C, the reaction was quenched by addition of Et_3N (15 µL). reaction mixture was allowed to warm to r.t., diluted with EtOAc (20 mL) and washed with aq. citric acid (0.25 M, 20 mL), aq. sat. $NaHCO_3$ (20 mL) and brine (20 mL). The organic layer was dried over Na_2SO_4, filtered and concentrated. The residue was purified by column chromatography on silica gel (toluene – EtOAc, 10:1 → 5:1) and size exclusion chromatography on Sephadex SX1 (2 cm × 60 cm, toluene – DCM, 3:1) to afford **29** (15 mg, 6.4 µmol, 90%) as a colorless syrup. R_f = 0.26 (toluene – EtOAc, 4 : 1); $[\alpha]_D^{20}$ = +37 (1.1, $CHCl_3$);

^{1}H-NMR (600 MHz, $CDCl_3$): δ 7.41-7.40 (m, 2H, CH_2Ph), 7.34-7.19 (m, 28H, CH_2Ph), 6.64 (d, 1H, $^3J_{NH,2}$ = 8.5 Hz, N*H*), 5.26 (dd, 1H, ^3J = 9.1 Hz, ^3J = 11.0 Hz, H-3), 5.20-5.15 (m, 2H, 2×β^{Myr}-C*H*), 5.16 (d, 1H, $^3J_{1,2}$ = 3.7 Hz, H-1), 5.11 (d, 1H, $^3J_{1',2'}$ = 1.8 Hz, H-1′), 5.10-5.06 (m, 1H, β^{Myr}-C*H*), 5.01-4.90 (m, 8H, 2×OP(O)(OCH_2Ph)$_2$), 4.69 (AB, 1H, ^2J = 11.5 Hz, CH_2Ph), 4.67-4.63 (m, 2H, H-4′, CH_2Ph), 4.47 (AB, 1H, ^2J = 11.9 Hz, CH_2Ph), 4.42 (d, 1H, $^3J_{4,3}$ = $^3J_{4,5}$ = $^3J_{4,P}$ = 9.3 Hz, H-4), 4.39 (AB, 1H, ^2J = 11.8 Hz, CH_2Ph), 4.34-4.30 (m, 2H, H-2, H-6a′), 4.26 (dd, 1H, J = 5.5 Hz, J = 12.2 Hz, H-6b′), 4.22 (dd, 1H, $^3J_{3',2'}$ = 3.1 Hz, $^3J_{3',4'}$ = 9.5 Hz, H-3′), 4.02-3.99 (m, 1H, H-5′), 3.75-3.73 (m, 1H, H-6a), 3.72-3.69 (m, 1H, H-5), 3.57 (dd, 1H, J = 5.9 Hz, J = 10.8 Hz, H-6b), 3.25 (s, 3H, CH_3, Me), 3.23 (t, J = 2.6 Hz, H-2′), 2.59 (dd, 1H, J = 7.5 Hz, J = 16.0 Hz, α^{Myr}-CH_2), 2.56-2.47 (m, 3H, α^{Myr}-CH_2), 2.45 (d, 2H, J = 6.1 Hz, α^{Myr}-CH_2), 2.29-2.20 (m, 4H, 2×α-CH_2), 2.17 (t, 2H, J = 7.6 Hz, α-CH_2), 1.59-1.47 (m, 12H, 3×γ^{Myr}-CH_2, β^{Myr}-CH_2, β^{Lau}-CH_2, β^{Cap}-CH_2), 1.32-1.16 (m, 102H, 51×CH_2), 0.89-0.86 (m, 18H, 4×ω^{Myr}-CH_3, ω^{Lau}-CH_3, ω^{Cap}-CH_3);

^{13}C-NMR (151 MHz, $CDCl_3$): δ 173.78, 173.26, 173.02, 171.05, 170.18, 170.02 (6×*C*O), 137.93 (2×*C*q, CH_2Ph), 135.80 (*C*q, $^3J_{C,P}$ = 6.7 Hz, OP(O)(OCH_2Ph)$_2$), 135.76 (*C*q, $^3J_{C,P}$ = 7.3 Hz, OP(O)(OCH_2Ph)$_2$), 135.57, 135.53 (2×*C*q, OP(O)(OCH_2Ph)$_2$), 128.68, 128.66, 128.62, 128.48, 128.44, 128.33, 128.31, 128.29, 128.14, 128.00, 127.91, 127.88, 127.67, 127.59, 127.53 (30×*C*H, CH_2Ph), 92.48 (C-1), 92.22 (C-1′), 77.27 (C-2′), 76.56 (C-3′), 73.81 (C-4, $^2J_{C,P}$ = 5.9 Hz), 73.73 (C-4′, $^2J_{C,P}$ = 6.2 Hz), 73.42, 72.48 (2x*C*H_2Ph), 71.02 (C-3), 70.67, 70.65 (C-5, β^{Myr}-*C*H), 70.26 (C-5′, $^3J_{C5,P}$ = 4.0 Hz), 70.08, 69.80 (2×β^{Myr}-*C*H), 69.76 (OP(O)(O*C*H_2Ph)$_2$, $^2J_{C,P}$ = 5.4 Hz), 69.70 (OP(O)(O*C*H_2Ph)$_2$, $^2J_{C,P}$ = 5.5 Hz), 69.41 (OP(O)(O*C*H_2Ph)$_2$, $^2J_{C,P}$ = 5.3 Hz), 69.23 (OP(O)(O*C*H_2Ph)$_2$, *C*H_2, $^2J_{C,P}$ = 5.4 Hz), 68.41 (C-6), 62.76 (C-6′), 58.96 (*C*H_3, Me), 51.27 (C-2), 41.36, 38.84, 38.65 (3×α^{Myr}-*C*H_2), 34.73, 34.47, 34.46 34.42, 34.24, 33.94 (3×γ^{Myr}-*C*H_2, α^{Myr}-*C*H_2, α^{Lau}-*C*H_2, α^{Cap}-*C*H_2), 31.93, 31.92, 31.87, 29.72, 29.70, 29.69, 29.66, 29.61, 29.56, 29.46, 29.42, 29.39, 29.37, 29.36, 29.33, 29.31, 29.28, 29.26, 29.18, 29.17, 25.31, 25.17, 25.06, 25.04, 24.99, 22.68, 22.66 (54×*C*H_2), 14.10 (4×ω^{Myr}-*C*H_3, ω^{Lau}-*C*H_3, ω^{Cap}-*C*H_3);

^{31}P-NMR (243 MHz, $CDCl_3$): δ -1.67, -2.39;

HRMS (+ESI) m/z: calcd for $C_{133}H_{207}NNaO_{25}P_2$ $[M+Na]^+$ 2303.4325, found 2303.4351.

Synthesis of 30

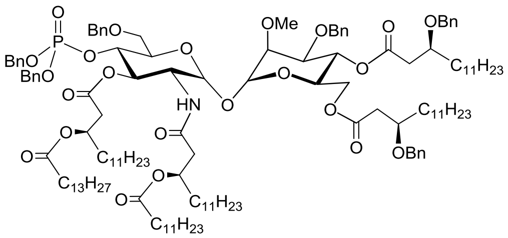

3-*O*-Benzyl-4,6-di-*O*-[(*R*)-3-(benzyloxy)tetradecanoyl]-2-*O*-methyl-α-D-mannopyranosyl-(1↔1)-6-*O*-benzyl-4-*O*-[bis(benzyloxy)phosphoryl]-2-deoxy-2-[(*R*)-3-(dodecanoyloxy)tetradecanoylamino]-3-*O*-[(*R*)-3-(tetradecanoyloxy)tetradecanoyl]-α-D-glucopyranoside (30).

To a stirred solution of **22** (19 mg, 12 µmol) and DMAP (0.1 mg, 0.8 µmol) in dry DCM (2 mL) a solution of **17** (15 mg, 45 µmol) in dry DCM (1 mL) and a solution of DIC (9 mg, 69 µmol) in dry DCM (1 mL) were added gradually over a period of 4 h at 0 °C. The mixture was additionally stirred for 1 h, diluted with DCM (30 mL) and washed with aq. citric acid (0.25 M, 10 mL), aq. sat. $NaHCO_3$ (10 mL) and brine (10 mL). The organic layer was dried over Na_2SO_4, filtered and concentrated. The residue was purified by column chromatography on silica gel (hexane – EtOAc, 2:1) and by HPLC (column B: hexane – EtOAc, 4:1 → 3:1) to afford **3** (22 mg, 9.7 µmol, 81%) as a colorless syrup. R_f = 0.24 (hexane - EtOAc, 3 : 1); $[\alpha]_D^{20}$ =+34 (*c* 0.60, $CHCl_3$);

^{1}H-NMR (600 MHz, $CDCl_3$): δ 7.35-7.18 (m, 30H, CH_2Ph), 6.38 (d, 1H, $^3J_{NH,2}$ = 8.1 Hz, N*H*), 5.30 (t, 1H, $^3J_{4',3'}$ = $^3J_{4',5'}$ = 9.8 Hz, H-4′), 5.23-5.16 (m, 3H, H-1, H-3, β-C*H*), 5.11 (d, 1H, $^3J_{1',2'}$ = 2.0 Hz, H-1′), 5.08-5.04 (m, 1H, β-C*H*), 4.97 (d, 2H, *J* = 8.1 Hz, OP(O)(OCH_2Ph)$_2$), 4.94 (d, 2H, *J* = 8.2 Hz, OP(O)(OCH_2Ph)$_2$), 4.63 (AB, 1H, ^2J = 12.1 Hz, CH_2Ph), 4.58 (AB, 1H, ^2J = 12.1 Hz, CH_2Ph), 4.56 (AB, 1H, ^2J = 2.8 Hz, CH_2Ph), 4.54 (AB, 1H, ^2J = 2.8 Hz, CH_2Ph), 4.48-4.38 (m, 5H, H-4, 2xCH_2Ph), 4.27-4.24 (m, 1H, H-2), 4.16 (dd, 1H $^3J_{6a',5'}$ = 6.0 Hz, $^2J_{6a',6b'}$ = 12.3 Hz, H-6a′), 4.06 (dd, 1H, $^3J_{6b',5'}$ = 2.0 Hz, $^2J_{6b',6a'}$ = 12.1 Hz, H-6b′), 4.00 (dd, 1H, $^3J_{3',2'}$ = 3.0 Hz, $^3J_{3',4'}$ = 9.6 Hz, H-3′), 3.90-3.79 (m, 3H, H-5′, 2×β-C*H*), 3.74-3.69 (m, 2H, H-5, H-6a), 3.58 (dd, 1H, *J* = 5.4 Hz, *J* = 10.6 Hz, H-6b), 3.27 (s, 3H, CH_3, Me), 3.23 (m, 1H, H-2′), 2.65-2.17 (m, 12H, 4×α^{Myr}-CH_2, 2×α^{Lau}-CH_2), 1.61-1.48 (m, 12H, 4×γ^{Myr}-CH_2, β^{Myr}-CH_2, β^{Lau}-CH_2), 1.42-1.11 (m, 108H, 54×CH_2), 0.89-0.86 (m, 18H, 5×ω^{Myr}-CH_3, 5×ω^{Lau}-CH_3);

^{13}C-NMR (151 MHz, $CDCl_3$): δ 173.79, 173.32, 171.49, 171.38, 170.60, 169.63 (6×*C*O), 138.85, 138.69, 138.07, 137.90 (4×*C*q, CH_2Ph), 135.58, 135.53 (2×*C*q, OP(O)(OCH_2Ph)$_2$), 128.70, 128.64, 128.49, 128.32, 128.21, 128.12, 128.02, 127.86, 127.75, 127.69, 127.63, 127.57, 127.41, 127.35 (30×*C*H, CH_2Ph), 92.80 (C-1′), 92.39 (C-1), 77.65 (C-2′), 76.04 (C-3′), 75.95, 75.63 (2×β-*C*H), 73.67 (C-4, $^2J_{C4,P}$ = 6.9 Hz), 73.48, 72.69, 71.59, 71.41 (4×*C*H_2, *C*H_2PH), 71.14 (C-5), 70.62, 70.58, 70.24, 69.82 (C-3, C-5′, 2×β-*C*H), 69.82, 69.77, 69.75, 69.74 (2×*C*H_2, OP(O)(O*C*H_2Ph)$_2$), 68.35 (C-6) 67.68 (C-4′), 62.54 (C-6′), 59.06 (*C*H_3, Me), 51.65 (C-2), 41.63, 39.82, 39.56, 38.90 (4×α^{Myr}-*C*H_2), 34.82, 34.58, 34.55, 34.45, 34.27, 31.94, 29.71, 29.69, 29.67, 29.64, 29.57, 29.54, 29.44, 29.38, 29.34, 29.30, 29.20, 25.38, 25.23, 25.18, 25.13, 25.01, 22.69 (62×*C*H_2, fatty acid), 14.09 (5×ω^{Myr}-*C*H_3, 5×ω^{Lau}-*C*H_3);

^{31}P-NMR (243 MHz, $CDCl_3$): δ -1.72;

HRMS (+ESI) m/z: calcd for $C_{137}H_{214}NNaO_{23}P$ $[M+Na]^+$ 2295.5236, found 2295.5276.

Synthesis of 4

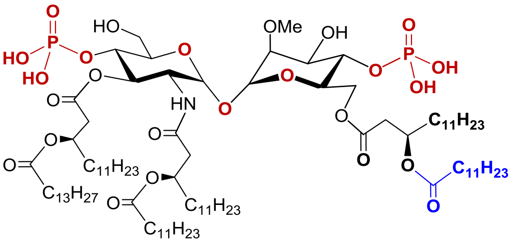

6-*O*-[(*R*)-3-(Dodecanoyloxy)tetradecanoyl]-2-*O*-methyl-4-*O*-phosphoryl-α-D-mannopyranosyl-(1↔1)-2-deoxy-2-[(*R*)-3-(dodecanoyloxy)tetradecanoylamino]-4-*O*-phosphoryl-3-*O*-[(*R*)-3-(tetradecanoyloxy)tetradecanoyl]-α-D-glucopyranoside (4).

Palladium black (3 mg, 28 µmol) was added to a solution of **26** (5.3 mg, 2.3 µmol) in toluene – MeOH (2 mL, 1:1), the mixture was placed under hydrogen atmosphere and stirred for 10 h at r.t. After partial debenzylation, the hydrophiliciy of the intermediates increased and the solubility of the intermediate products droped. To dissolve an increasingly formed precipitate, the mixture was diluted with MeOH (2 mL), additional Pd black (3 mg, 28 µmol) was added and the mixture was placed under hydrogen atmosphere and stirred for 8 h at r.t. The mixture was diluted with DCM – EtOH (1:1, 5 mL) and the solids were removed by filtration over regenerated cellulose filter (45 µm). The filtrate was concentrated to afford **4** (4.0 mg, 2.26 µmol, 98%) as a white solid; R_f = 0.35 ($CHCl_3$ – MeOH – H_2O, 50:28:6).

For biological studies, the residue was purified by size exclusion chrmoatography on BioBeads SX1 (BioRad, column 800x1.5 mm, elution with toluene – DCM, 3:1). Appropriate fractions were collected and concentrated, the residue was dissolved in DMSO (2 mL), filtered through a regenerated cellulose filter (45 µm) and the filtrate was lyophilized to afford **4** (2.8 mg, 1.6 µmol, 69%).

^{1}H-NMR (600 MHz, $CDCl_3$ – MeOD, 3 : 1): δ 5.24-5.11 (m, 6H, H-1, H-1′, H-3, 3×β^{Myr}-C*H*), 4.32-4.22 (m, 5H, H-2, H-4, H-6a′, H-6b′), 4.16-4.11 (m, 1H, H-3′), 3.97-3.91 (m, 1H, H-6a), 3.82-3.66 (m, 3H, H-5, H-5′, H-6b), 3.51 (s, 1H, H-2′), 3.48 (s, 3H, C*H*$_3$, Me), 2.69-2.60 (m, 4H, 2×α^{Myr}-C*H*$_2$), 2.47-2.38 (m, 2H, α^{Myr}-C*H*$_2$), 2.32-2.25 (m, 6H, α^{Myr}-C*H*$_2$, 2×α^{Lau}-C*H*$_2$), 1.66-1.50 (m, 12H, β^{Myr}-C*H*$_2$, 2×β^{Lau}-C*H2,* 3×γ^{Myr}-C*H*$_2$), 1.38-1.16 (m, 106H, 53×C*H*$_2$), 0.88 (t, 18H, *J* = 7.0 Hz, 4×ω^{Myr}-C*H*$_3$, 2×ω^{Lau}-C*H*$_3$);

^{31}P NMR (243 MHz, MeOD) δ 0.46 (s, 1P), 0.32 (s, 1P).

MS (-MALDI) m/z: calcd for $C_{93}H_{174}NO_{25}P_2$ $[M-H]^-$ 1767.187, found 1767.090.

Synthesis of 5

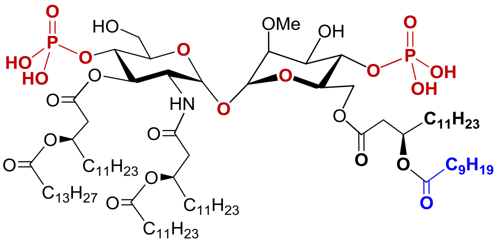

6-*O*-[(*R*)-3-(Decanoyloxy)tetradecanoyl]-2-*O*-methyl-4-*O*-phosphoryl-α-D-mannopyranosyl-(1↔1)-2-deoxy-2-[(*R*)-3-(dodecanoyloxy)tetradecanoylamino]-4-*O*-phosphoryl-3-*O*-[(*R*)-3-(tetradecanoyloxy)tetradecanoyl]-α-D-glucopyranoside (5).

Palladium black (20 mg) was added to a solution of **29** (40 mg, 17.5 µmol) in toluene – MeOH (8 mL, 1:1), the mixture was placed under hydrogen atmosphere and stirred for 12 h at r.t. After partial debenzylation, the hydrophiliciy of the intermediate products increased and the solubility droped. To dissolve an increasingly formed precipitate, the mixture was diluted with MeOH (5 mL) and Pd black (10 mg) was added. The reaction mixture was placed under hydrogen atmosphere and stirred for 4 h. The mixture was diluted with DCM : EtOH = 1:1 (20 mL) and the solids were removed by filtration over regenerated cellulose filter (45 µm). The reaction

flask was filled with DCM - EtOH (3 mL, 1:1) and sonicated for 5 min, the solvent was filtered over regenerated cellulose filter (45 µm) and the procedure was repeated 3 times. The combined filtrates were concentrated to afford **5** (30 mg, 17.2 µmol, 98%) as white solid; R_f = 0.28 ($CHCl_3$ – MeOH – H_2O, 50:28:6).

For biological and functional studies compound **5** (10 mg) was purified by size exclusion chromatography on BioBeads SX1 (BioRad, column 800x1.5 mm, elution with toluene – DCM, 3:1). Appropriate fractions were collected and concentrated, the residue was re-dissolved in DMSO (4 mL), filtered through a regenerated cellulose filter (45 µm) and the filtrate was lyophilized to afford **5** (7.5 mg) as white fluffy solid.

^{1}H-NMR (600 MHz, $CDCl_3$ – MeOD, 3:1): δ 5.24-5.17 (m, 5H, H-1, H-1′, H-3, 2×β^{Myr}-C*H*), 5.13-5.09 (m, 1H, β^{Myr}-C*H*), 4.36-4.22 (m, 5H, H-2, H-4, H-4′, H-6a′, H-6b′), 4.13 (dd, 1H, ^3J = 3.2 Hz, ^3J = 9.2 Hz, H-3′), 3.90 (dd, 1H, ^3J = 3.9 Hz, ^3J = 13.2 Hz, H-6a), 3.81-3.74 (m, 2H, H-5′, H-6b), 3.72-3.69 (m, 1H, H-5), 3.52 (dd, 1H, ^3J = 1.6 Hz, ^3J = 3.3 Hz, H-2′), 3.49 (s, 3H, CH_3, Me), 2.68-2.59 (m, 4H, 2×α^{Myr}-CH_2), 2.46-2.38 (m, 2H, α^{Myr}-CH_2), 2.32-2.26 (m, 6H, α^{Myr}-CH_2, α^{Lau}-CH_2, α^{Cap}-CH_2), 1.64-1.53 (m, 12H, 3×γ^{Myr}-CH_2, β^{Myr}-CH_2, β^{Lau}-CH_2, β^{Cap}-CH_2), 1.35-1.22 (m, 102H, 51×CH_2), 0.88 (t, 18H, J = 7.0 Hz, 4×ω^{Myr}-CH_3, ω^{Lau}-CH_3, ω^{Cap}-CH_3).

^{31}P-NMR (243 MHz, $CDCl_3$ – MeOD, 3:1) δ 0.52, 0.41;

MS (-MALDI) m/z: calcd for $C_{91}H_{170}NO_{25}P_2$ $[M-H]^-$ 1739.154, found 1739.009.

Synthesis of 6

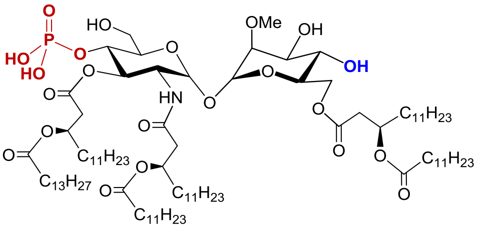

6-*O*-[(*R*)-3-(Dodecanoyloxy)tetradecanoyl]-2-*O*-methyl-α-D-mannopyranosyl-(1↔1)-2-deoxy-2-[(*R*)-3-(dodecanoyloxy)tetradecanoylamino]-4-*O*-phosphoryl-3-*O*-[(*R*)-3-(tetradecanoyloxy)tetradecanoyl]-α-D-glucopyranoside (6).

Palladium black (5 mg, 47 µmol) was added to a solution of **25** (10 mg, 4.9 µmol) in toluene – MeOH (3 mL, 1:1), the mixture was placed under hydrogen atmosphere and stirred for 20 h at r.t. The mixture was diluted with DCM – EtOH, 1:1 (5 mL) and the solids were removed by filtration over regenerated cellulose filter (45 µm) and the filtrate was concentrated to afford to afford **6** (8 mg, 4.7 µmol, 97%) as white solid; R_f = 0.59 ($CHCl_3$ – MeOH – H_2O, 50:28:6).

For biological studies, compound **6** was purified by size exclusion chromatography on BioBeads SX1 (BioRad, column 800 x 1.5 mm, elution with toluene – DCM, 3:1). Appropriate fractions were collected and concentrated, the residue was dissolved in DMSO (2 mL) filtered through a regenerated cellulose filter (45 µm) and freeze dried to afford **6** (5.8 mg, 3.4 µmol, 77%) as white solid.

^{1}H-NMR (600 MHz, $CDCl_3$ – MeOD, 3 : 1): δ 7.12 (d, 1H, $^3J_{NH,2}$ = 8.5 Hz, N*H*), 5.25-5.16 (m, 5H, H-1, H-1′, H-3, 2×β^{Myr}-C*H*), 5.14-5.09 (m, 1H, β^{Myr}-C*H*), 4.34-4.29 (m, 2H, H-4, H-6a′), 4.28-4.22 (m, 2H, H-2, H-6b′), 3.92-3.89 (m, 2H, H-3′, H-6a), 3.79-3.74 (m, 1H, H-6b), 3.73-3.64 (m, 3H, H-4′, H-5′, H-5), 3.49-3.47 (m, 1H, H-2′), 3.48 (s, 3H, CH_3, Me), 2.69-2.58 (m, 4H, 2×α^{Myr}-CH_2), 2.47-2.40 (m, 2H, α^{Myr}-CH_2), 2.32-2.27 (m, 6H, α^{Myr}-CH_2, 2×α^{Lau}-C*H*), 1.65-1.54 (m, 12H, β^{Myr}-CH_2, 2×β^{Lau}-CH_2, 3×γ^{Myr}-CH_2), 1.37-1.22 (m, 106H, 53×CH_2), 0.89 (t, 18H, J = 7.0 Hz, 4×ω^{Myr}-CH_3, 2×ω^{Lau}-CH_3);

^{31}P-NMR (243 MHz, $CDCl_3$ – MeOD, 3:1): δ 0.35;

MS (-MALDI): m/z calcd for $C_{93}H_{173}NO_{22}P$ $[M-H]^-$ 1687.219; found 1687.250.

Synthesis of 7

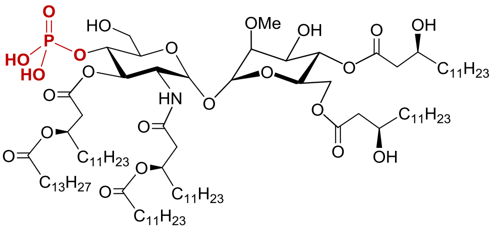

4,6-di-*O*-[(*R*)-3-hydroxytetradecanoyl]-2-*O*-methyl-α-D-mannopyranosyl-(1↔1)-2-deoxy-2-[(*R*)-3-(dodecanoyloxy)tetradecanoylamino]-4-*O*-phosphoryl-3-*O*-[(*R*)-3-(tetradecanoyloxy)tetradecanoyl]-α-D-glucopyranoside (7).

Palladium black (6 mg, 56 µmol) was added to a stirred solution of **30** (7.7 mg, 3.4 µmol) in toluene – MeOH (2 mL, 2:1), the mixture was stirred under hydrogen atmosphere for 4 h at r.t. The mixture was diluted with MeOH (1 mL) and Pd black was added stepwise in four portions (5 mg, 47 µmol each, every 5h). The stirring was continued for 24 h (4 h after the last addition of Pd black), the mixture was diluted with toluene – MeOH, 1:1 (5 mL) and the solids were removed by filtration over regenerated cellulose filter (45 µm). The filtrate was concentrated to afford **7** (5.5 mg, 3.2 µmol, 93%). R_f = 0.53 ($CHCl_3$ – MeOH – H_2O, 50:28:6).

For biological studies, the residue was purified by size exclusion chromatography on Sephadex LH-20 (80 cm × 1 cm, toluene – MeOH, 1:1). Appropriate fractions were concentrated, the residue was dissolved in DMSO (1 mL), filtered through a regenerated cellulose filter (45 µm) and freeze dried to afford **7** (4.2 mg, 2.4 µmol, 68%).

^{1}H-NMR (600 MHz, $CDCl_3$ – MeOD, 3 : 1): δ 7.22 (d, 1H, $3J_{NH,2}$ = 8.5 Hz, N*H*), 5.24-5.18 (m, 4H, H-1, H-1′, H-3, β^{Myr}-C*H*), 5.12-5.08 (m, 1H, β^{Myr}-C*H*), 5.10 (t, 1H, $3J_{4',5'}$ = $3J_{4',3'}$ = 9.9 Hz, H-4′), 4.35-4.27 (m, 1H, H-4), 4.23-4.12 (m, 3H, H-2, H-6a′, H-6b′), 4.11-4.09 (m, 1H, H-3), 4.04-3.99 (m, 2H, 2×β^{Myr}-C*H*), 3.95 (br. d, 1H, *J* = 12.0 Hz, H-6a), 3.84-3.81 (m, 1H, H-5′), 3.72 (d, 1H, *J* = 12.7 Hz, H-6b), 3.69-3.64 (m, 1H, H-5), 3.53 (dd, 1H, *J* = 1.4 Hz, *J* = 3.3 Hz, H-2′), 3.50 (s, 3H, CH_3, Me), 2.70-2.62 (m, 2H, α-CH_2), 2.55-2.47 (m, 3H, α-CH_2), 2.44-2.39 (m, 3H, α-CH_2), 2.33-2.28 (m, 4H, α-CH_2), 1.66-1.41 (m, 12H, 4×γ^{Myr}-CH_2, β^{Lau}-CH_2, β^{Lau}-CH_2), 1.39-1.21 (m, 108H, 54×CH_2), 0.89 (t, 18H, *J* = 7.0 Hz, 5×ω^{Myr}-CH_3, ω^{Lau}-CH_3).

^{31}P-NMR (243 MHz, $CDCl_3$ – MeOD, 3 : 1) δ 0.73;

HRMS (-MALDI) m/z: calcd for $C_{95}H_{177}NO_{23}P$ $[M-H]^-$ 1731.246, found 1731.193.

References

[1] M. Akamatsu, Y. Fujimoto, M. Kataoka, Y. Suda, S. Kusumoto, K. Fukase, *Bioorg.Med.Chem.* **2006,** *14*, 6759-6777.
[2] H. G. Bazin, L. S. Bess, M. T. Livesay, K. T. Ryter, C. L. Johnson, J. S. Arnold, D. A. Johnson, *Tetrahedron Lett.* **2006,** *47*, 2087-2092.
[3] S. T. Ishizaka, L. D. Hawkins, *Expert Rev.Vaccines* **2007,** *6*, 773-784.
[4] D. A. Johnson, *Curr.Top.Med.Chem.* **2008,** *8*, 64-79.
[5] T. Shibata, Y. Motoi, N. Tanimura, N. Yamakawa, S. Akashi-Takamura, K. Miyake, *Int.Immunol.* **2011,** *23*, 503-510.
[6] H. H. Jensen, L. U. Nordstrom, M. Bols, *J.Am.Chem.Soc.* **2004,** *126*, 9205-9213.
[7] F. Adanitsch, S. Ittig, J. Stöckl, A. Oblak, M. Haegman, R. Jerala, R. Beyaert, P. Kosma, A. Zamyatina, *J.Med.Chem.* **2014,** *57*, 8056-8071.
[8] D. Artner, A. Oblak, S. Ittig, J. A. Garate, S. Horvat, C. Arrieumerlou, A. Hofinger, C. Oostenbrink, R. Jerala, P. Kosma, A. Zamyatina, *ACS Chem.Biol.* **2013,** *8*, 2423-2432.
[9] M. A. Chaube, S. S. Kulkarni, *Trends Carbohydr.Res.* **2012,** *4*, 1-19.
[10] Y. Ito, H. Ando, M. Wada, T. Kawai, Y. Ohnish, Y. Nakahara, *Tetrahedron* **2001,** *57*, 4123-4132.
[11] C. D. Leigh, C. R. Bertozzi, *J.Org.Chem.* **2008,** *73*, 1008-1017.
[12] Y. C. L. Lu, B. Ghosh, K. K. T. Mong, *Chem.Eur.J.* **2017,** *23*, 6905-6918.

[13] H. I. Duynstee, M. J. van Vliet, G. A. van der Marel, J. H. van Boom, *Eur.J.Org.Chem.* **1998,** *1998*, 303-307.
[14] M. Färnbäck, L. Eriksson, G. Widmalm, *Acta Crystallogr.Sect.E* **2004,** *60*, 1483-1485.
[15] T. C. Baddaley, C. M. Clow, P. J. Cox, A. M. McLaughlin, J. L. Wardell, *Acta Cryst.* **2001,** *E57*, 456-457.
[16] T. C. Baddaley, C. M. Clow, P. J. Cox, I. G. Davidson, R. A. Howie, J. L. Wardell, *Acta Crystallogr., Sect.E* **2002,** *E58*, 476-477.
[17] K. Bock, J. Defaye, H. Driguez, E. Bar-Guilloux, *Eur.J.Biochem.* **1983,** *132*, 595-600.
[18] G. M. Brown, D. C. Rohrer, B. Berking, C. A. Beevers, R. O. Gould, R. Simpson, *Acta Cryst.Sect.B* **1972,** *28*, 3145-3158.
[19] M. K. Dowd, P. J. Reilly, A. D. French, *J.Comput.Chem.* **1992,** *13*, 102-114.
[20] E. D. Stevens, M. K. Dowd, G. P. Johnson, A. D. French, *Carbohydr.Res.* **2010,** *345*, 1469-1481.
[21] S. M. Clow, P. J. Cox, G. I. Gilmore, J. L. Wardell, *Acta Cryst.* **2001,** *E57*, 77-78.
[22] S. Tsuchiya, Y. Kobayashi, Y. Goto, H. Okumura, S. Nakae, T. Konno, K. Tada, *Cancer Res.* **1982,** *42*, 1530-1536.
[23] C. Gallego, D. Golenbock, M. A. Gomez, N. G. Saravia, *Infect.Immun.* **2011,** *79*, 2871-2879.
[24] J. Shi, Y. Zhao, Y. Wang, W. Gao, J. Ding, P. Li, L. Hu, F. Shao, *Nature* **2014,** *514*, 187-192.
[25] D. S. Keegan, S. R. Hagen, D. A. Johnson, *Tetrahedron: Asymmetry* **1996,** *7*, 3559-3564.

NMR and Mass- spectra

^{1}H and ^{13}C NMR spectra of target compounds and synthetic intermediates

HSQC-NMR and MALDI-TOF spectra of target compounds

(10): ^{1}H-NMR, 300 MHz, $CDCl_3$

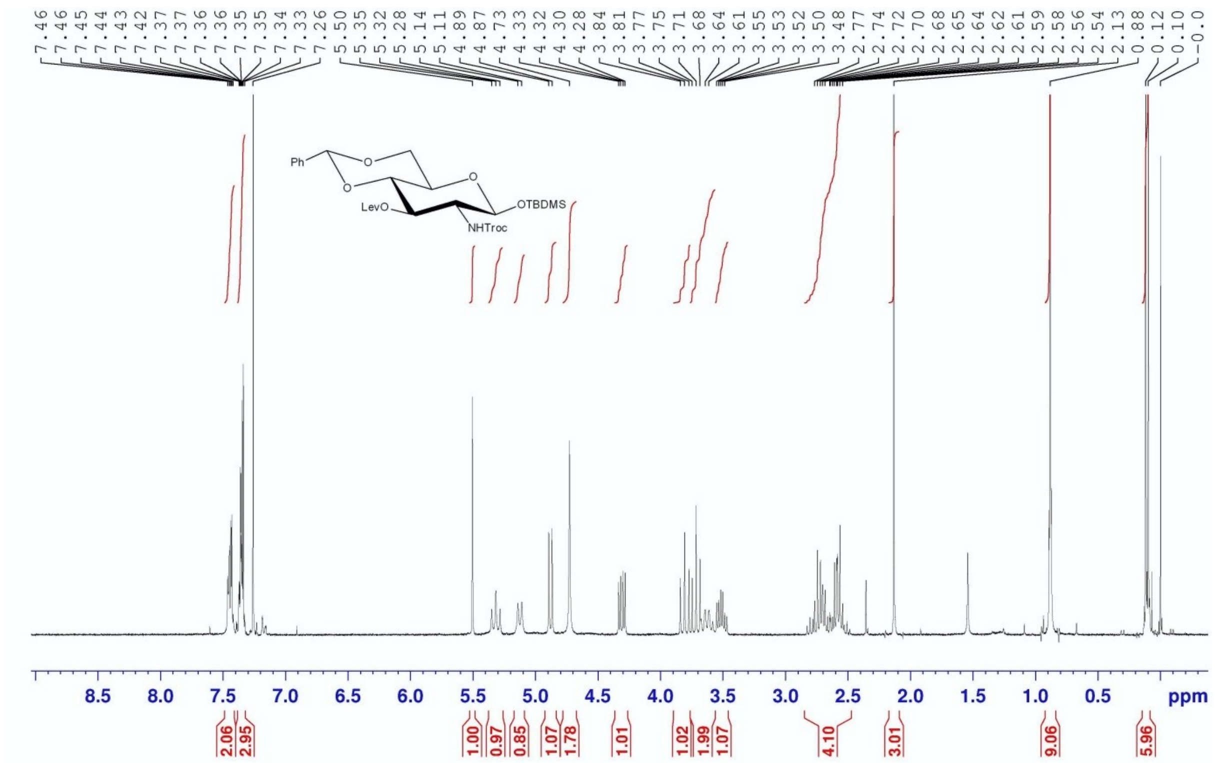

(10): ^{13}C-NMR, APT, 75 MHz, $CDCl_3$

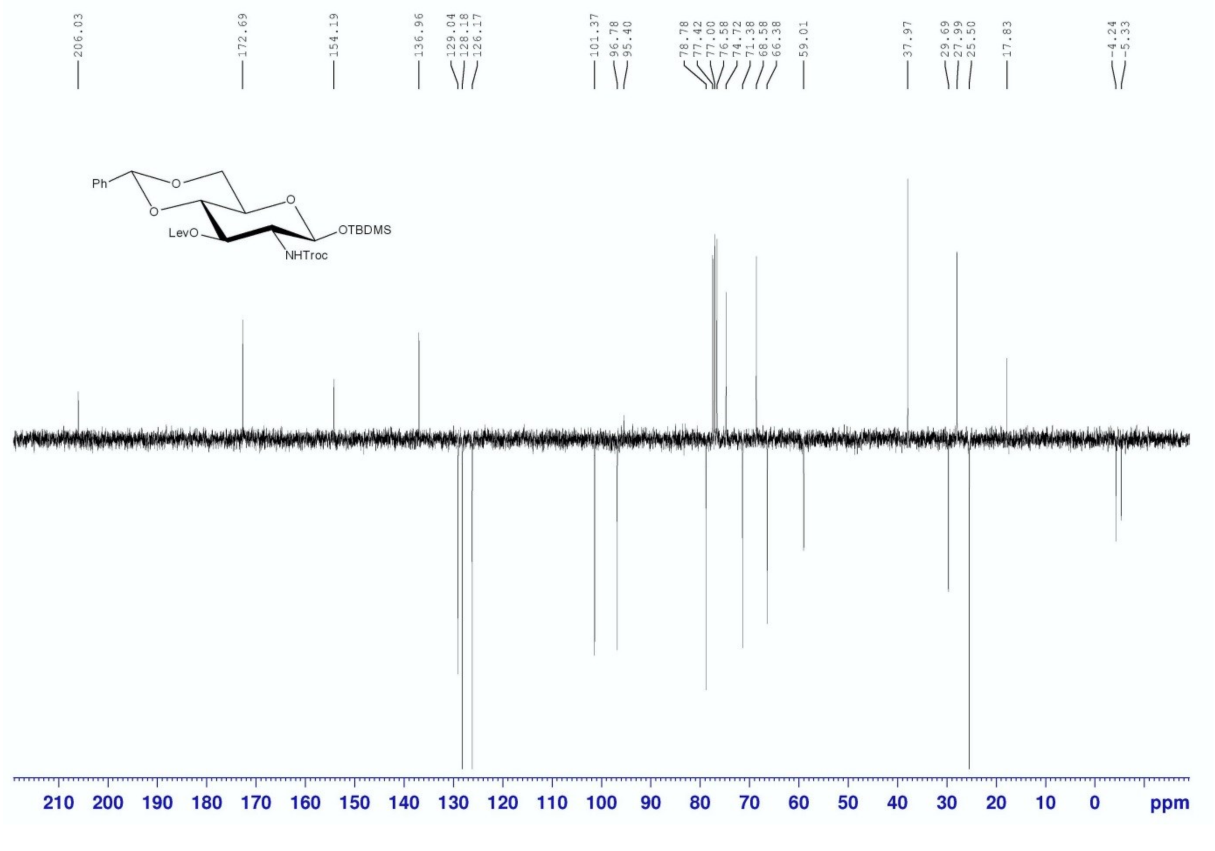

(11): ^{1}H-NMR, 600 MHz, $CDCl_3$

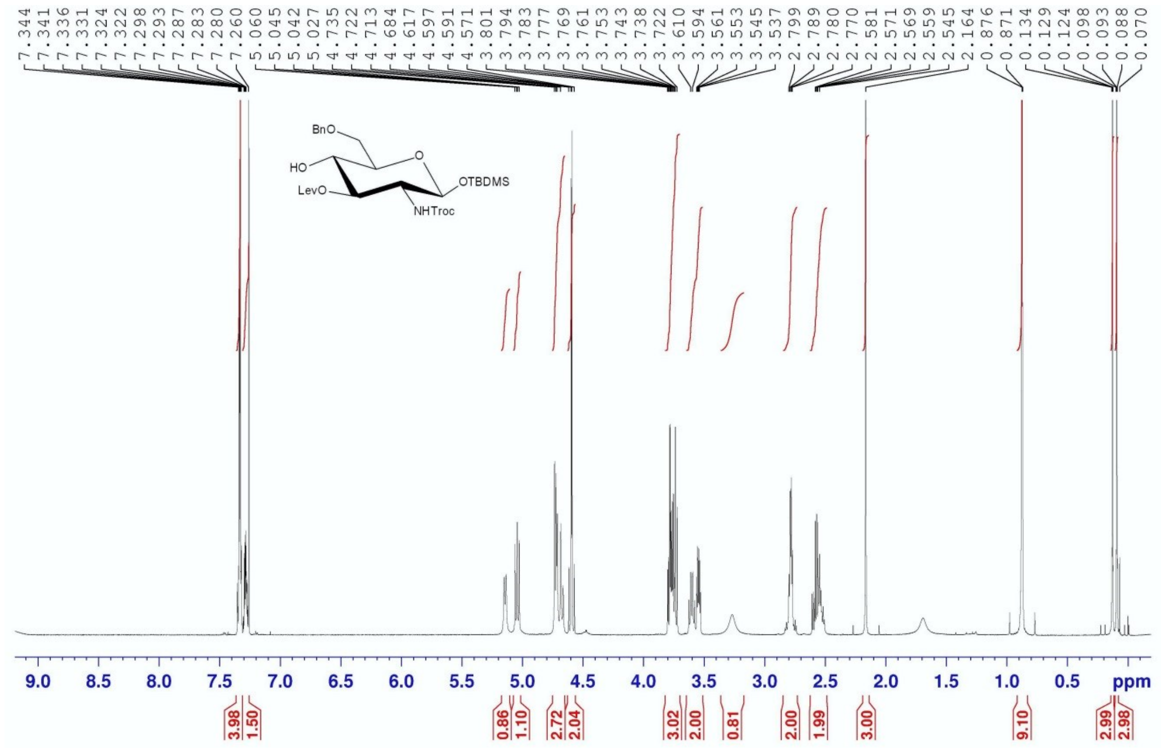

(11): ^{13}C-NMR, 101 MHz, $CDCl_3$

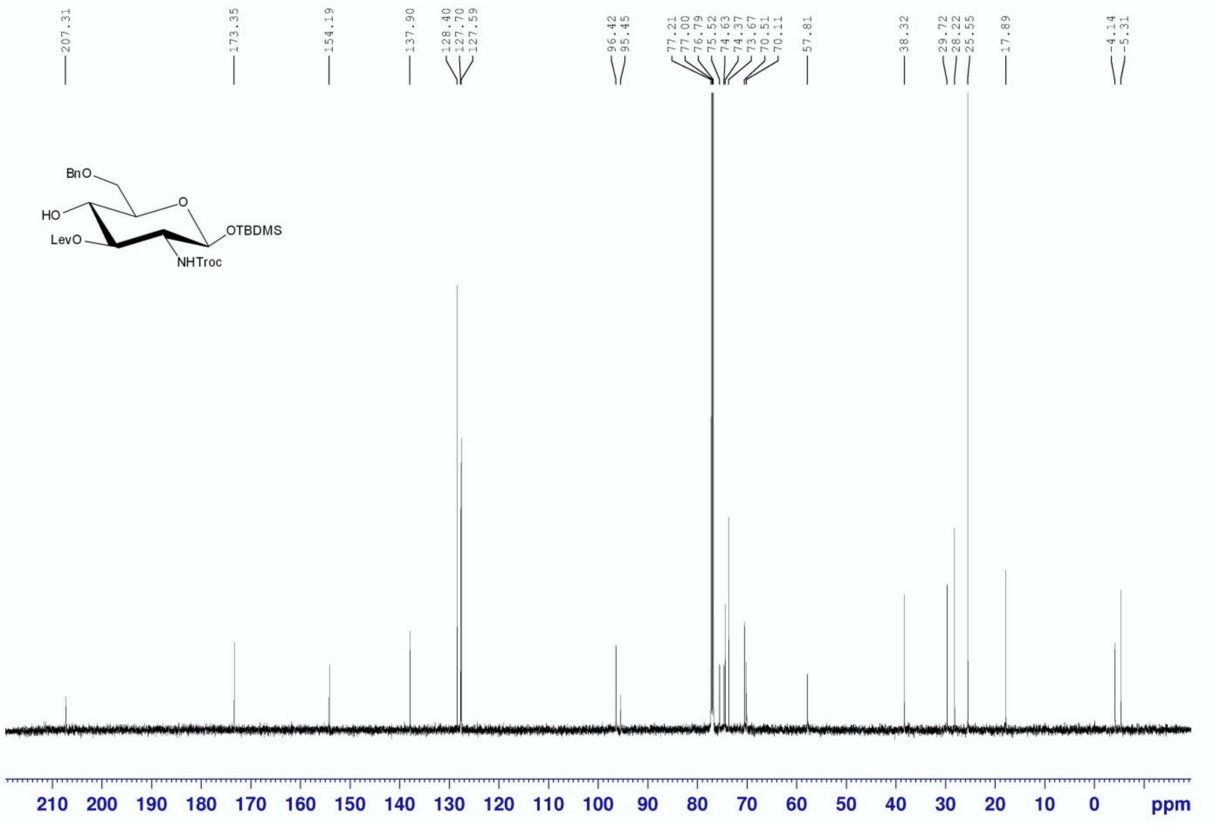

(12): ^{1}H-NMR, 600 MHz, $CDCl_3$

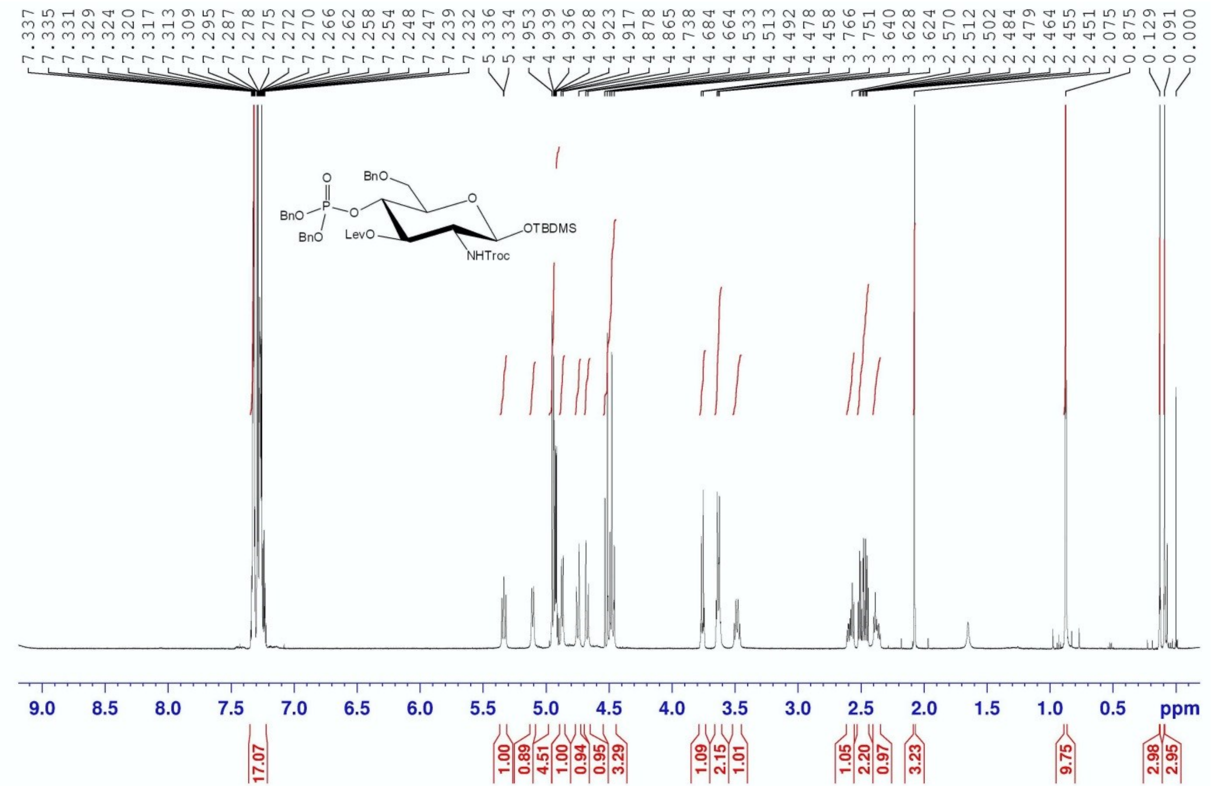

(12): ^{13}C-NMR, APT, 151 MHz, $CDCl_3$

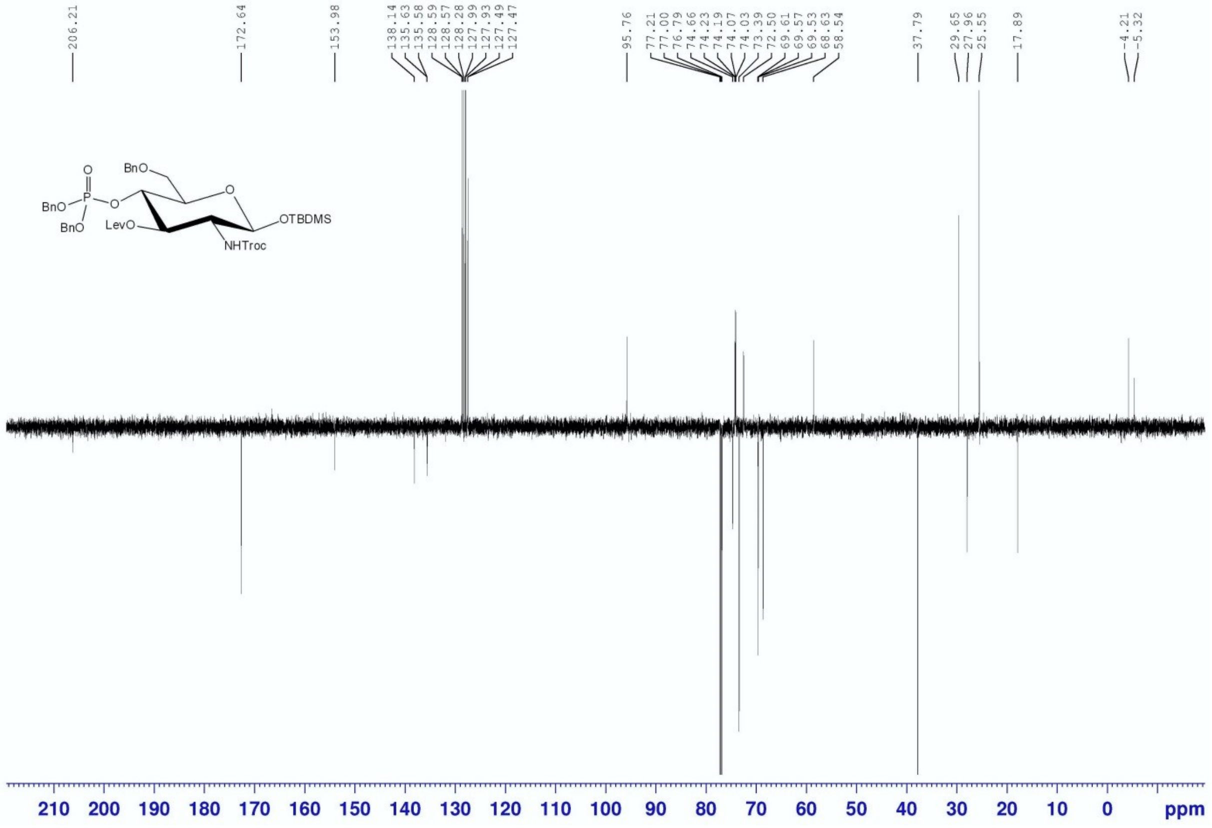

(13): ^{1}H-NMR, 300 MHz, $CDCl_3$; α-anomer

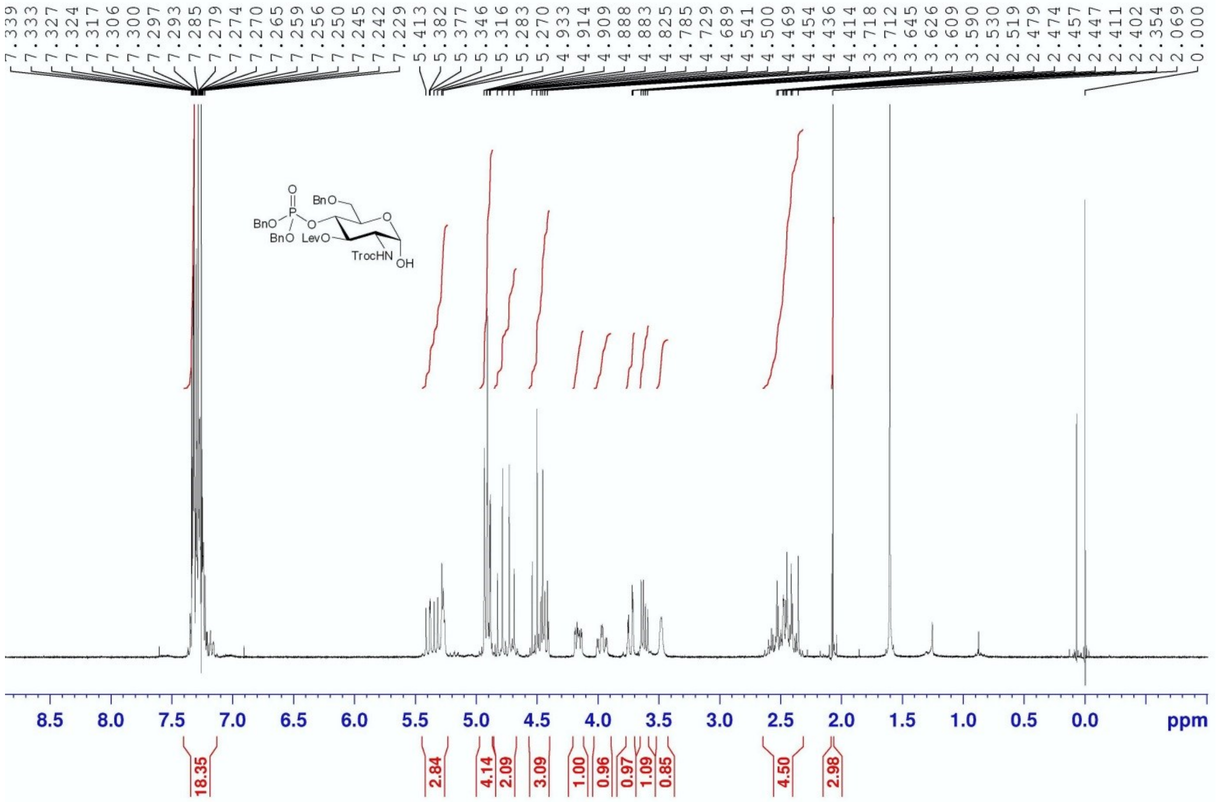

(13): ^{13}C-NMR, 75 MHz, $CDCl_3$; α-anomer

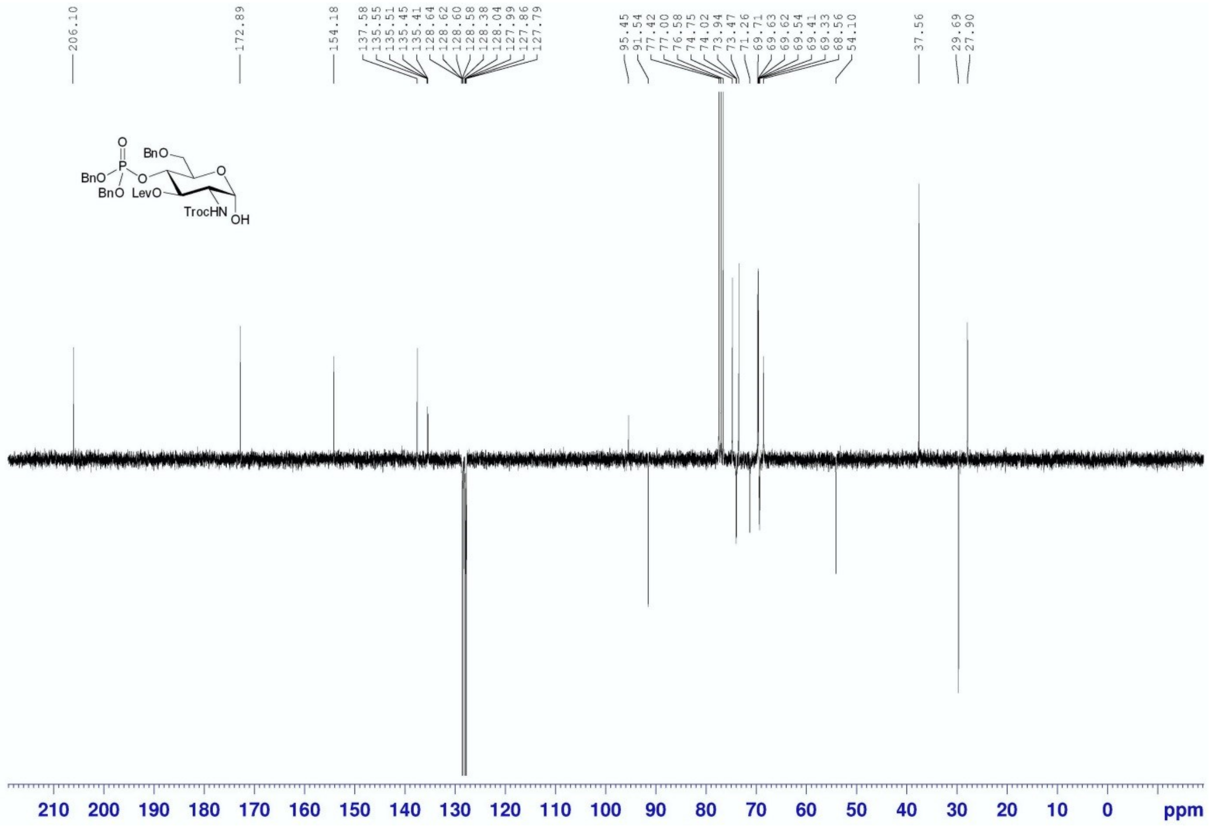

(18): ^{1}H-NMR, 600 MHz, $CDCl_3$

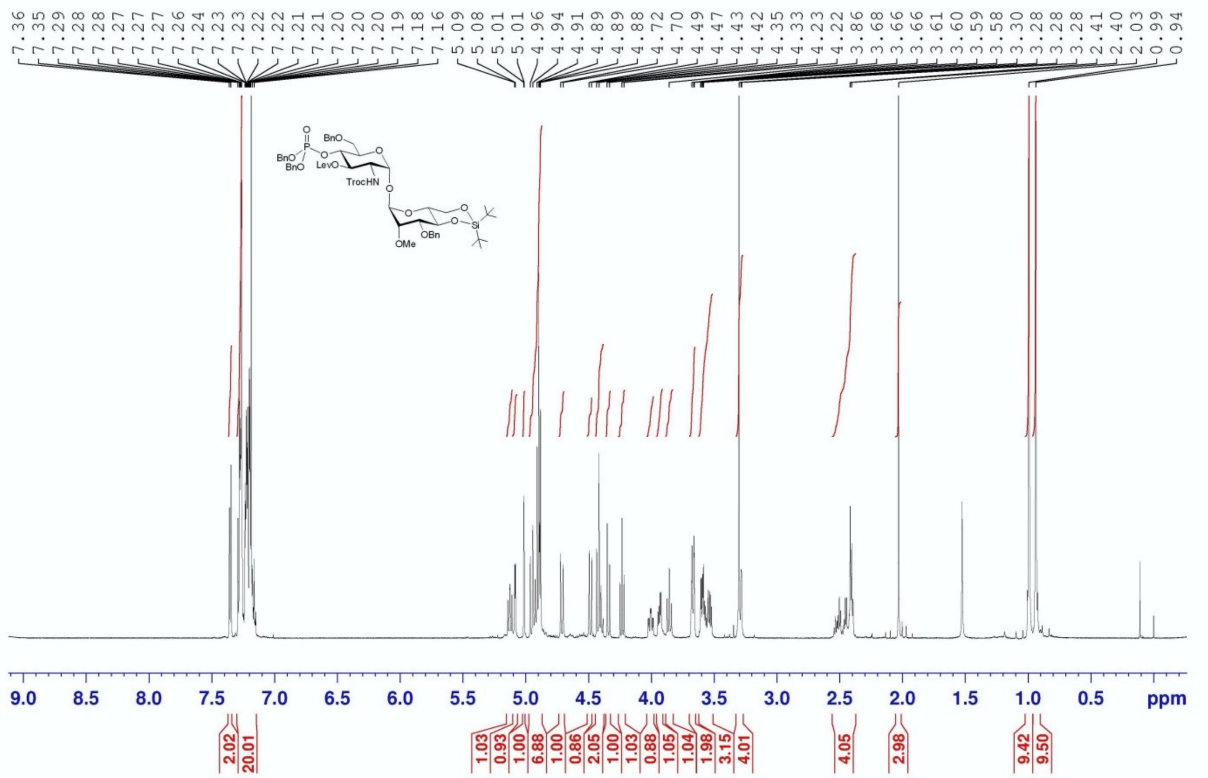

(18): ^{13}C-NMR, APT, 151 MHz, $CDCl_3$

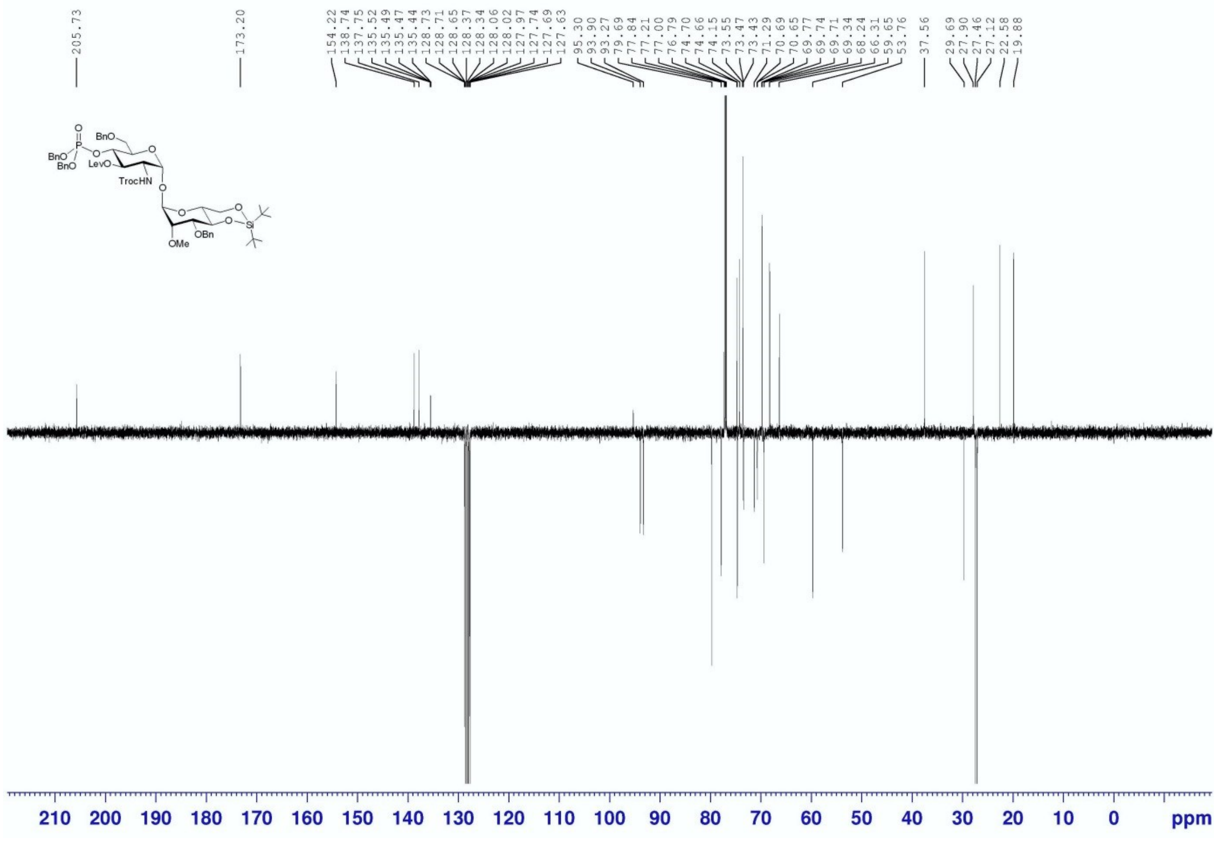

(19): ^{1}H-NMR, 600 MHz, $CDCl_3$

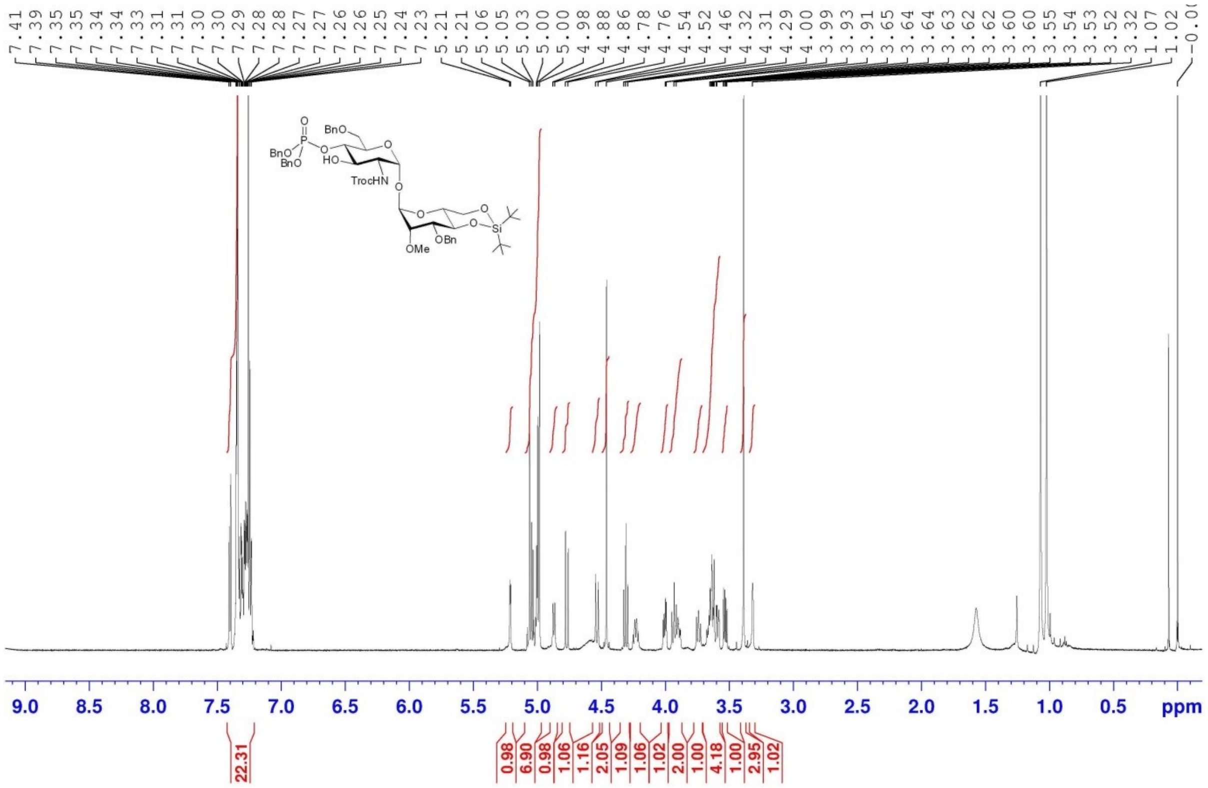

(19): ^{13}C-NMR, APT, 151 MHz, $CDCl_3$

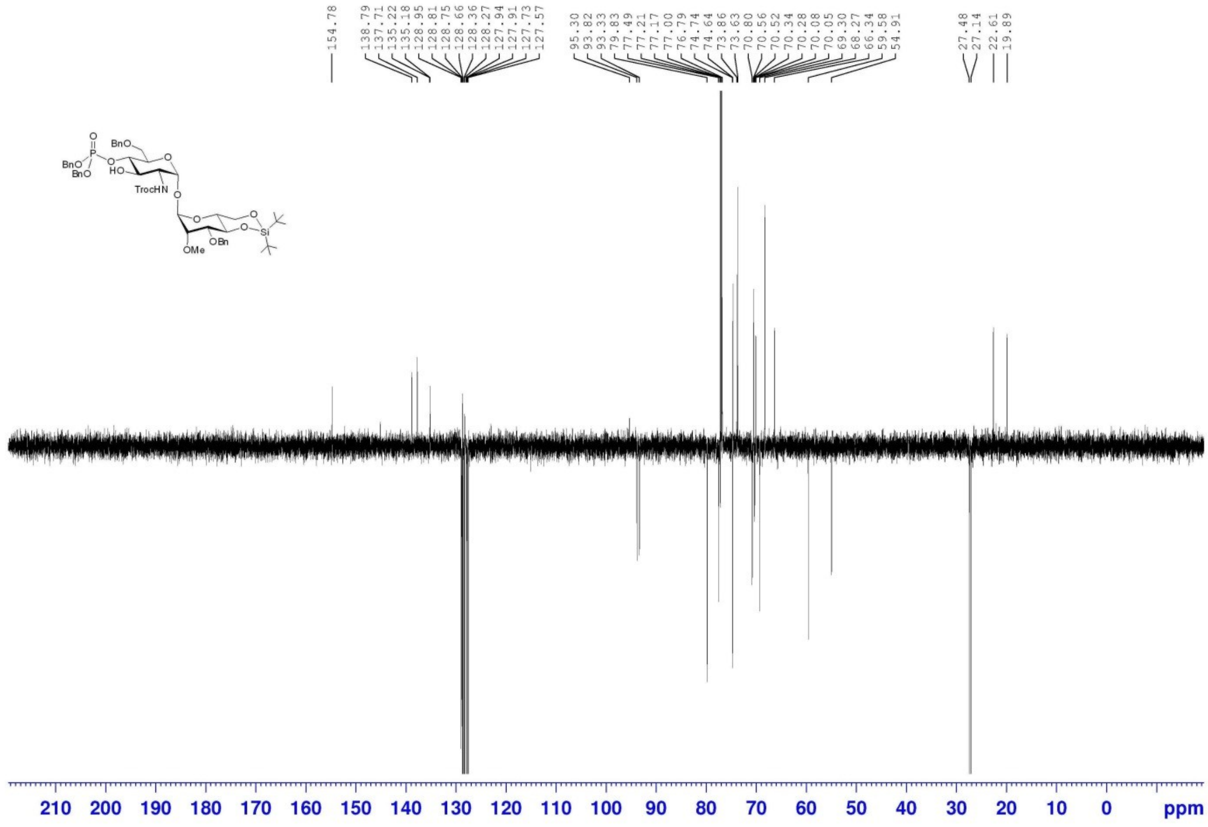

(20): ^{1}H-NMR, 600 MHz, $CDCl_3$

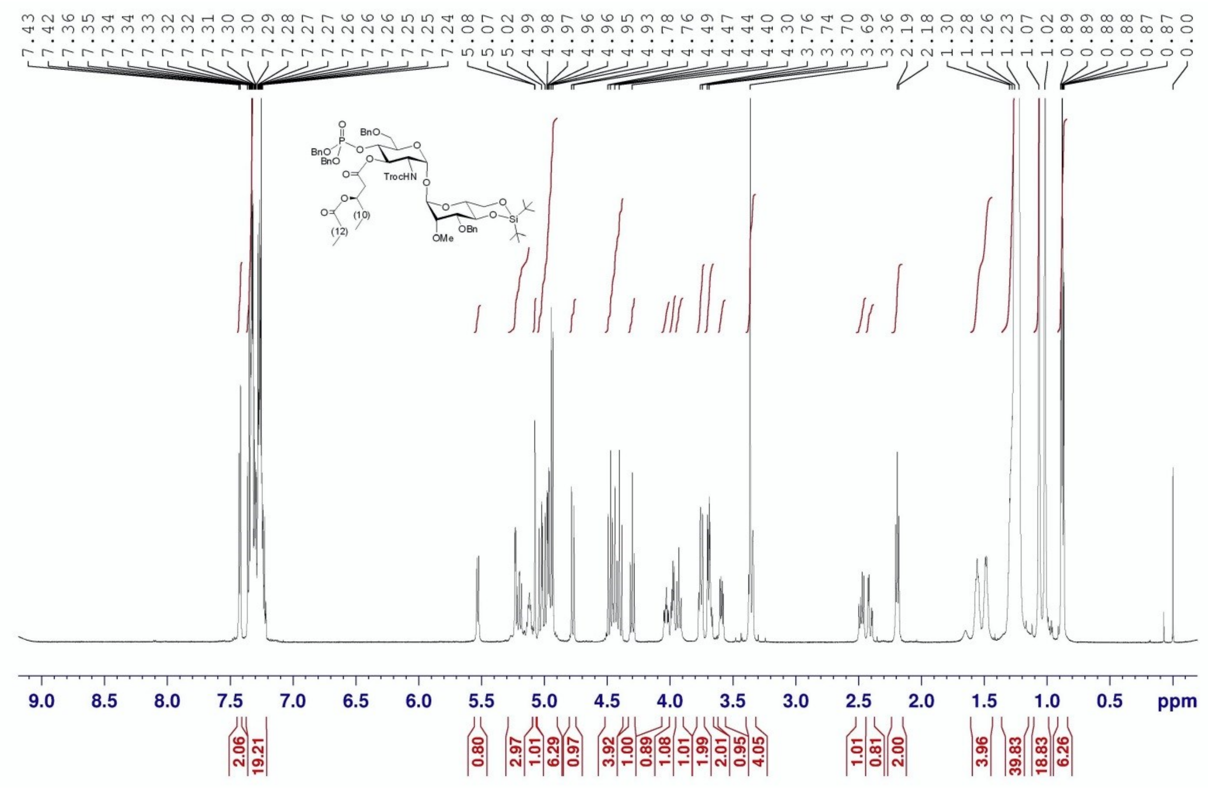

(20): ^{13}C-NMR, APT, 151 MHz, $CDCl_3$

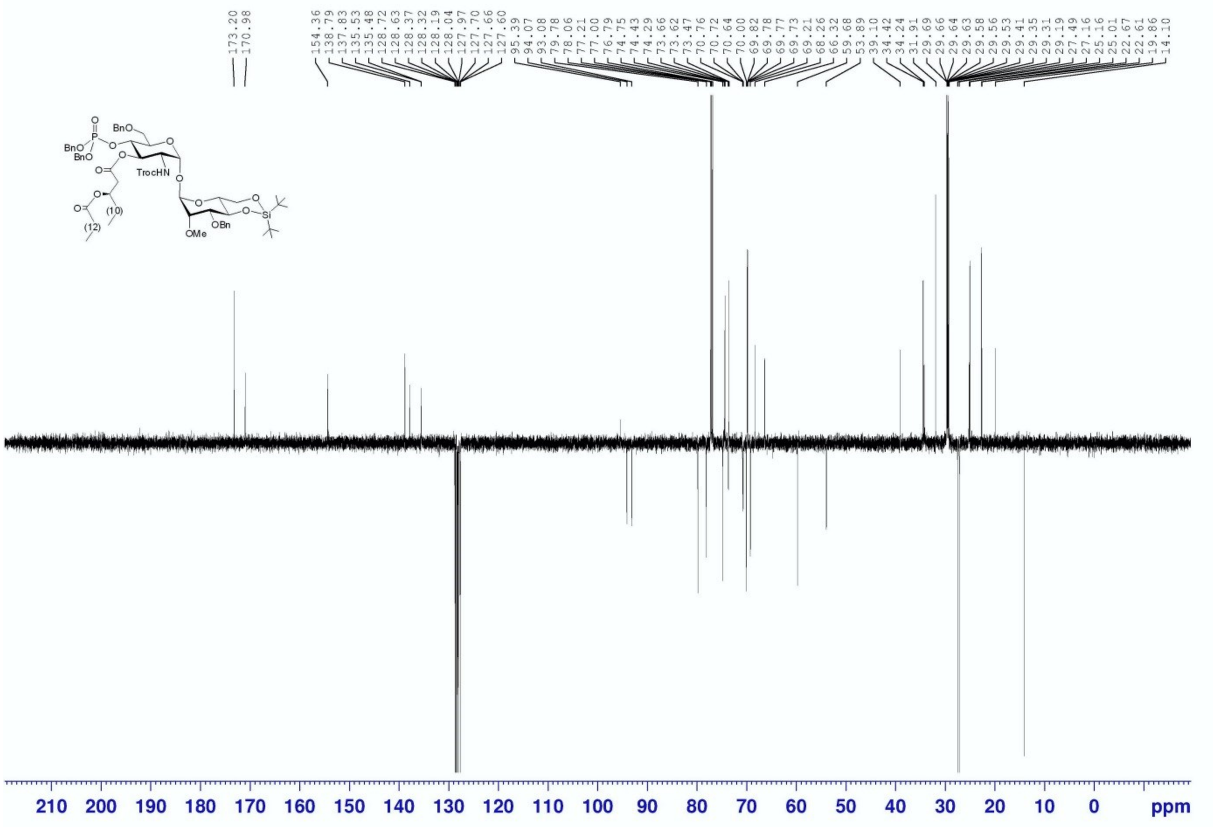

(21): ^{1}H-NMR, 600 MHz, CDCl$_3$

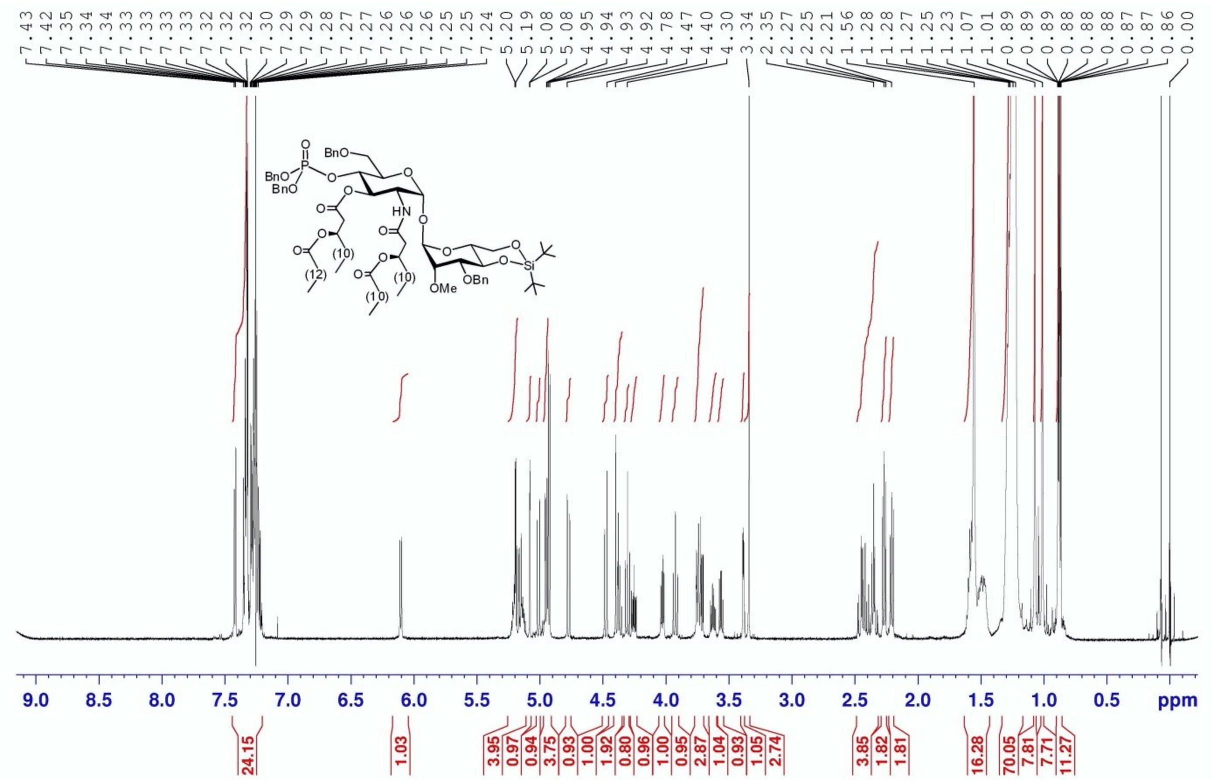

(21): ^{13}C-NMR, APT, 151 MHz, CDCl$_3$

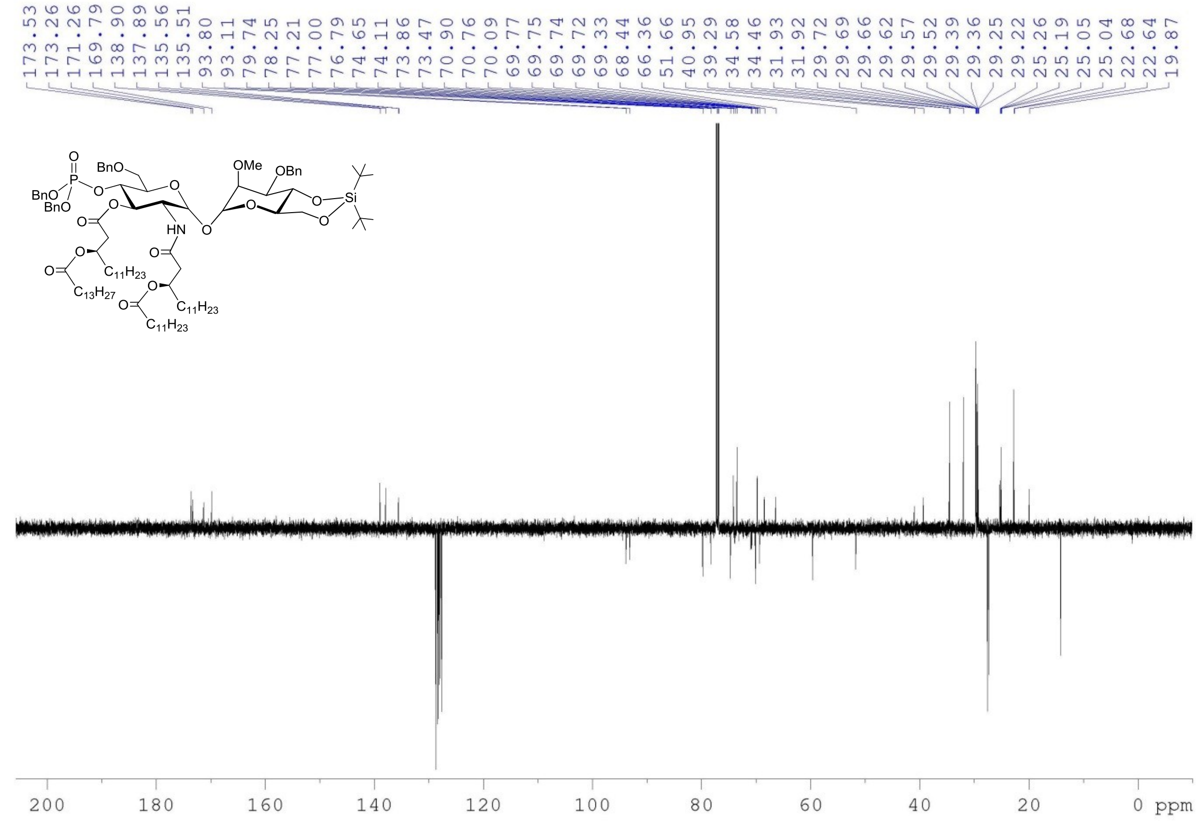

(22): ^{1}H-NMR, 600 MHz, $CDCl_3$

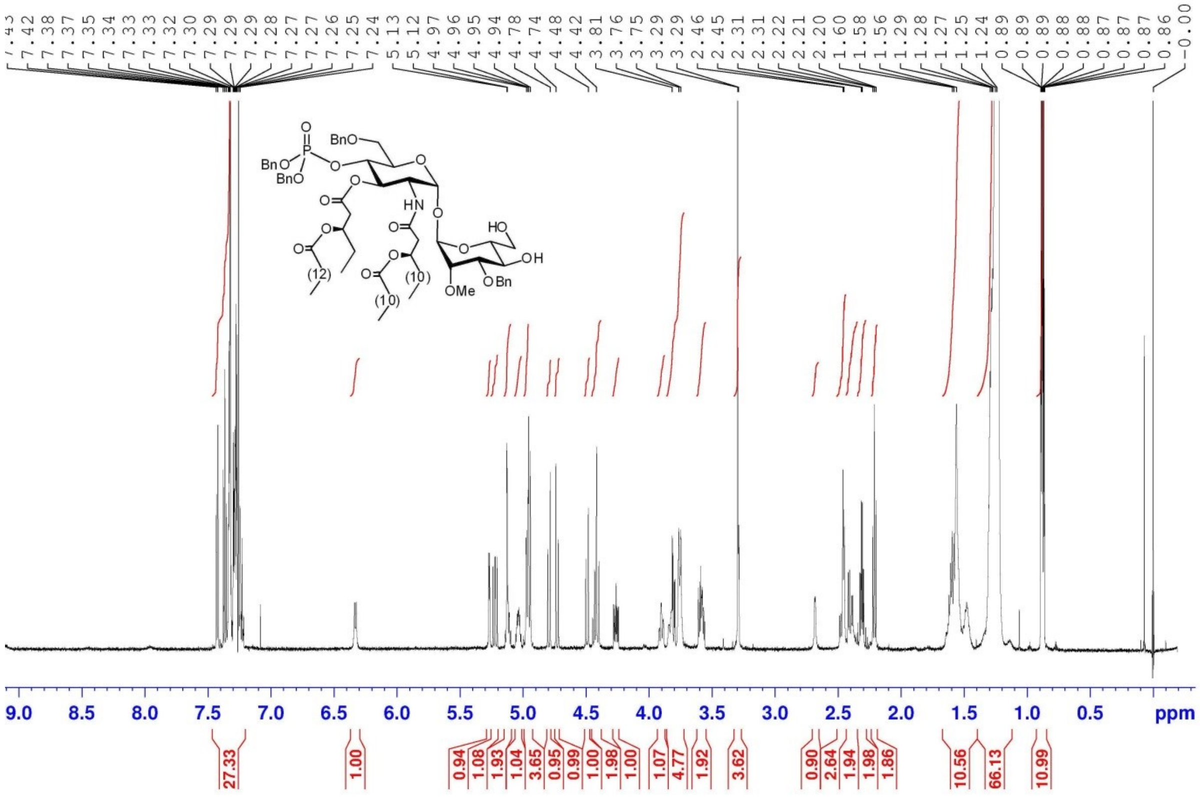

(22): ^{13}C-NMR, APT, 151 MHz, $CDCl_3$

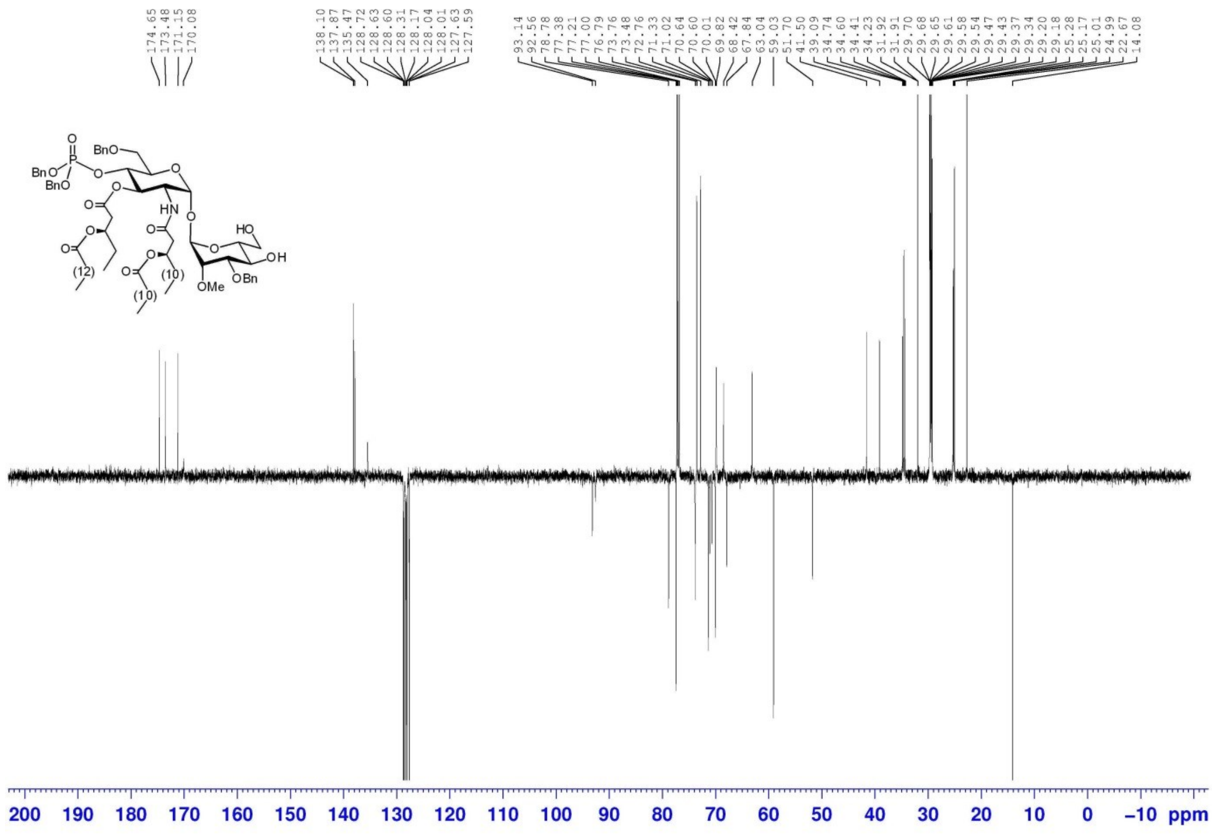

(23): ^{1}H-NMR, 600 MHz, $CDCl_3$

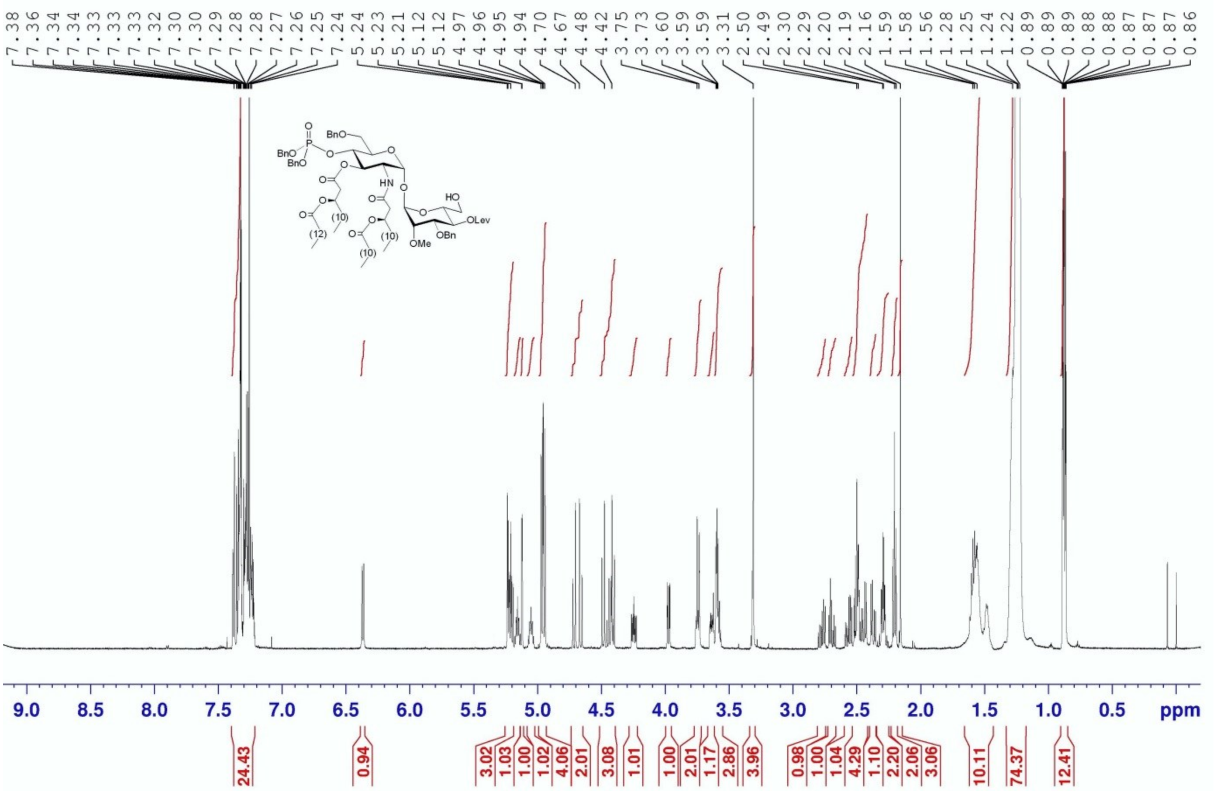

(23): ^{13}C-NMR, APT, 151 MHz, $CDCl_3$

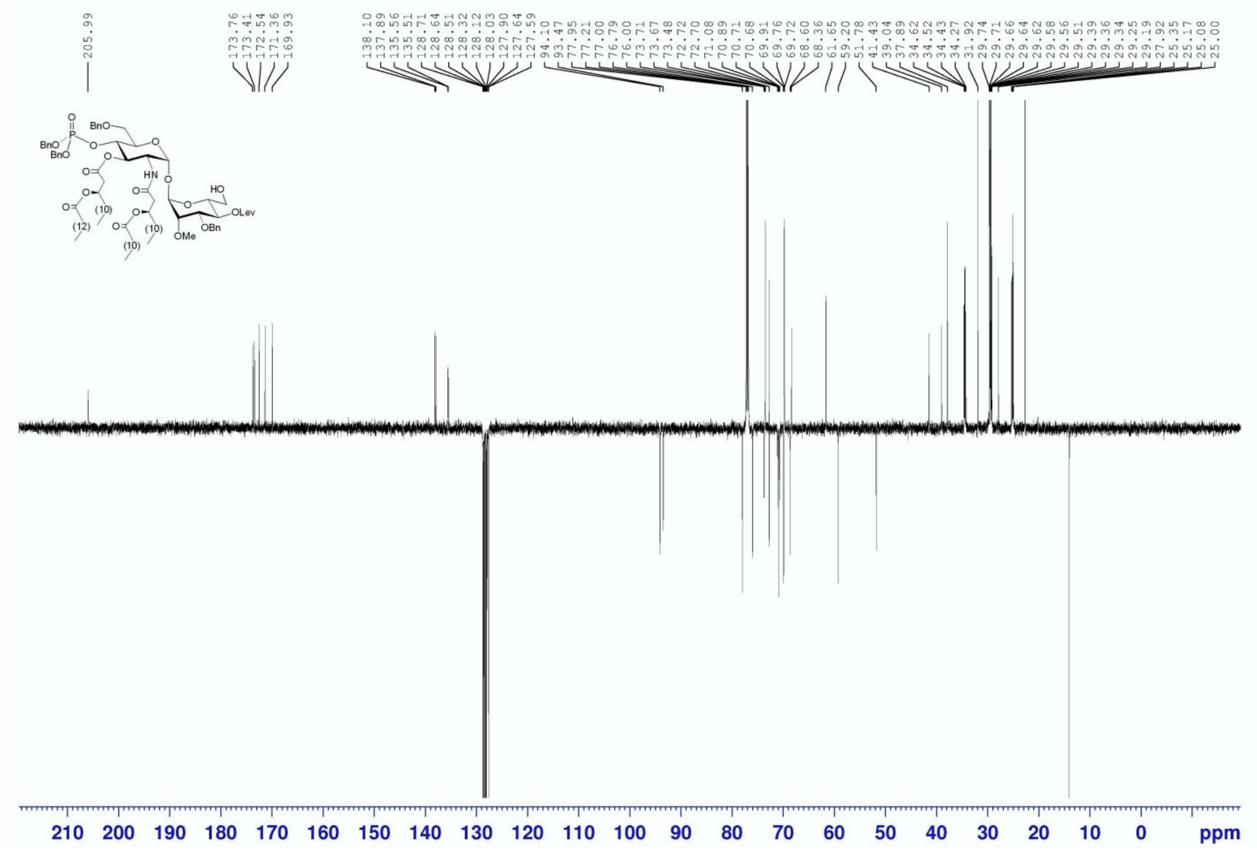

(24): ^{1}H-NMR, 600 MHz, $CDCl_3$

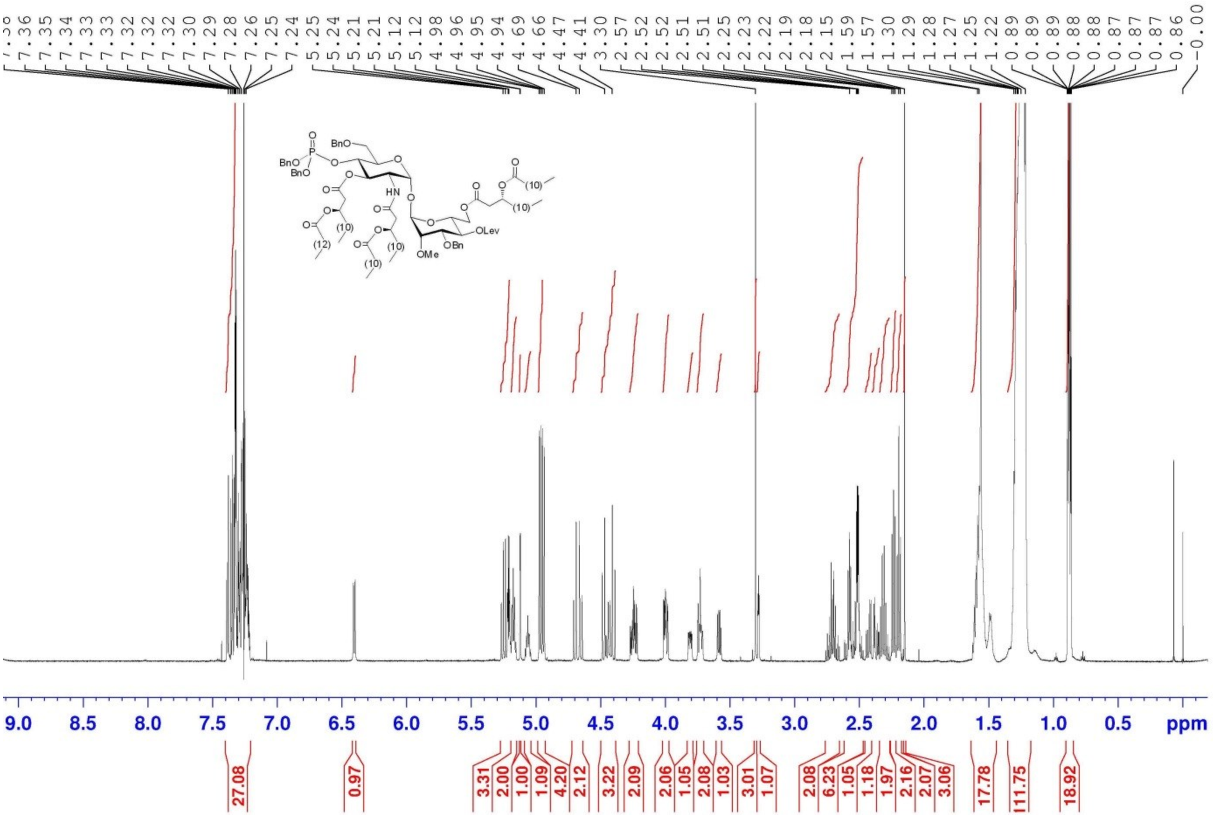

(24): ^{13}C-NMR, APT, 151 MHz, $CDCl_3$

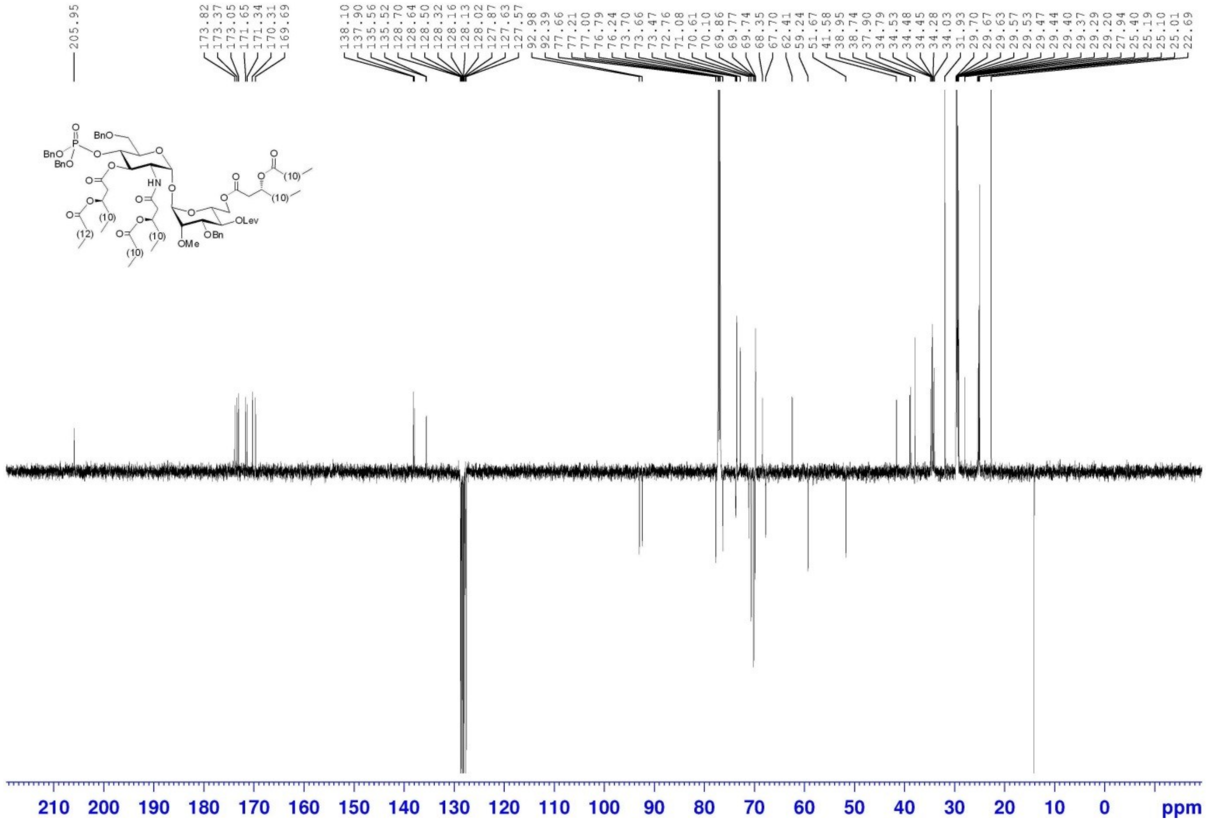

(25): ^{1}H-NMR, 600 MHz, $CDCl_3$

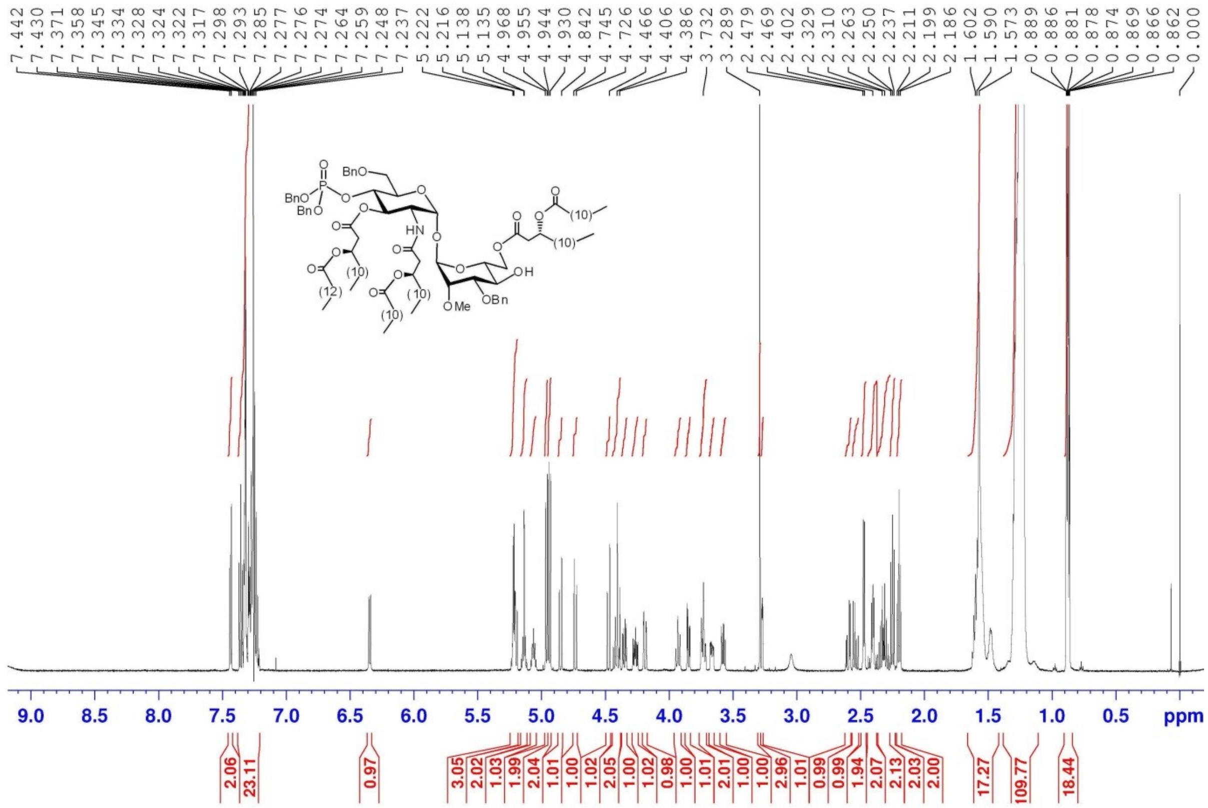

(25): ^{13}C-NMR, APT, 151 MHz, $CDCl_3$

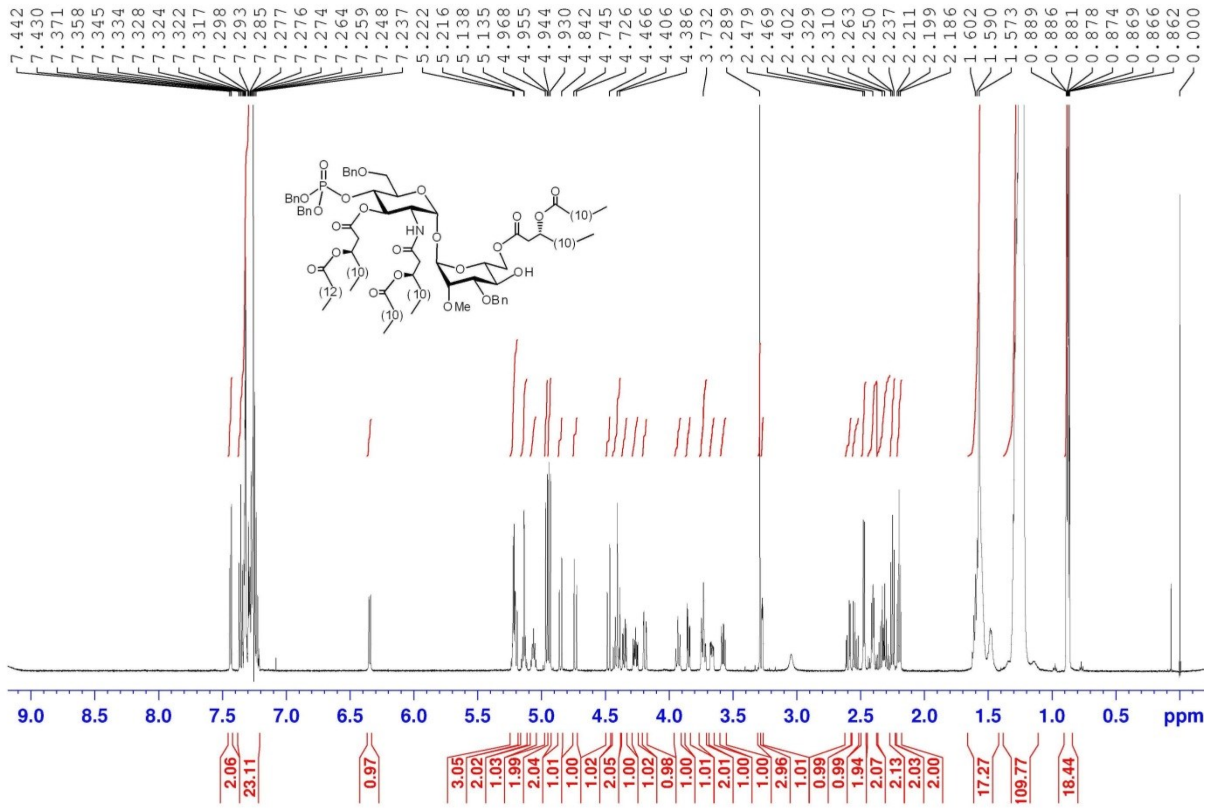

(26): ^{1}H-NMR, 600 MHz, CDCl$_3$

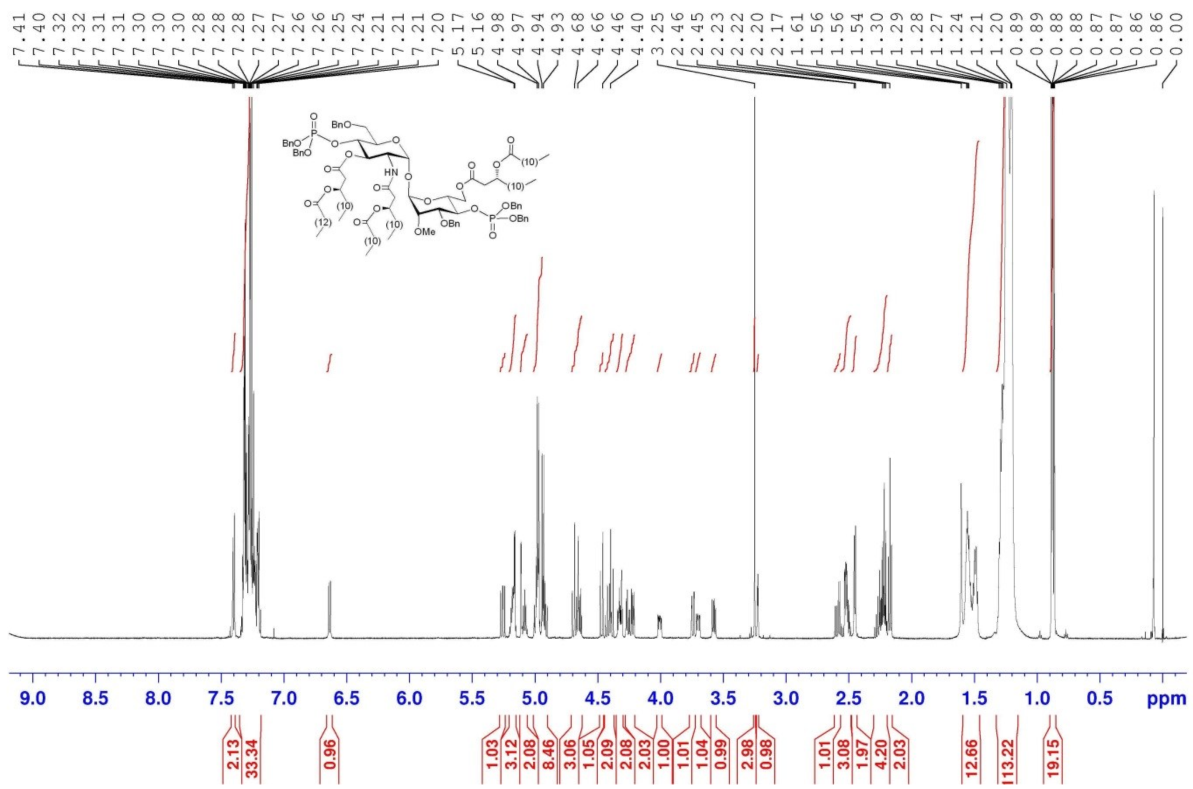

(26): ^{13}C-NMR, APT, 151 MHz, CDCl$_3$

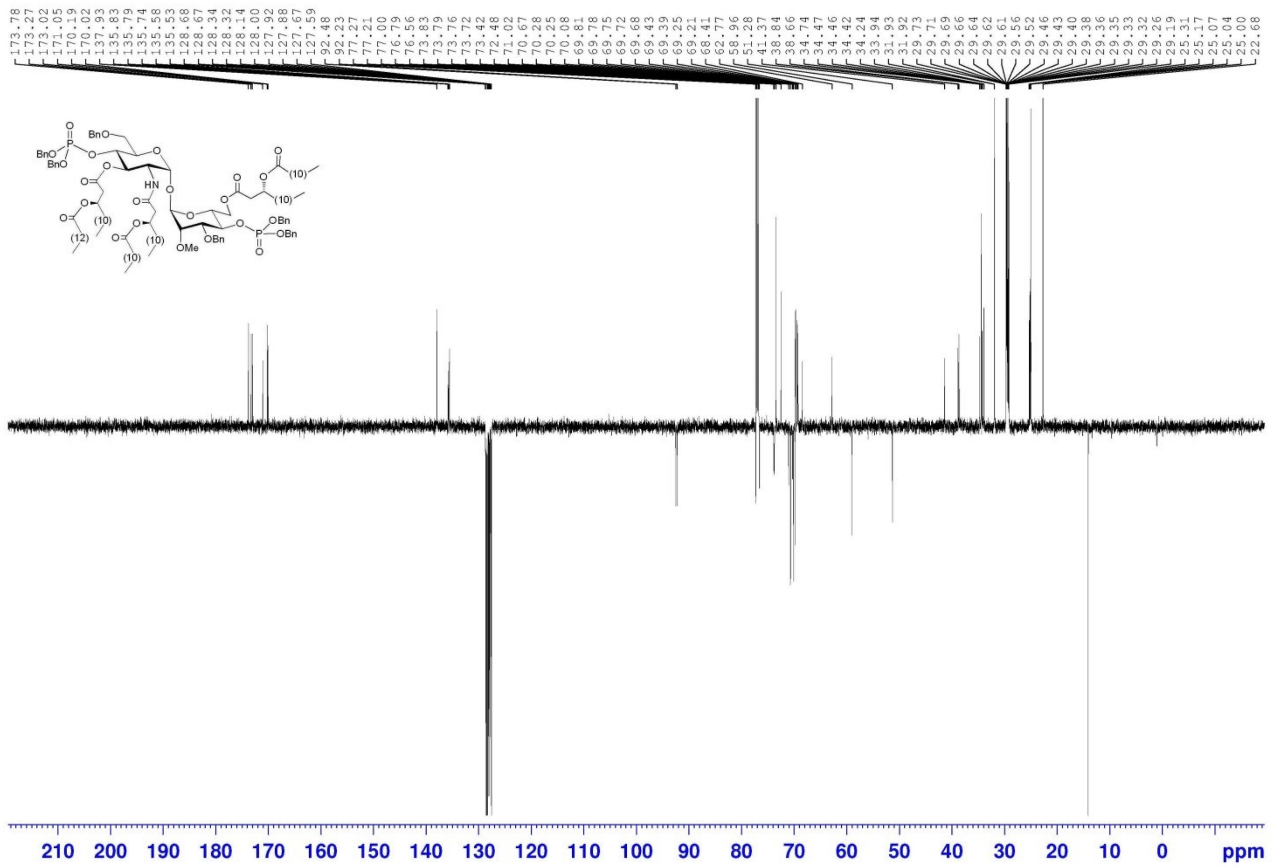

(27): ^{1}H-NMR, 600 MHz, CDCl$_3$

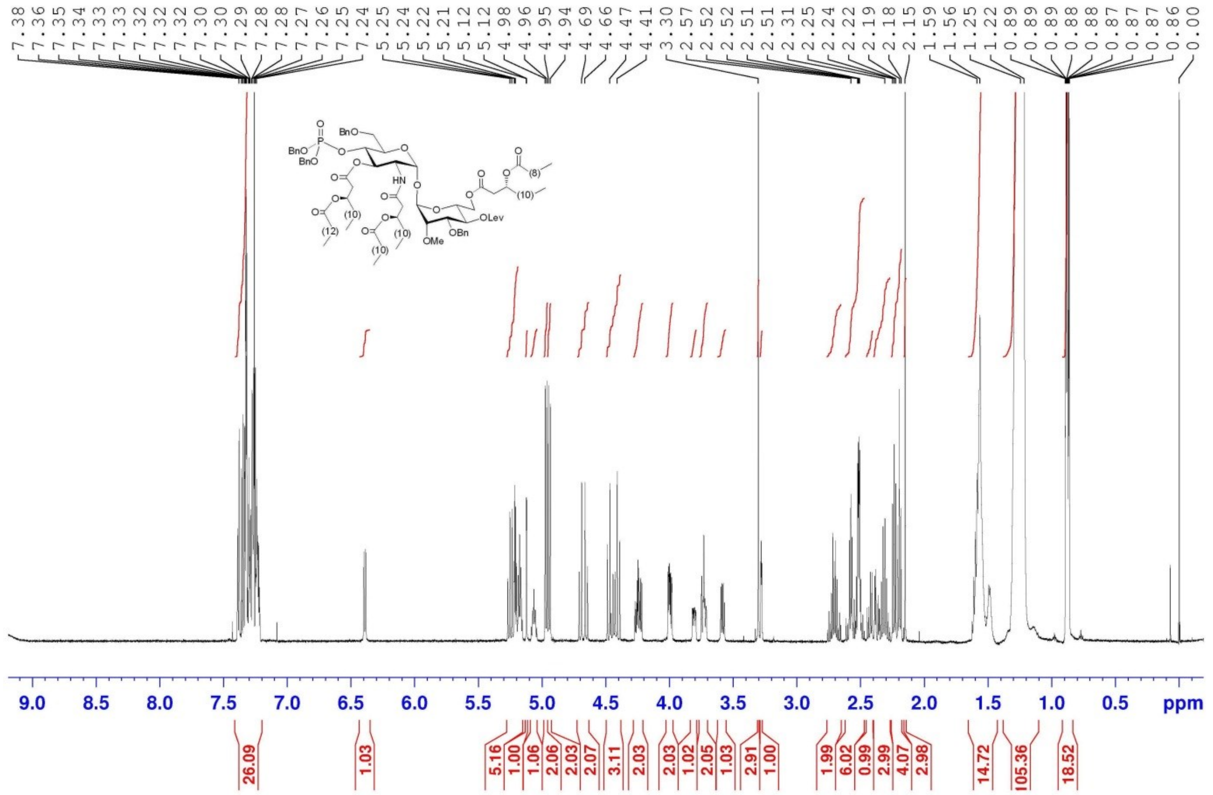

(27): ^{13}C-NMR, APT, 151 MHz, CDCl$_3$

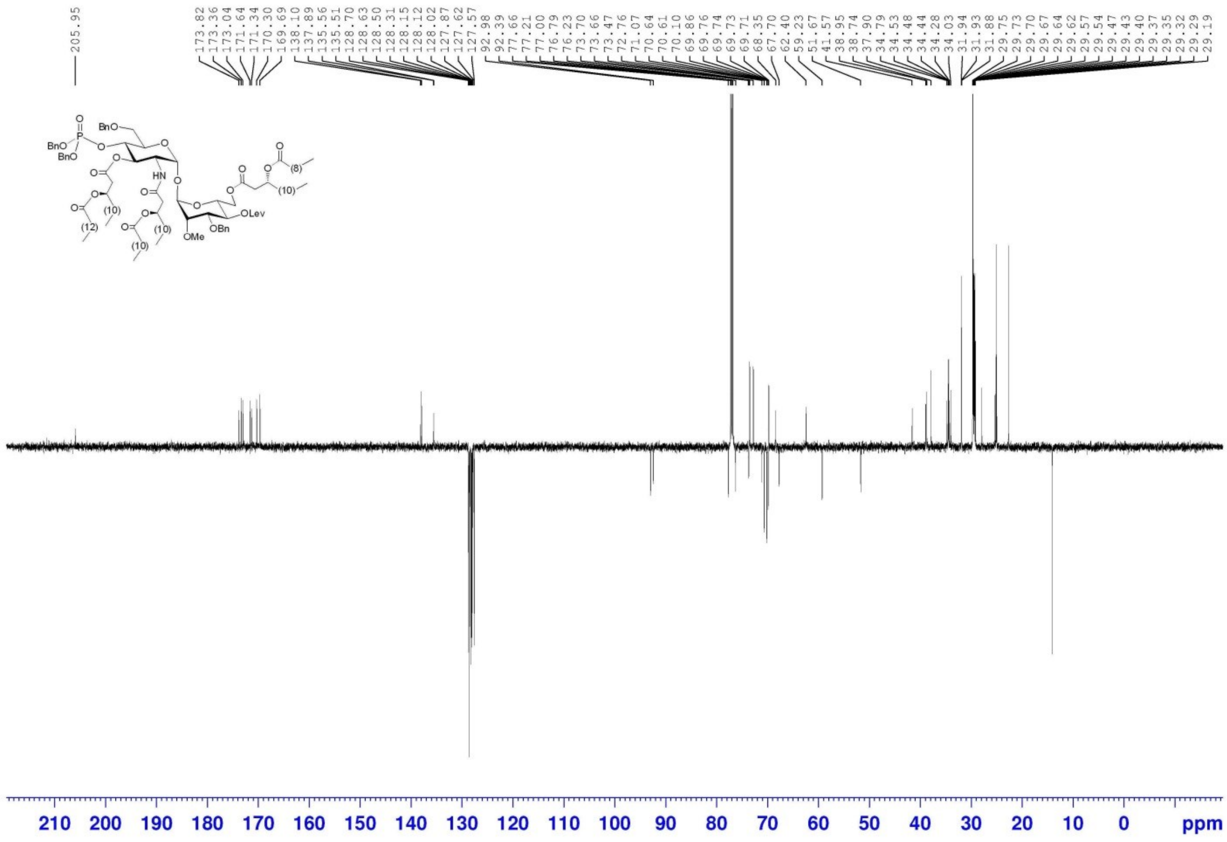

(28): ^{1}H-NMR, 600 MHz, $CDCl_3$

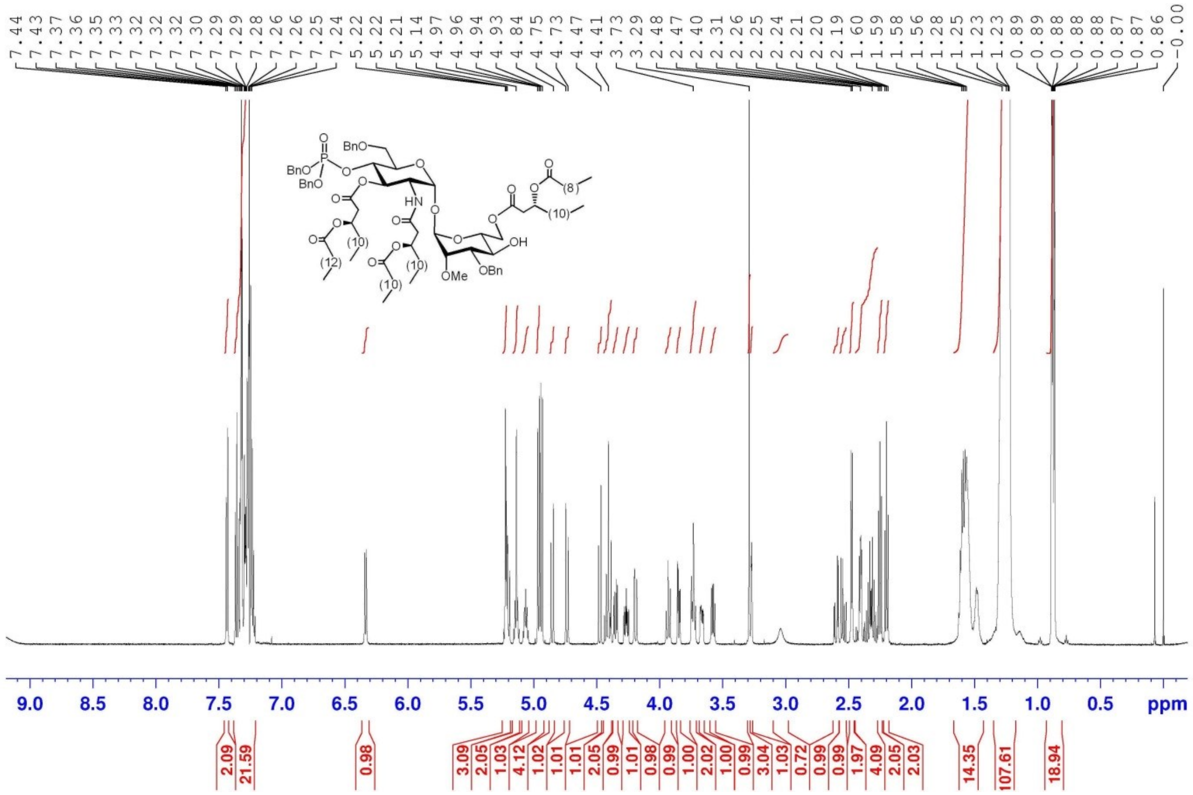

(28): ^{13}C-NMR, APT, 151 MHz, $CDCl_3$

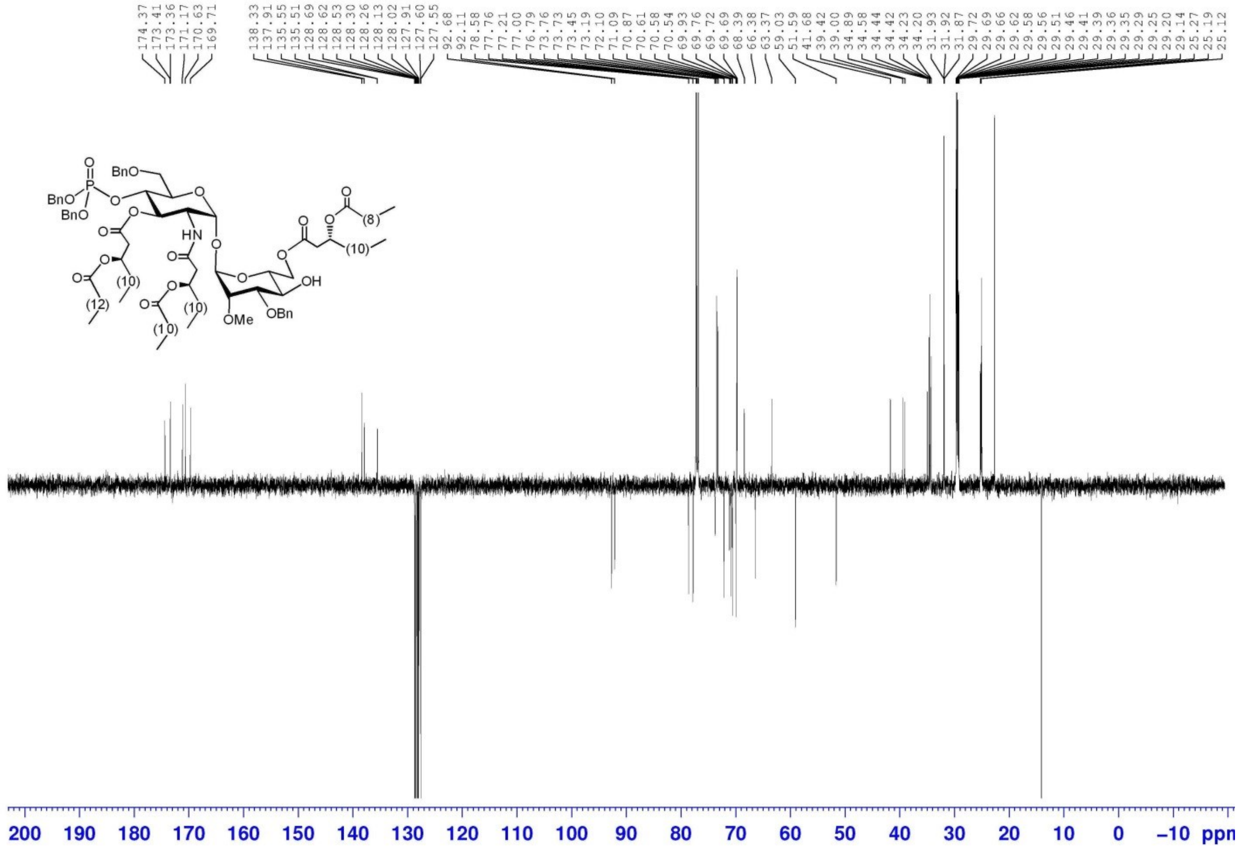

(29): ^{1}H-NMR, 600 MHz, $CDCl_3$

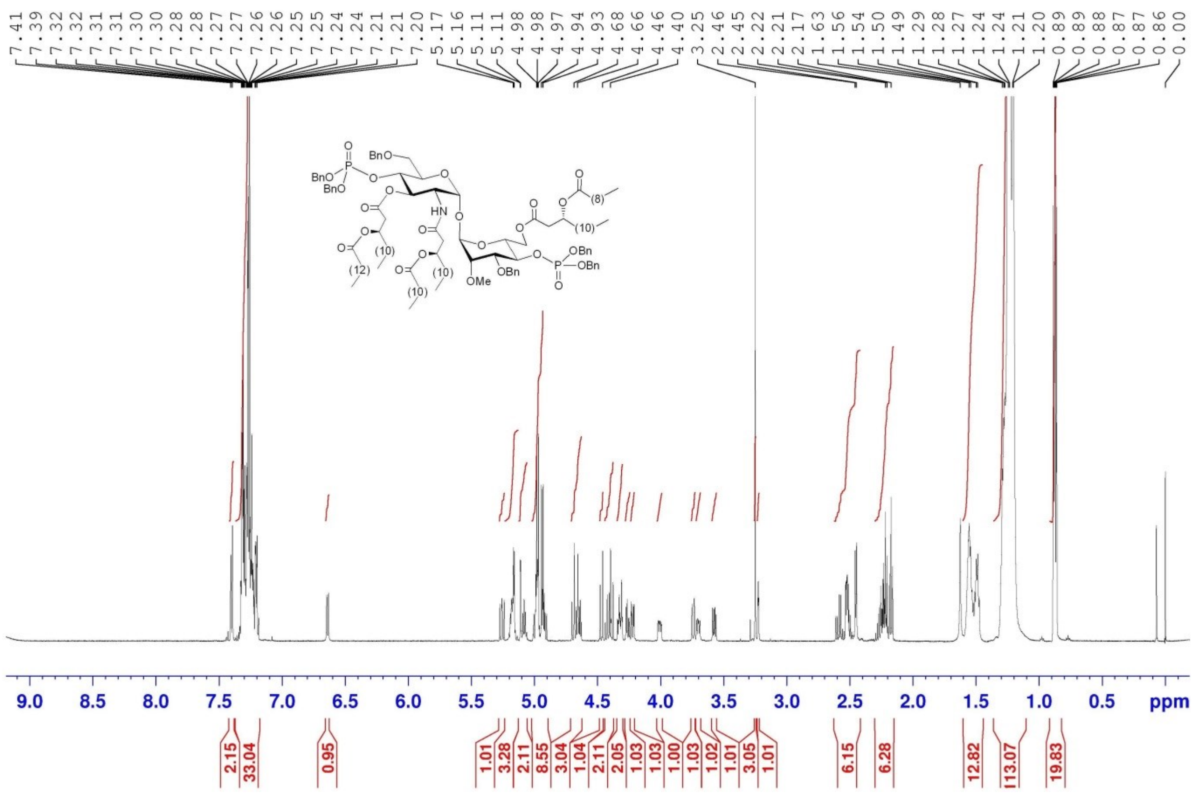

(29): ^{13}C-NMR, APT, 151 MHz, $CDCl_3$

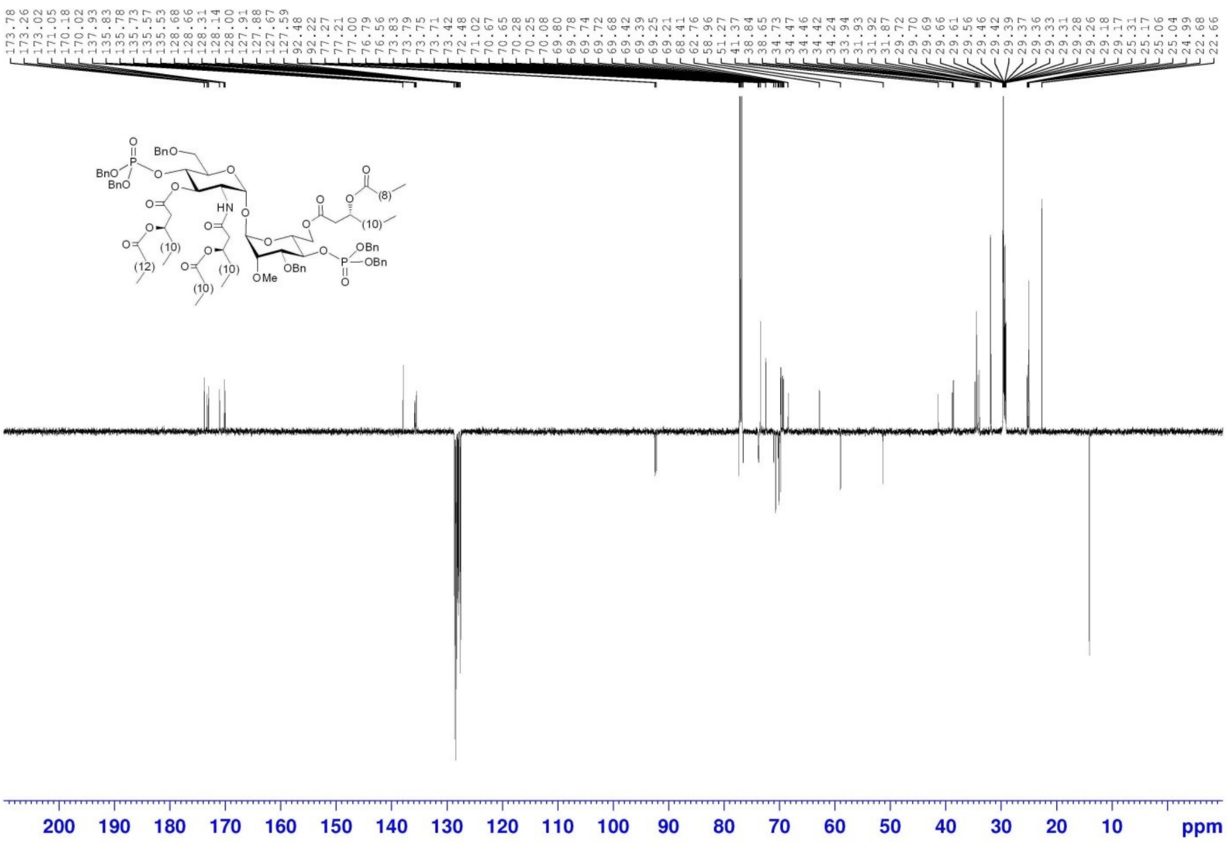

(30): ^{1}H-NMR, 600 MHz, $CDCl_3$

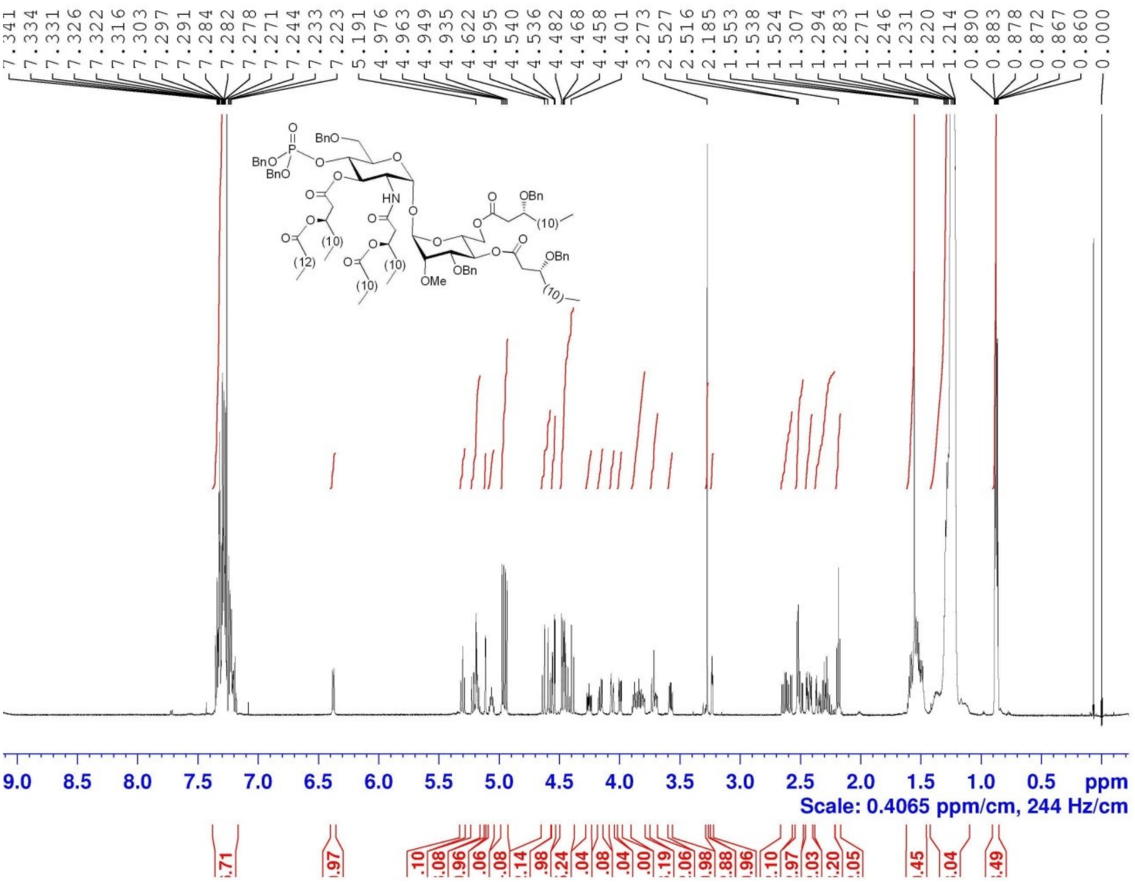

(30): ^{13}C-NMR, APT, 151 MHz, $CDCl_3$

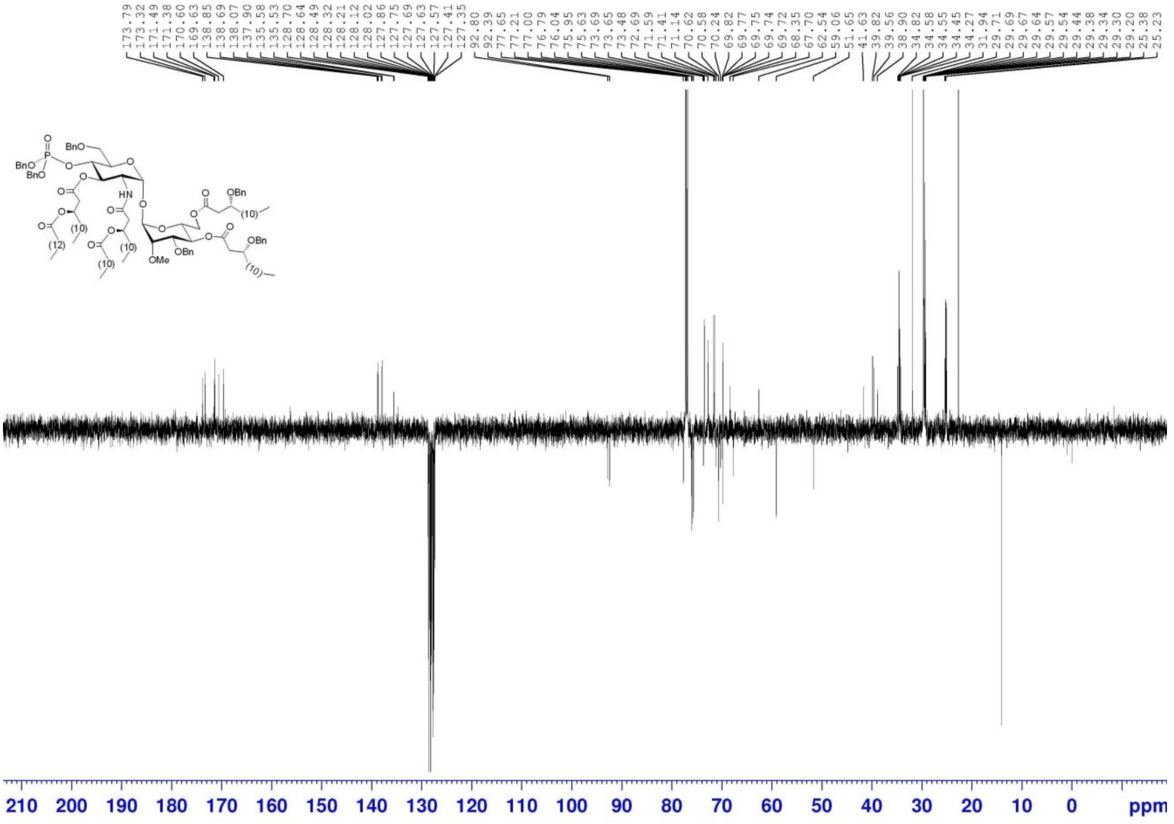

(4): ^{1}H-NMR, 600 MHz, $CDCl_3$ – MeOD, 3 : 1

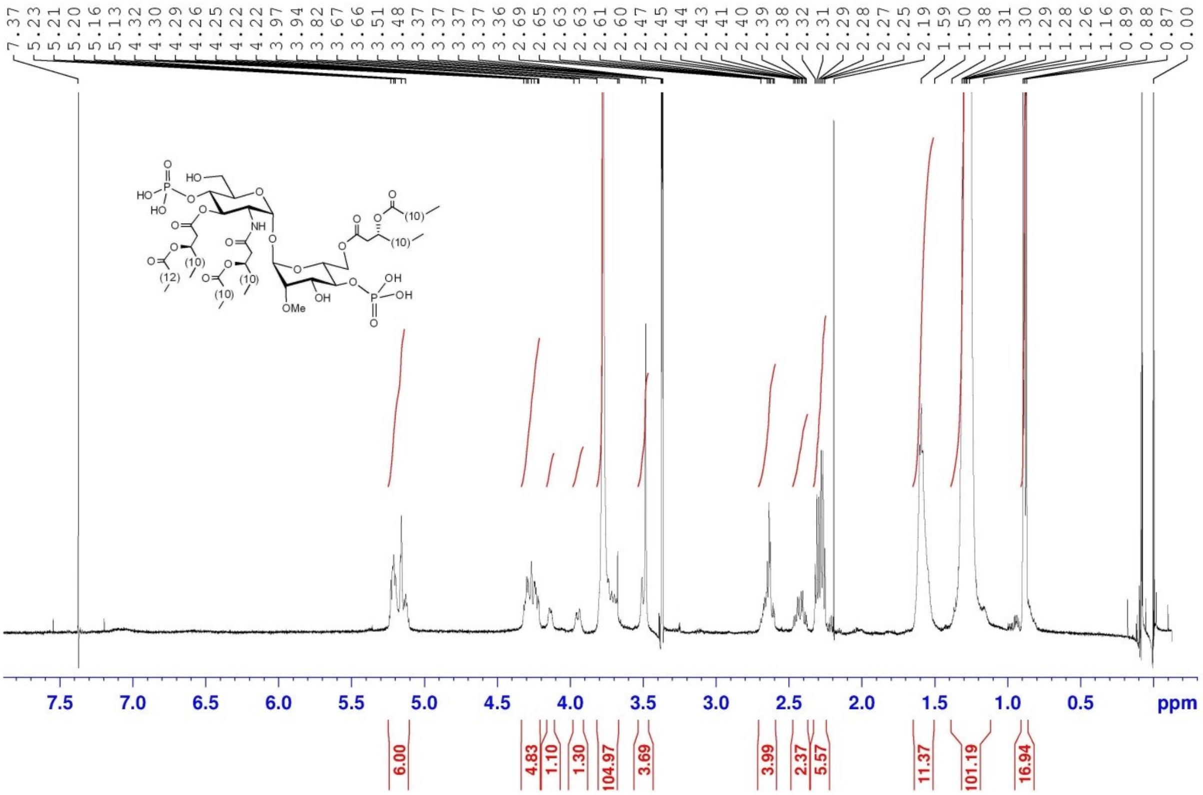

(4): HSQC, $CDCl_3$ – MeOD, 3 : 1

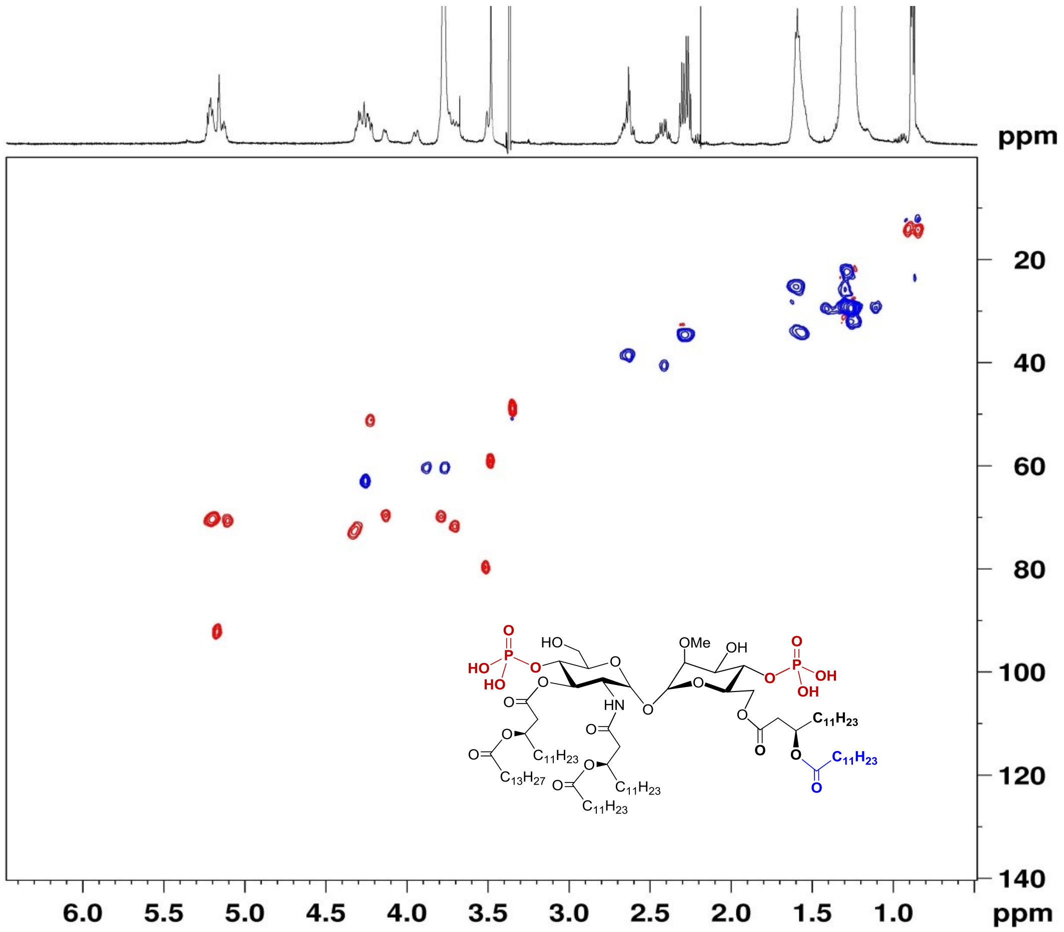

MALDI-TOF (neg. mode) spectrum of **4**

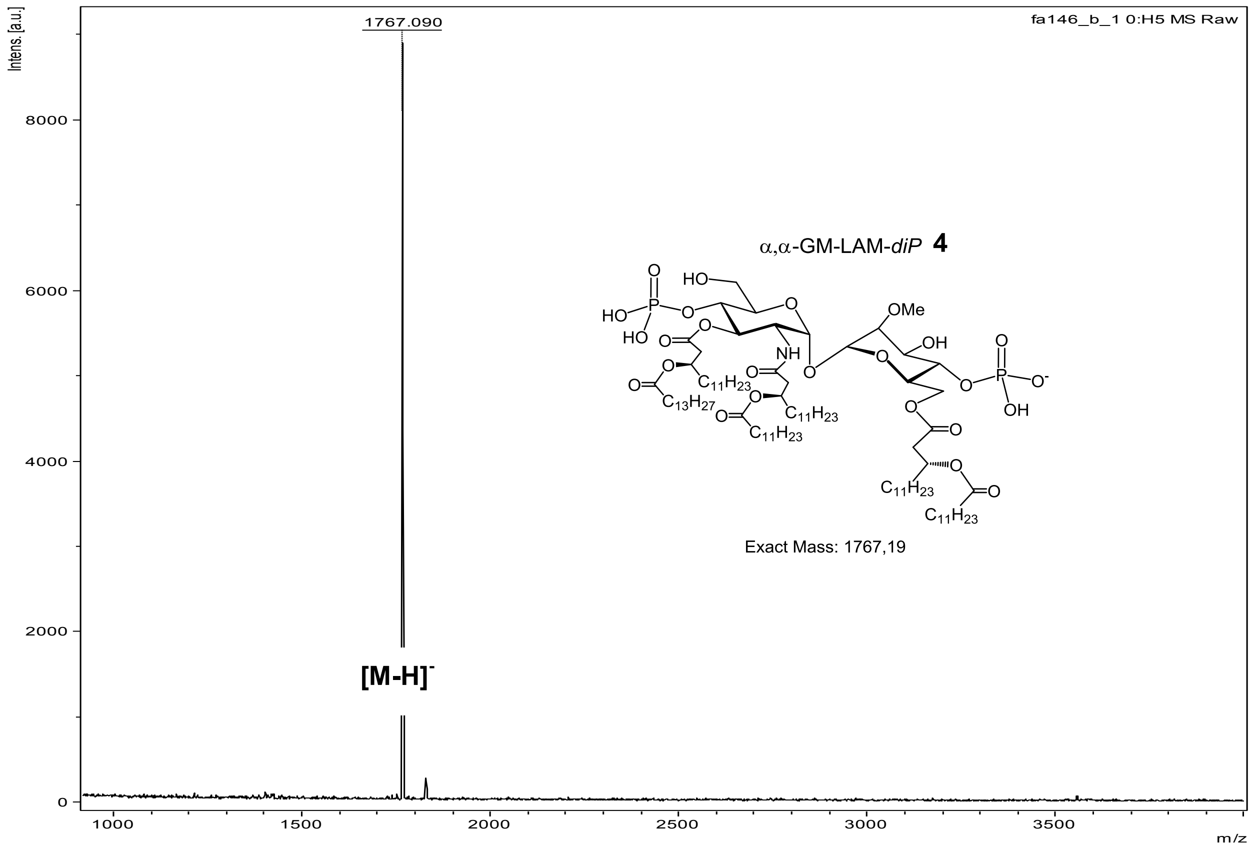

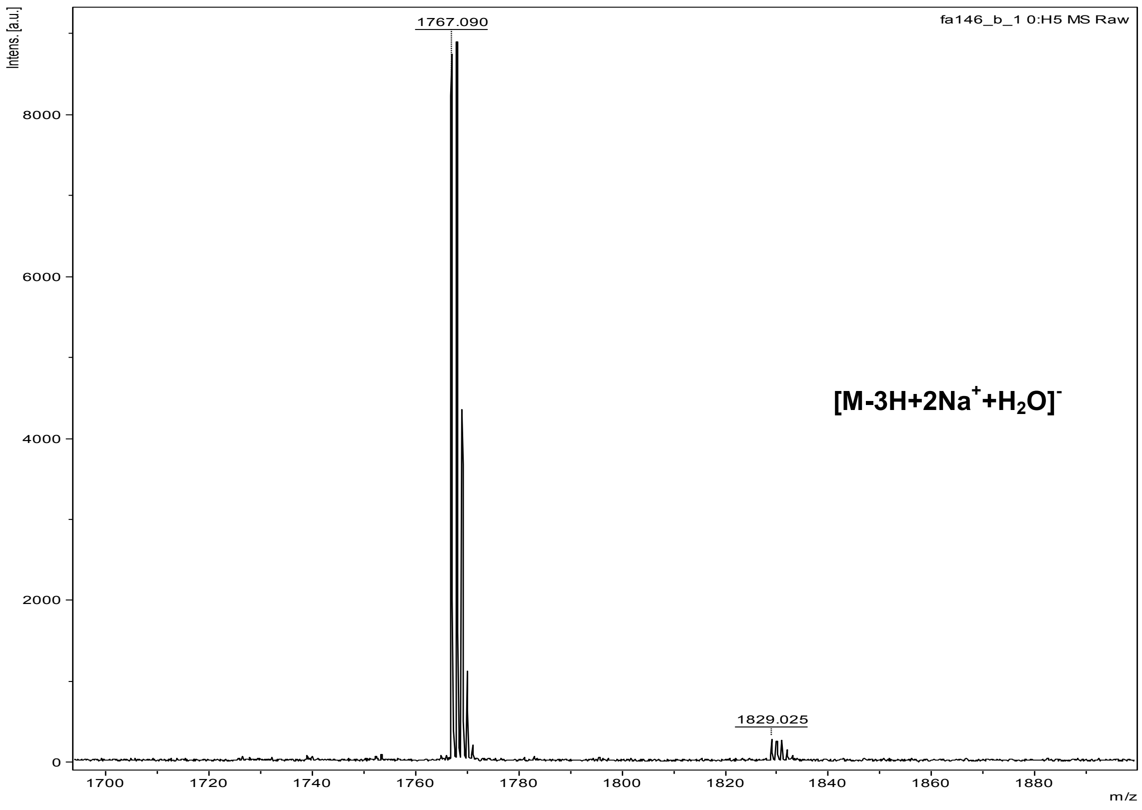

(5): ^{1}H-NMR, 600 MHz, $CDCl_3$ – MeOD, 3 : 1

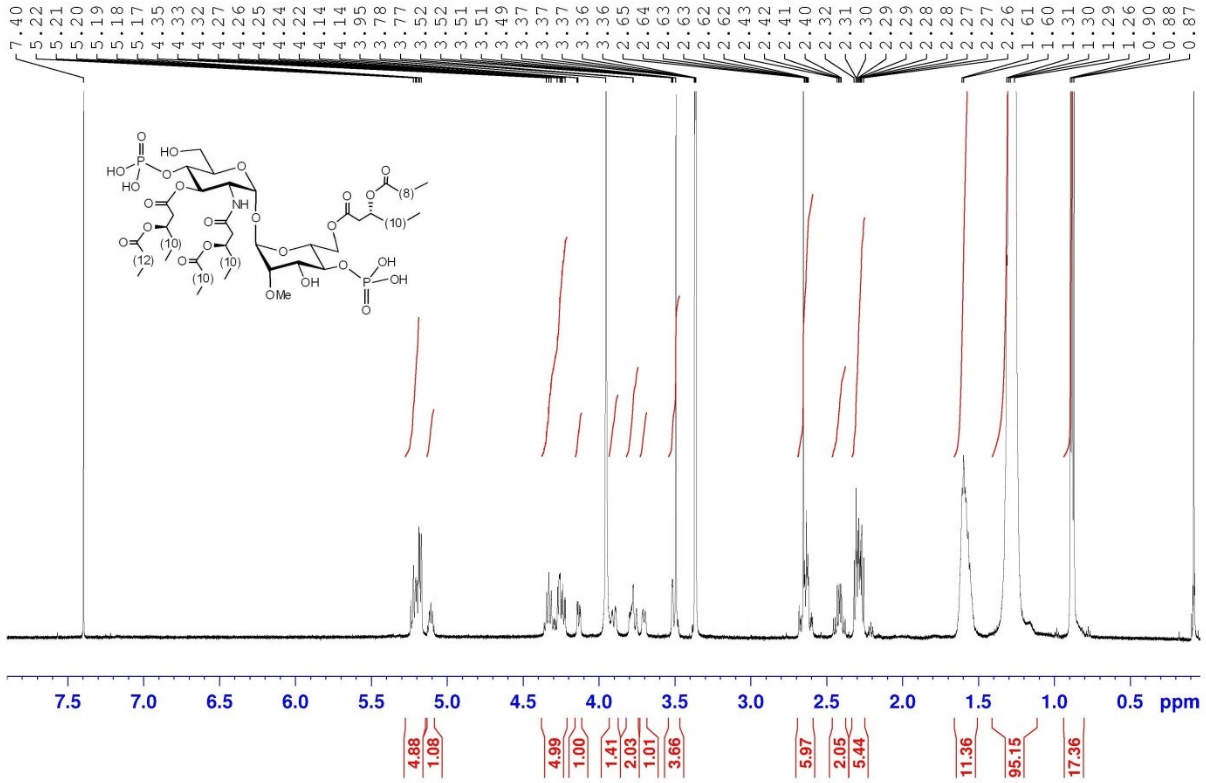

(5): HSQC, $CDCl_3$ – MeOD, 3 : 1

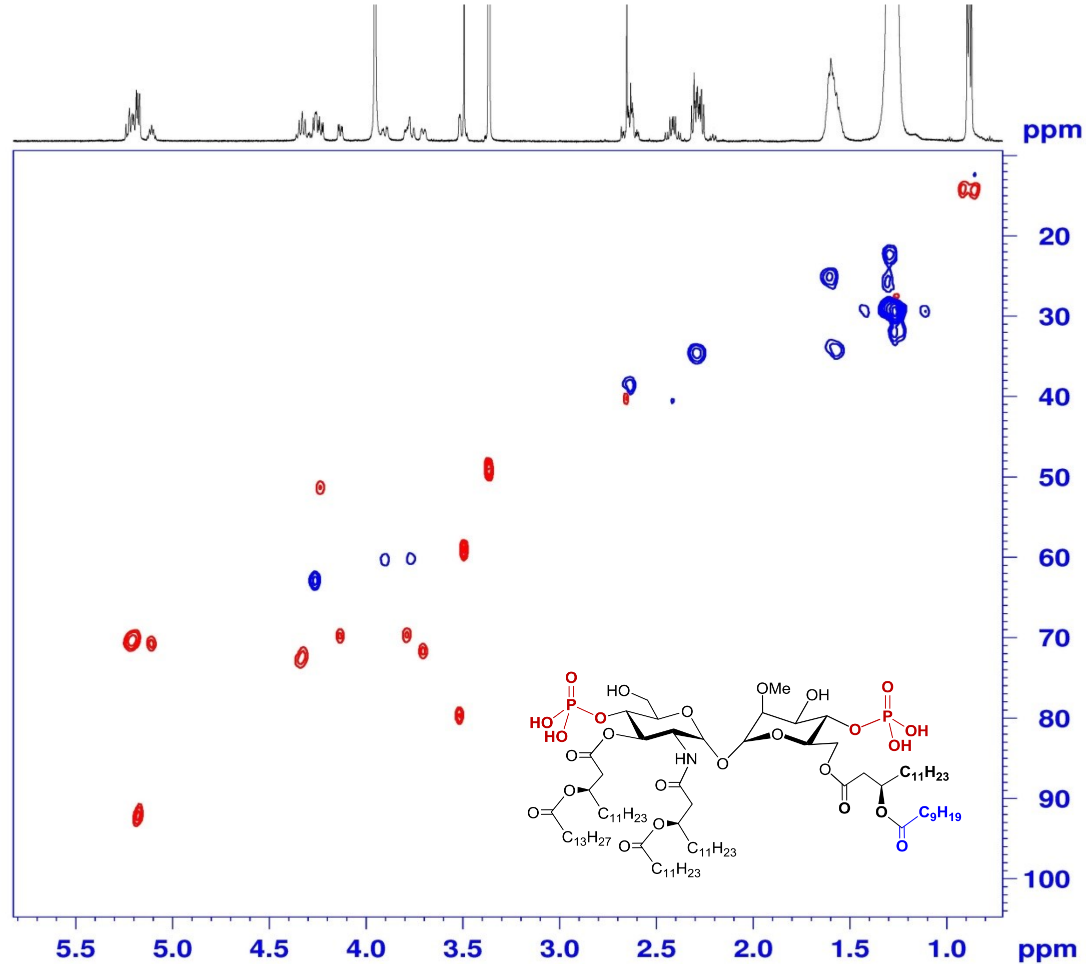

MALDI-TOF (neg. mode) spectrum of α,α-GM-LAM-*diP* **5**

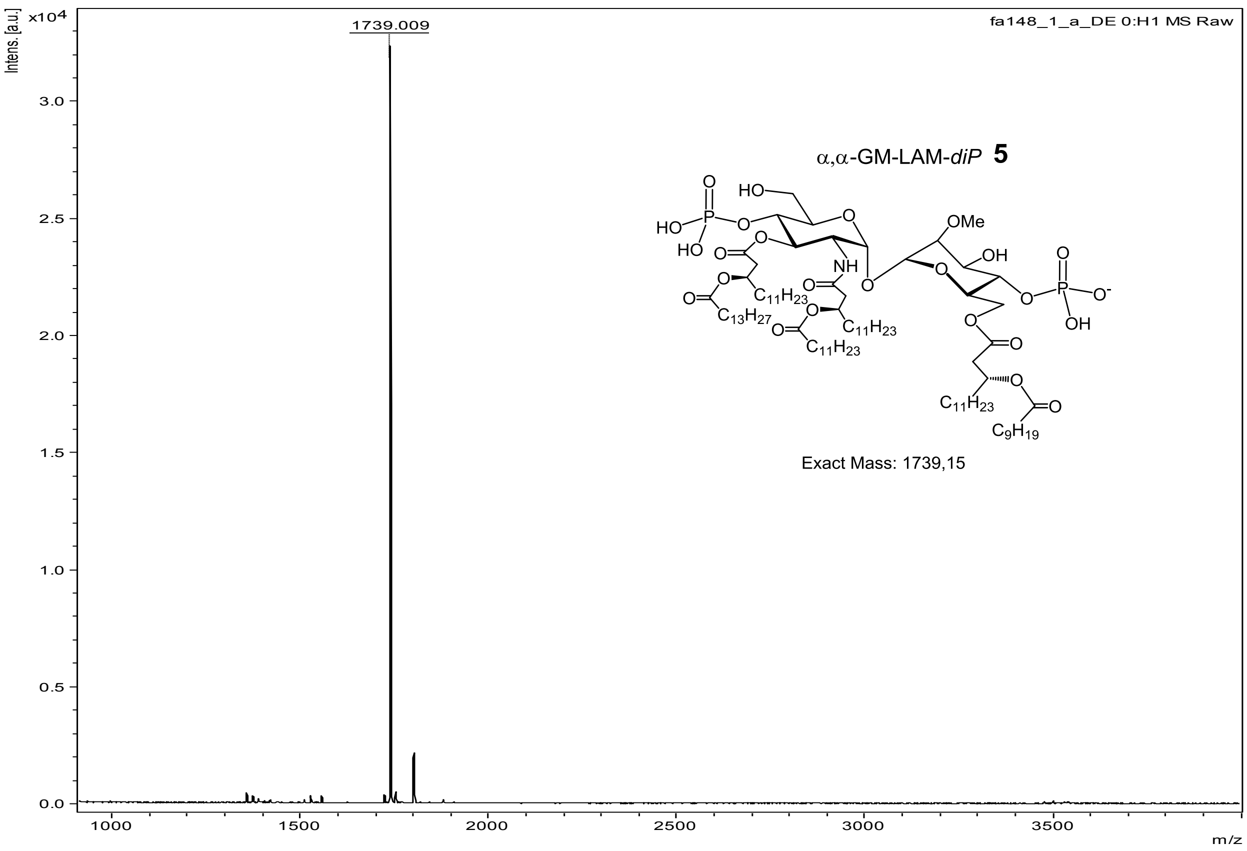

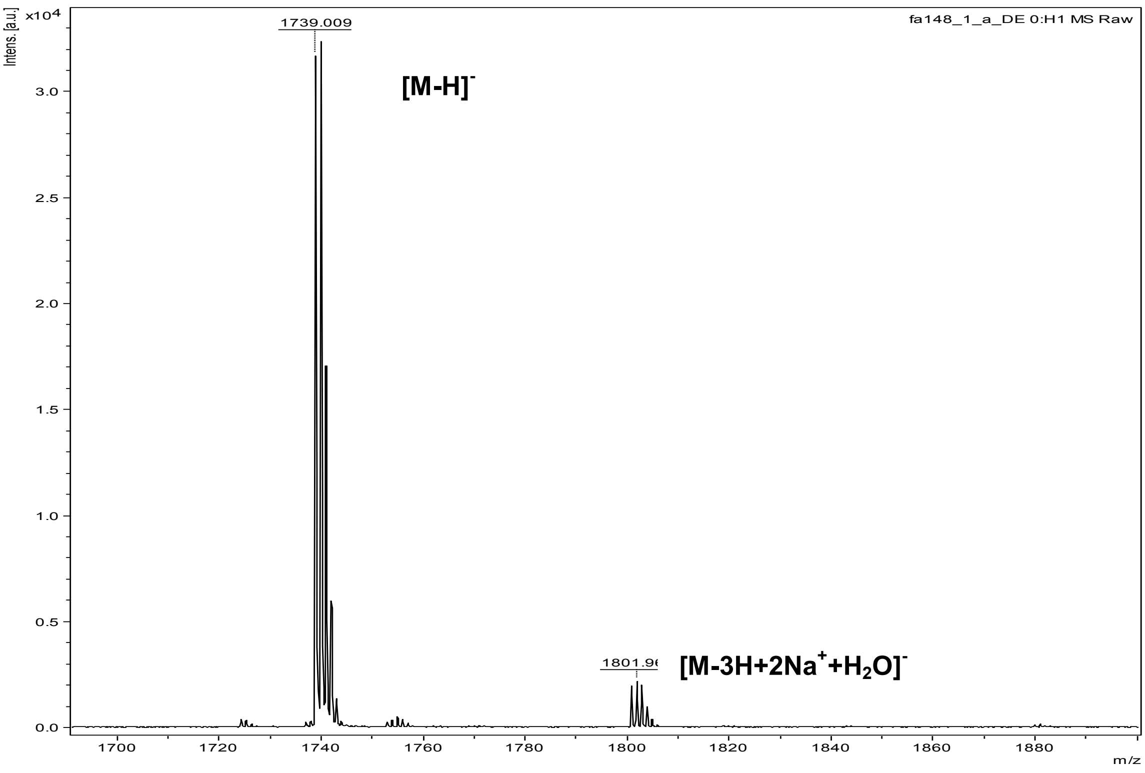

(6): ^{1}H-NMR, 600 MHz, $CDCl_3$ – MeOD, 3 : 1

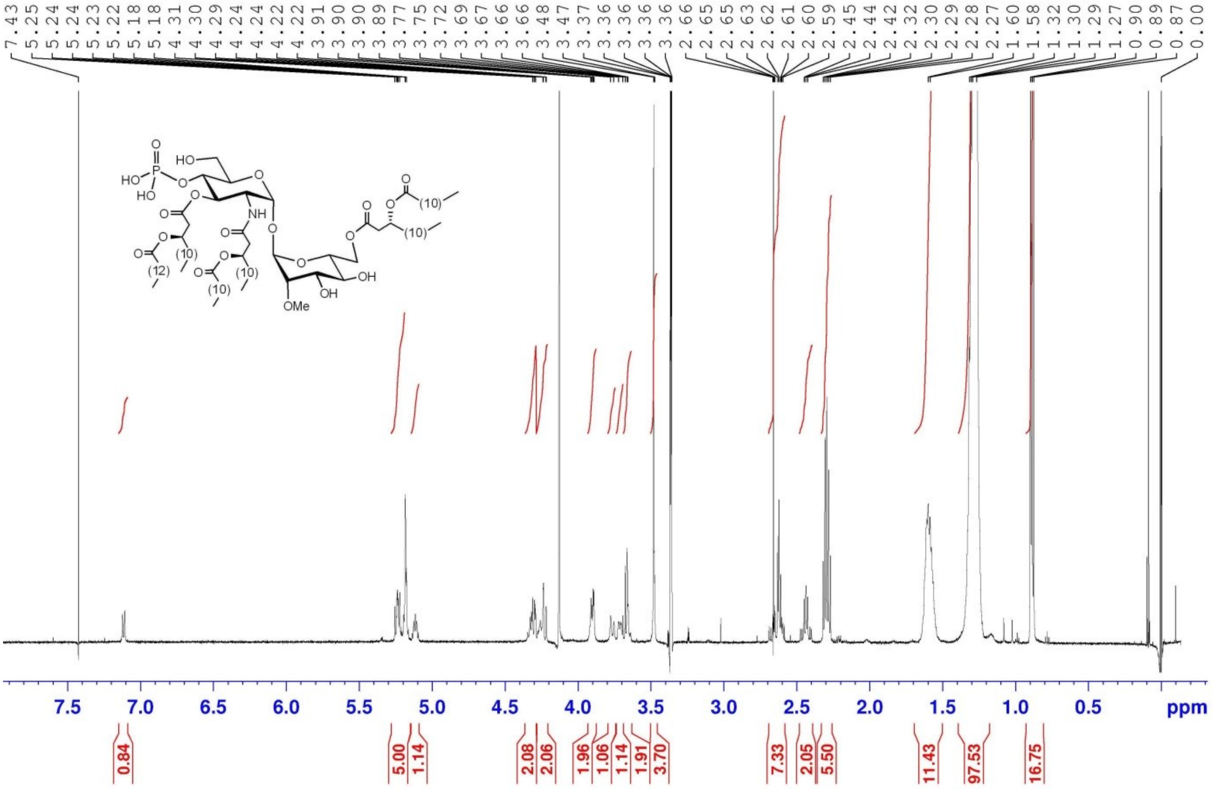

(6): HSQC, $CDCl_3$:MeOD, 3:1

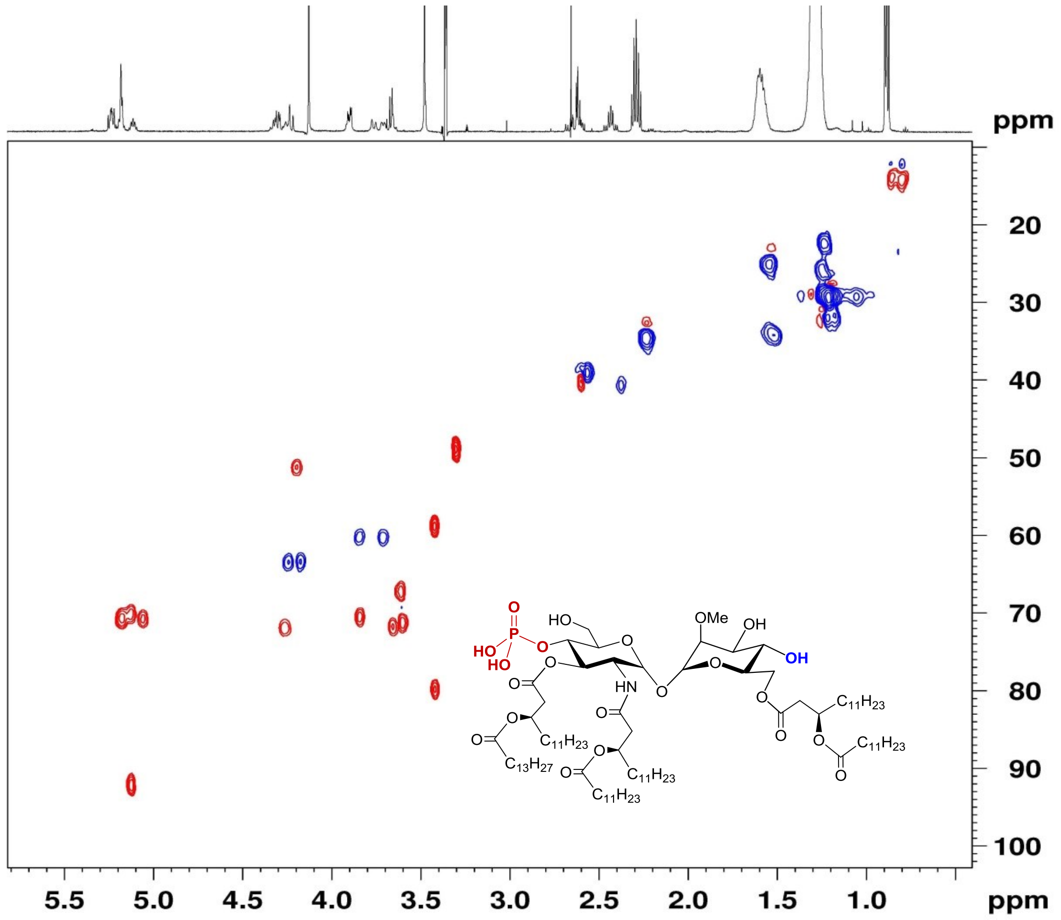

MALDI-TOF (neg. mode) spectrum of **6**

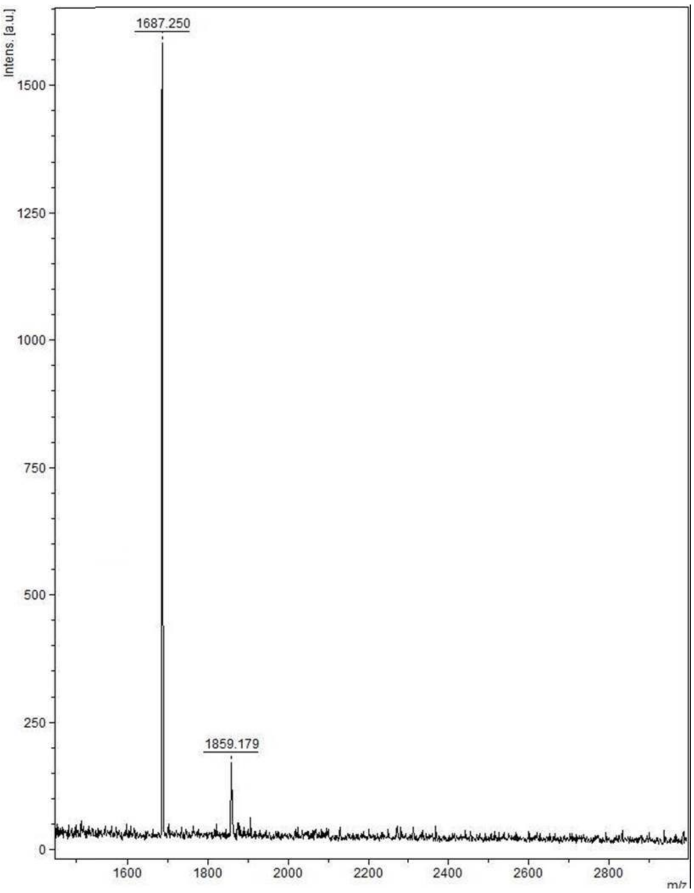

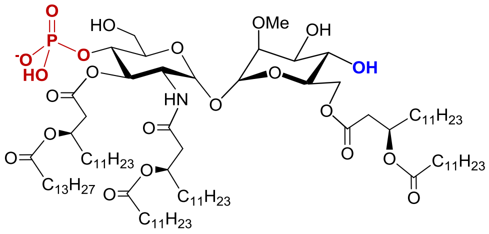

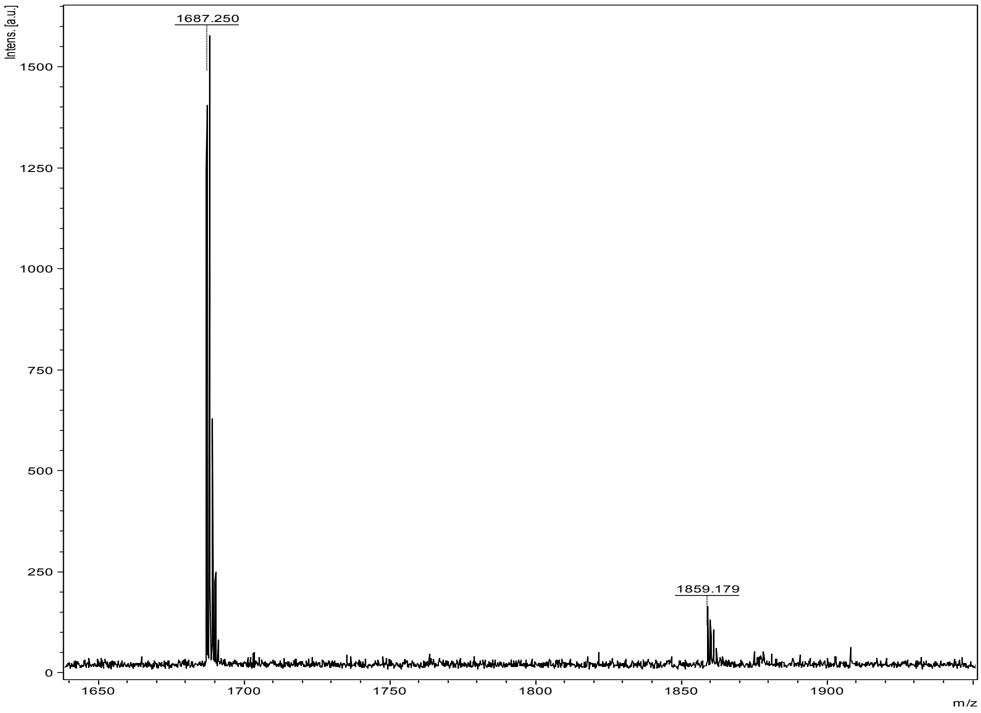

(7): ^{1}H-NMR, 600 MHz, $CDCl_3$ – MeOD, 3 : 1

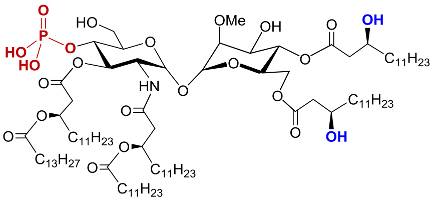

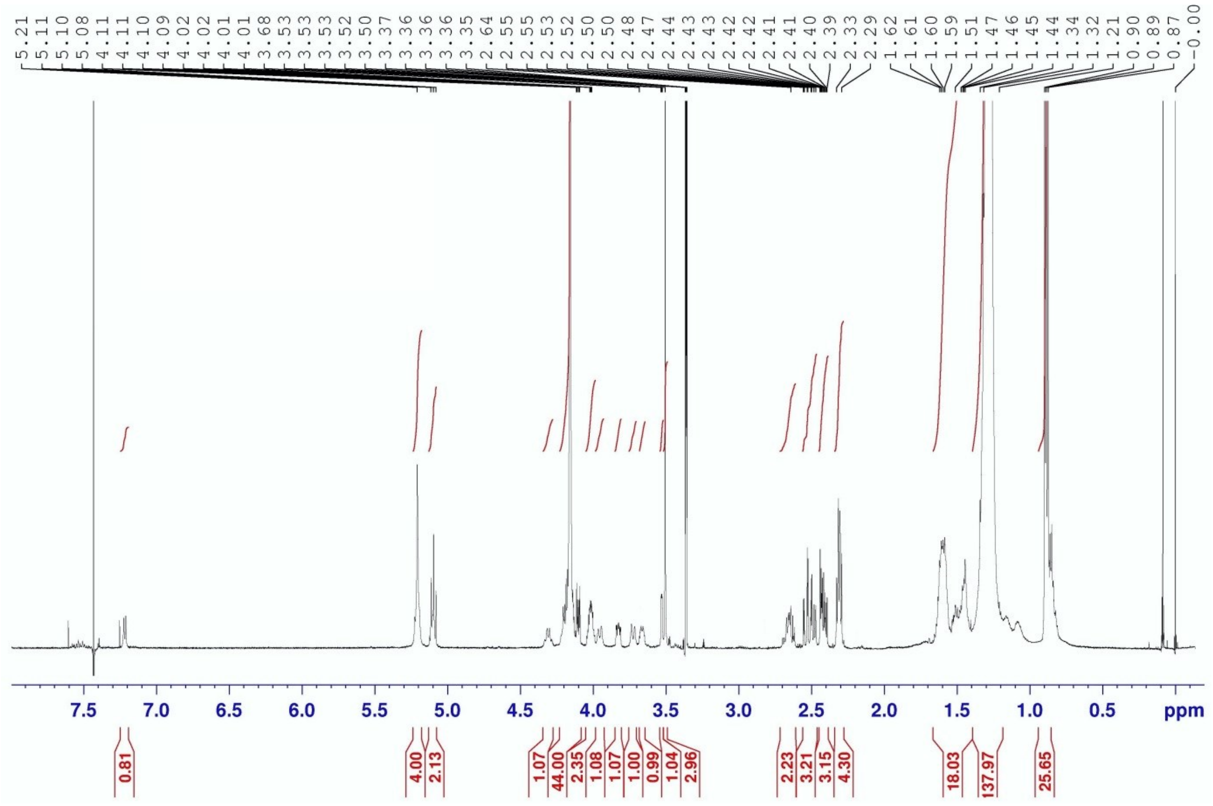

(7): HSQC, $CDCl_3$:MeOD, 3:1

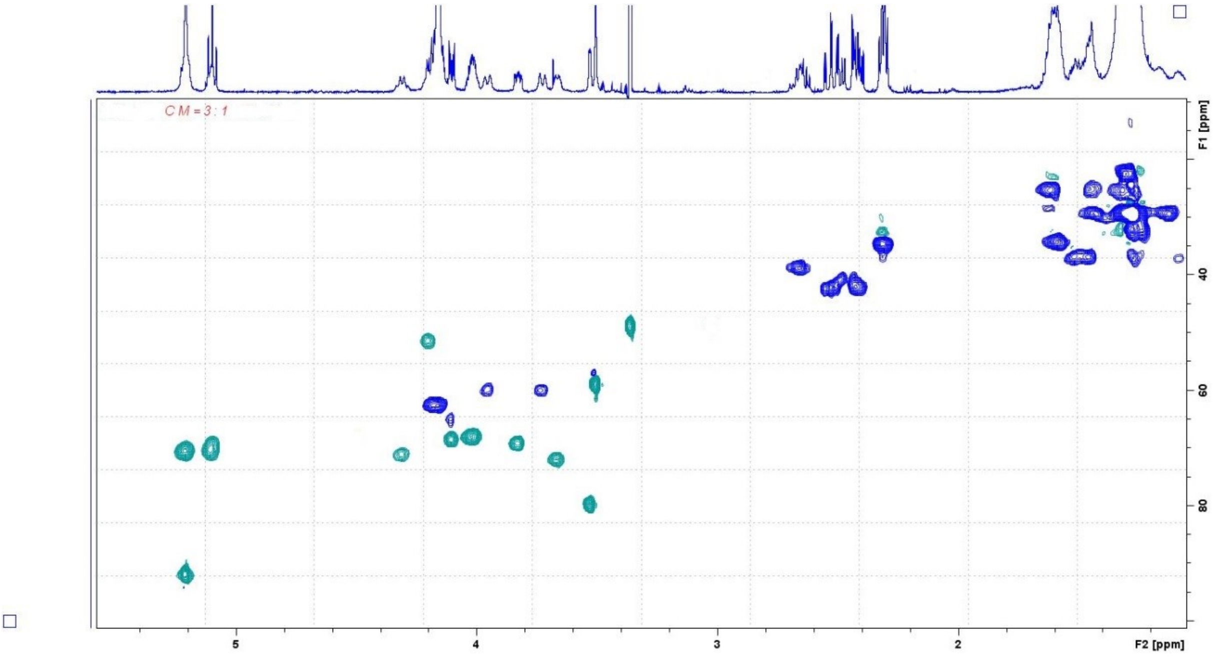

MALDI-TOF (neg. mode) spectrum of **7**

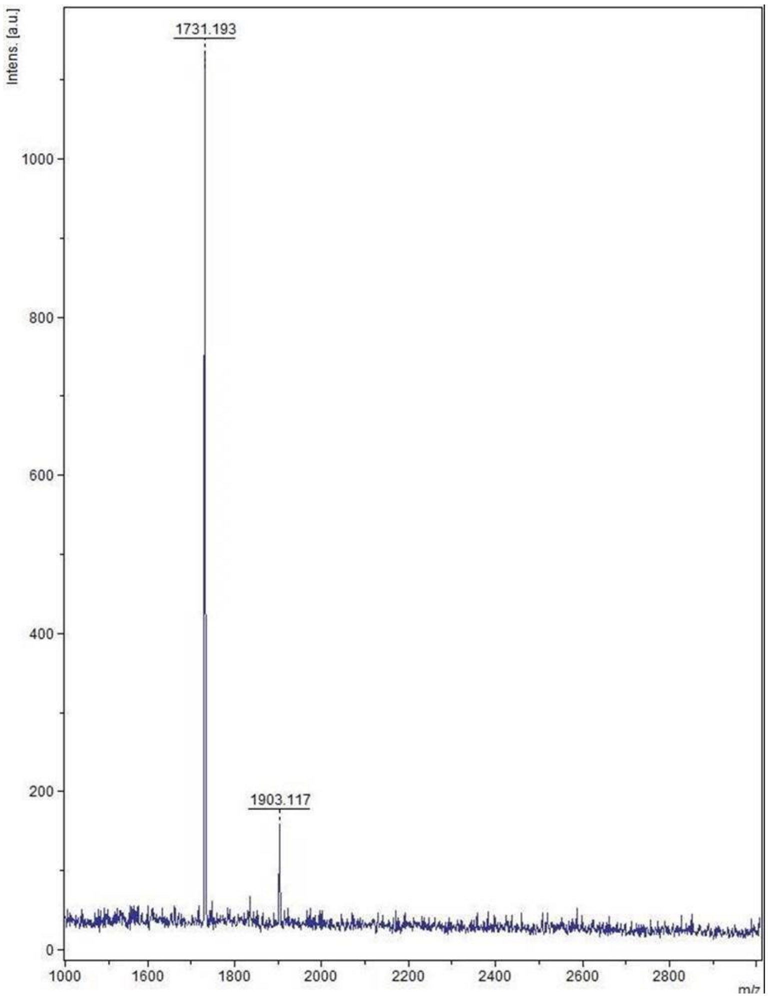

α,α-GM-LAM-*monoP* **7**

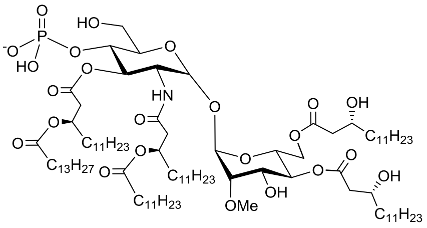

Exact Mass: 1731,25

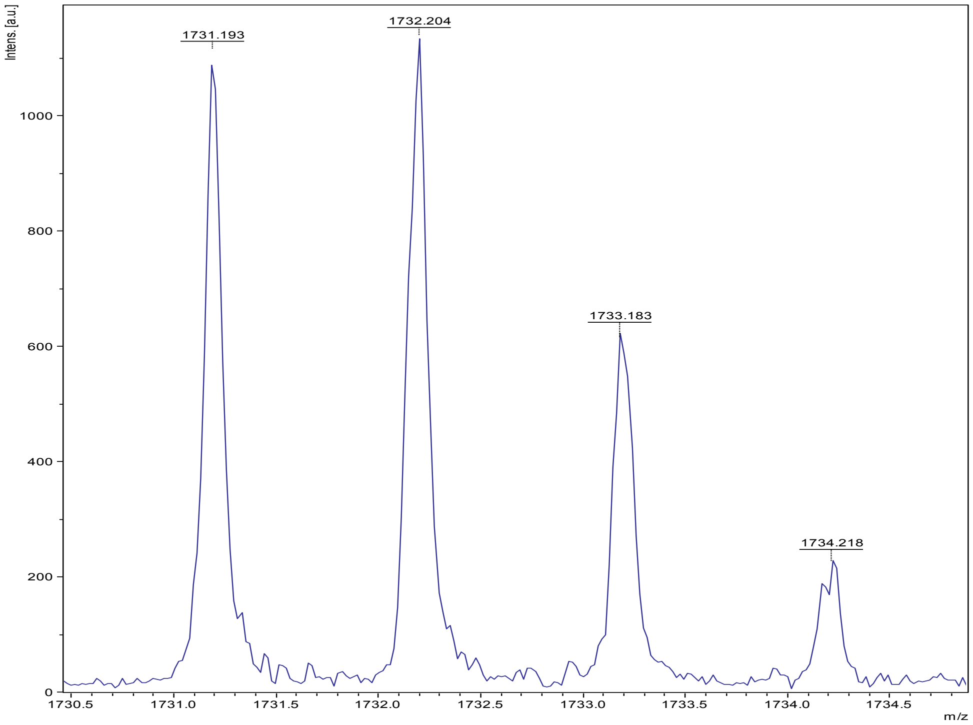

Supplement: Supplementary file 1 [file SC-009-C7SC05323A-s001.pdf]
